# A golden opportunity: benzofuranone modifications of aurones and their influence on optical properties, toxicity, and potential as dyes

**DOI:** 10.3762/bjoc.15.171

**Published:** 2019-07-25

**Authors:** Joza Schmitt, Scott T Handy

**Affiliations:** 1 Department of Chemistry, Middle Tennessee State University, Murfreesboro, TN 37132, USA

**Keywords:** aurone, dyeing, dyes, substitution effect, toxicity, UV–vis spectrum

## Abstract

Aurones are a small subclass of the flavonoid family known primarily for their unusual structure and the golden yellow color they impart to the flowers of snapdragons and cosmos. Most studies of aurones focus on their range of biological activities, but relatively little has been reported with respect to their optical properties, unlike their aza and thio analogs. What little is known has focused entirely on the influence of the benzylidene portion. In this study, the influence of substitution in the benzofuranone ring on the UV–vis spectrum is explored, as well as an initial screening of their toxicity and a qualitative preliminary study of their potential to act as fabric dyes.

## Introduction

Aurones are a fascinating minor sub-family of the flavonoid natural products [1–2]. While they feature the same C15 composition as other flavonoids, the skeleton is quite different, featuring a benzofuranone connected to an aromatic ring via an exocyclic alkene. This unusual skeleton has attracted a modest amount of synthetic attention and fairly recently significant biological focus. At the same time, aurones were first noted (and indeed their name is derived from) for their golden yellow color. The colors of flavonoids in general have been appreciated and used for virtually the entirety of recorded history and yet the application of aurones as dyes or pigments has not been reported or studied [3].

Even the optical properties of aurones have had very minimal study. The reports that have appeared have all focused on fluorescent properties and have also been largely limited in scope to the influence of the benzylidene portion. Most noteworthy is the report by Bane and co-workers examining the UV–vis and fluorescent properties of a series of amino-substituted aurones [4]. Subsequently, Liu and co-workers explored the same series of aurones using computational methods to develop and validate the method for the rational design of new aurone fluorophores [5].

**Figure 1:**
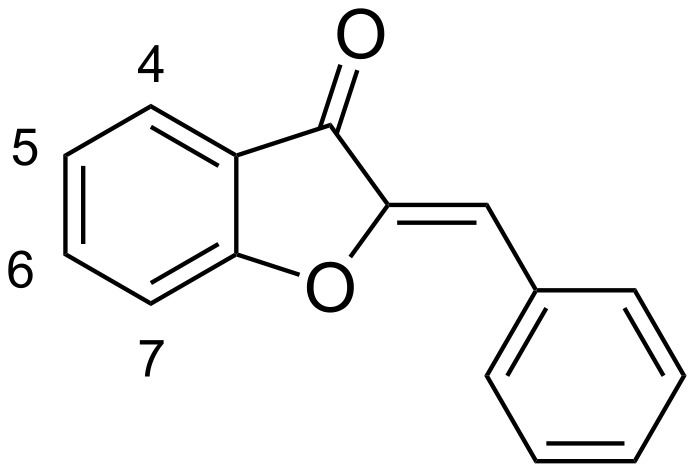
Aurone ring system and numbering.

Two more recent studies have explored the influence of the benzofuranone portion of the aurone system. Salas examined a larger set of compounds featuring methoxy substitution at 4 or 5-position on the benzofuranone and methoxy moiety and one methoxy/bromo-substituted benzylidene group, but none in which the benzofuranone was unsubstituted (see Figure 1 for numbering) [6]. This meant that no comparison regarding the influence of substitution could realistically be made. They did observe that a methoxy group at the 5-position resulted in a shift to longer wavelength by roughly 20 nm compared to a methoxy substituent at the 4-position and a strong dependence upon solvent polarity. While this shift was quite reasonably attributed to the inductive effect of the oxygen, no other substitution was studied. Muñoz-Becerra and co-workers have also reported a computational study of amino aurone derivatives with variations in the benzofuranone portion of the molecule, though all substitution was strictly at the 4-position of the benzofuranone [7].

As an extension of our on-going studies of the aurone ring system, we chose to study the potential for aurones to be used as dyes as well as a more extensive study of the impact of substitution in the benzofuranone portion of the molecule on its optical properties.

## Results and Discussion

### Synthesis

**Figure 2:**
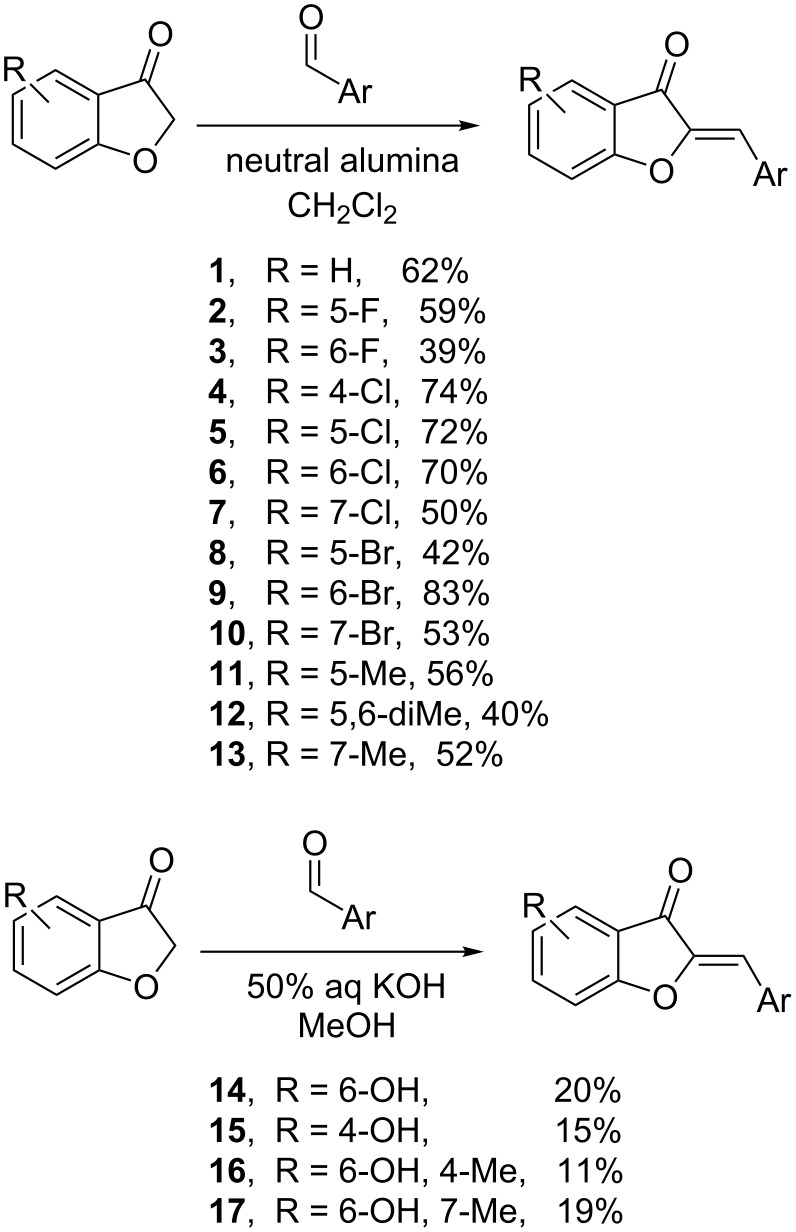
Aurone syntheses.

We have previously reported the synthesis of a significant number of new aurones by way of the common Knoevenagel condensation approach, mostly varying in the benzylidene portion. To explore benzofuranone variations using this method, different benzofuranone starting materials are required. Although not likely to be the most colorful, we elected for simplicity’s sake to use *p*-tolualdehyde as the benzylidene precursor for all of the new aurones. The synthesis itself was performed via one of two methods (Figure 2). For all compounds without a free hydroxy group, the neutral alumina method of Varma combined with an aldehyde scavenging step was employed to afford pure products without the need for any chromatography in generally reasonable yields and excellent purity [8]. For hydroxy-substituted compounds, a more traditional base-mediated reaction was employed [9]. Product purification was not as easy in these cases and the product yield was sacrificed for the sake of high purity, so yields should not be considered optimized.

### Spectral and toxicity studies

**Figure 3:**
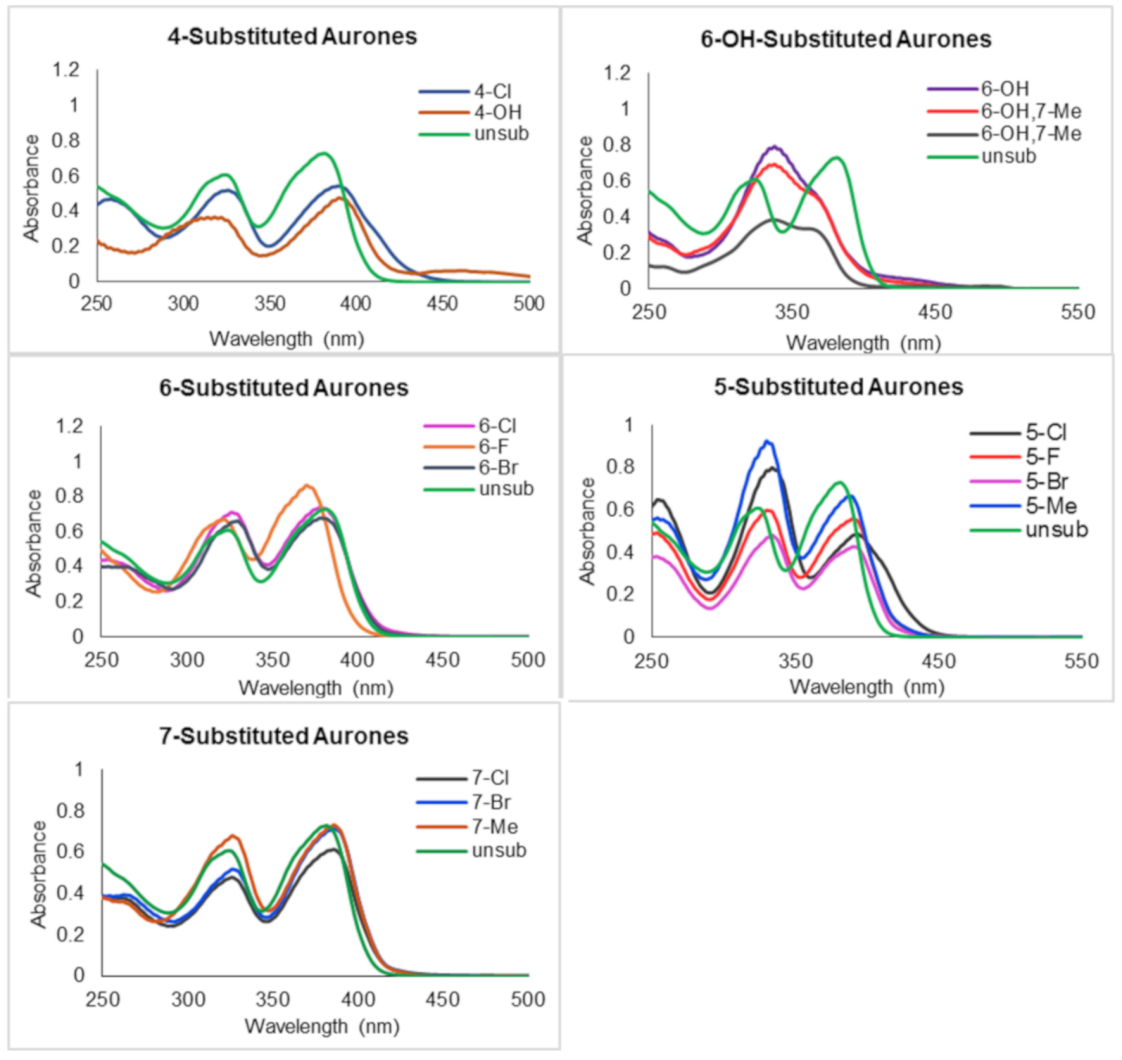
UV–vis spectral comparisons in acetonitrile.

With the desired compounds in hand, their UV–visible spectra were recorded at concentrations between 29 and 44 μM in acetonitrile (Figure 3 and Table 1). While not all substituents were prepared at every position, some interesting trends were observed. Substitution at the 4-position whether halogen or hydroxy afforded essentially identical lambda maxima (390 nm), slighly red-shifted compared to the unsubstituted compound **1**. On the other hand, hydroxy groups at the 6-position result in a significant blue shift of this lambda max by roughly 40 nm to around 338 nm, presumably due to their donating effect and conjugation with the carbonyl oxygen. Halogen substituents at the 6-position generally had little effect, with the exception of the most electron-withdrawing fluorine, which resulted in a slight blue-shift of roughly 10 nm. Unexpectedly, any halogen at the 5-position had an effect nearly identical to that of halogen at the 4-position, resulting in a slight red-shift compared to the unsubstituted compound. On the other hand, substitution at the 7-position had virtually no effect, which is to be expected due to the lack of direct conjugation with the carbonyl as well as the only modest electronic effects of alkyl, chlorine, and bromine. In general, the substituent effects are all fairly modest and result in fairly moderate changes in the extinction coefficient (although hydroxy substitution does definitely increase this value by roughly 50%).

**Table 1:** Toxicity and UV spectral data for aurones.

Compound	Substituent	λ_max_ (nm)^a^	ε (L mol^−1^ cm^−1^)	Hep G2 % inhibition^b^

**1**	H	382	20208	86.27 ± 1.09
**2**	5-F	394	17188	87.01 ± 0.56
**3**	6-F	370	24252	3.52 ± 3.39
**4**	4-Cl	390	17129	15.13 ± 5.75
**5**	5-Cl	394	16551	72.68 ± 2.77
**6**	6-Cl	378	23187	25.07 ± 18.77
**7**	7-Cl	386	17247	3.64 ± 3.64
**8**	5-Br	394	15112	59.35 ± 1.25
**9**	6-Br	378	22435	75.22 ± 3.84
**10**	7-Br	390	22250	5.85 ± 11.12
**11**	5-Me	390	15470	80.88 ± 1.38
**12**	5- and 6-Me	382	19522	24.95 ± 2.23
**13**	7-Me	386	18380	87.54 ± 1.61
**14**	6-OH	338	33050	66.79 ± 3.04
**15**	4-OH	390	22664	54.04 ± 4.65
**16**	6-OH, 4-Me	338	30445	67.76 ± 6.06
**17**	6-OH, 7-Me	336	will not dissolve completely	78.58 ± 7.11
tartrazine	N/A	N/A	N/A	6.81

^a^UV–vis spectra determined in CH_3_CN. ^b^Toxicity values determined at 200 μM concentration of compound.

While these results on the impact of the benzofuranone portion of aurones on their optical properties are interesting, if one were to think about using them as dyes, toxicity is also an important consideration. Generally, aurones are considered to be relatively non-toxic, although data reported in the literature shows considerable variability even in this respect. An initial screening of toxicity was conducted at a fairly high concentration (200 μM) on the present series of compounds using a standard HEP G2 inhibition assay (Table 1). Compared to a currently used yellow dye (tartrazine), the aurones are similar to more toxic. Within the aurone series, though, an interesting pair of trends can be observed. First, all hydroxylated aurones are comparatively more toxic, displaying >50% inhibition at 200 μM. Methyl groups are similarly mostly more toxic. For the halogens, however, location is fairly important, with the 6 or 7 position being much less toxic (similar to tartrazine) and dramatically better than the unsubstituted base compound **1**, with the unexpected exception of 7-bromo compound **10**. Whether this trend is general or not for aurone compounds is an interesting question for future study.

### Preliminary dyeing efforts

**Figure 4:**
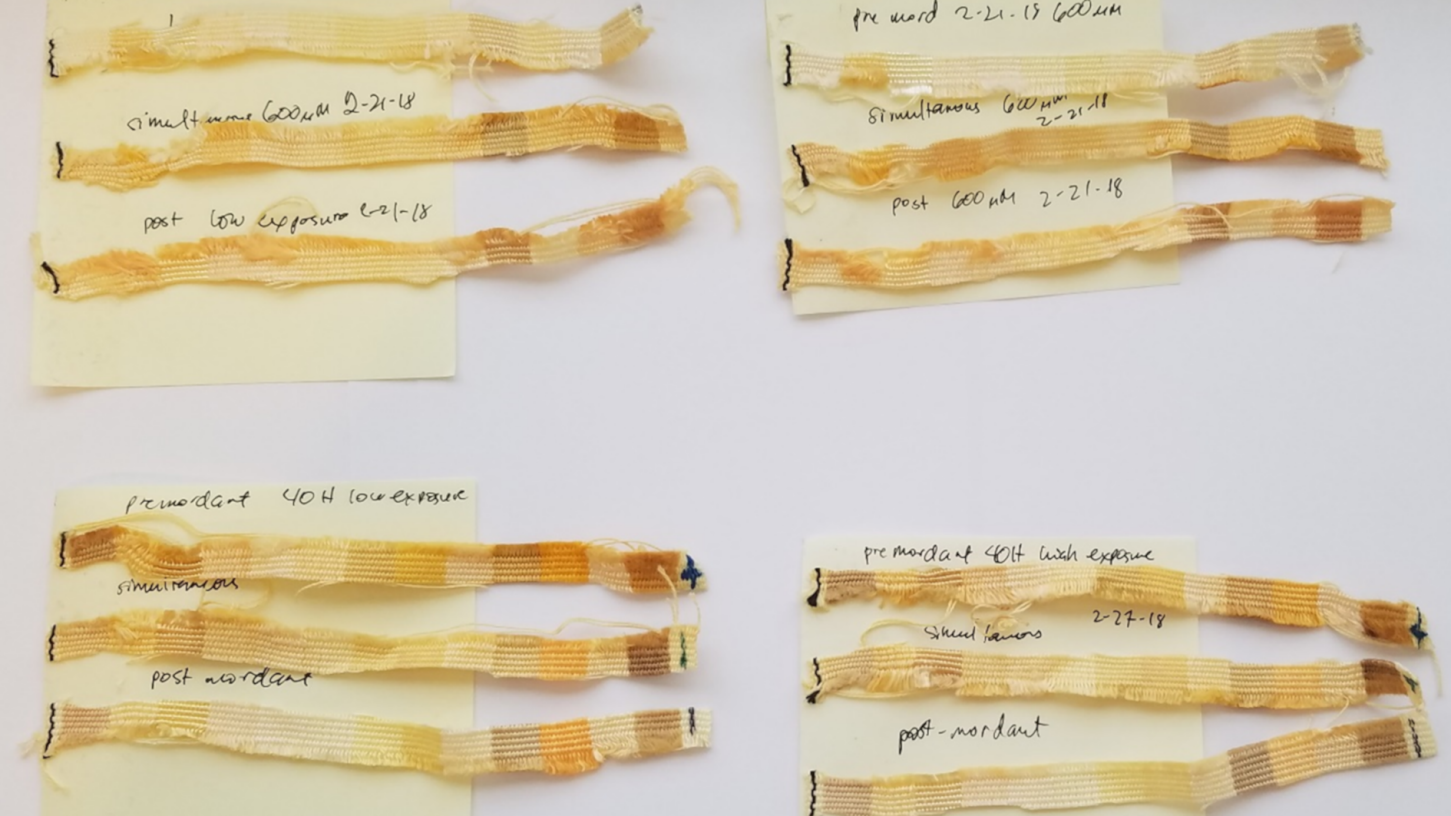
Fabric dying and photobleaching. The top two sets show dyed fabric strips with premordant, simultaneous, and post-mordant dying (top to bottom) using aurone **10** after dyeing (left) and after one week of sun exposure (right). The bottom two are similarly dyed fabric strips with premordant, simultaneous, and post-mordant dying (top to bottom) using aurone **15** after dyeing (left) and after one week of sun exposure (right). The fabric order on the strip is acetate, rayon, sef, arnel, cotton, creslan, dacron 54, dacron 64, nylon 6.6, orlon 75, silk, polypropylene, viscose rayon, and wool from left to right.

As the initial inspiration for this work was the potential of aurones as textile dyes, two of the more interesting compounds, aurones **15** and **10** were used in a very preliminary attempt at fabric dyeing. Three processes were compared: pre-, simultaneous, and post-mordanting. As can be seen in Figure 4, dyeing did occur in all cases. With aurone **10**, simultaneous mordanting qualitatively appeared to be better, while with aurone **15** pre-mordanting was superior. Aurone **15** afforded more vibrant colors in general and also adhered to a wider range of fabrics. While several were natural fibers (silk and wool in particular), synthetics including polypropylene were also substrates. This feature is quite remarkable and has the potential to make aurone dyes useful in dyeing polypropylene, a substrate that is generally only dyed with any efficiency using an extrusion method. Of equal interest was that a highly qualitative photobleaching study indicated a significant degree of stability of these aurone-based dyes, with simultaneous or pre-mordanting offering better stability. Clearly further studies of the generality of this observation as well as its longer term retention and photostability are required.

## Conclusion

In conclusion, the benzofuranone portion of the aurone skeleton does have a definite impact on both the UV–vis spectral and cytotoxicity properties of aurones. Interestingly, the position of the substituent was often more important than the exact substituent (at least for the examples studied), with halogen or hydroxy at the 4-position are red-shifted by roughly 10 nm compared to the unsubstituted compound and any halogen or methyl at the 5-position likewise displays a similar shift. Only hydroxylation at the 6-position displays a significant blue shift by roughly 40 nm. With respect to toxicity, halogens were the least toxic substituents, displaying the lowest cytotoxicity when at the 6- or 7-positions. Hydroxylation or substitution at the 4-position invariably lead to higher cytotoxicity, though often times no worse than the unsubstituted benzofuranone system. Finally, preliminary dyeing studies showed the expected absorption on natural fibers, but also an unexpected affinity for polypropylene. Future studies of these and other aurones are underway and the results will be reported in due course.

## Experimental

Full details of the synthesis and photochemical studies can be found in Supporting Information File 1 as well as copies of the spectra for all new compounds. Raw data for the toxicity and absorption studies can be found in Supporting Information File 2.

## Supporting Information

File 1 Experimental methods and spectra for all new compounds.

File 2 Absorption data and individual plots for all compounds.
