# Supplementary material for: A golden opportunity: benzofuranone modifications of aurones and their influence on optical properties, toxicity, and potential as dyes
Source: Beilstein J Org Chem. 2019 Jul 25;15:1781–5. doi: 10.3762/bjoc.15.171 (PMC6664396; doi:10.3762/bjoc.15.171)

BEILSTEIN JOURNAL OF ORGANIC CHEMISTRY

Supporting Information

for

A golden opportunity: benzofuranone modifications of aurones and their influence on optical properties, toxicity, and potential as dyes

Joza Schmitt and Scott T. Handy

Beilstein J. Org. Chem. **2019,** *15,* 1781–1785. doi:10.3762/bjoc.15.171

Experimental methods and spectra for all new compounds

License and Terms: This is a supporting information file under the terms of the Creative Commons Attribution License (http://creativecommons.org/licenses/by/4.0). Please note that the reuse, redistribution and reproduction in particular requires that the authors and source are credited.
The license is subject to the *Beilstein Journal of Organic Chemistry* terms and conditions: (https://www.beilstein-journals.org/bjoc)

Table of contents

Synthetic methods	S2–S3
Characterization for aurones	S3–S10
UV–vis procedure	S10
Cytotoxicity procedure	S10–S11
Dyeing procedure	S11–S12
References	S12
Copies of NMR and MS spectra	S13–S63

Instrumentation

The structures of the newly synthesized aurones were determined by NMR (JEOL ECA-500 and ECX-300) and GC–MS (Shimadzu QP2010S). UV-Vis analyses were performed on an HP 8452A Diode Array spectrophotometer. Spectrometry results from MTT assay were analyzed using Molecular Devices SpectraMax M5 Multi-Mode Microplate Reader.

General procedure for the condensation of non-hydroxy-substituted benzofuranones

In a round bottom flask, 1 mmol of the appropriate benzofuranone was mixed with 2 mmol of *p*-tolualdehyde in approximately 3.5 g of neutral alumina and 5 mL of dichloromethane. The reaction was stirred at room temperature for 24 hours. The alumina was filtered out and the mixture concentrated *in vacuo*. Two equivalents of isonicotinic acid hydrazide was added to the concentrated mixture in 5 mL/mmol of both dichloromethane and dimethylformamide and left to stir at room temperature for another 24 hours to remove any excess aldehyde. In some cases, further purification methods were used and/or the ratio of the hydrazide scavenger and the solvents were changed to increase the scavenger's effectiveness after air exposure. Exceptions are noted below. The aurone product was extracted using a mixture of ethyl acetate and 1M HCl except where noted. The organic layer was concentrated and subjected to NMR and GC/MS analysis for purity.

General synthetic procedure for hydroxyl-substituted benzofuranones

In a round bottom flask, 1 mmol of the appropriate benzofuranone was mixed with 2 mmol of *p*-tolualdehyde in 1.5 g/mmol of 1:1 (w:w) KOH/H_2O and 5 mL/mmol methanol. The mixture was left to stir for 6 hours at room temperature. The mixture was triturated with 1 M HCl until acidic and precipitates formed. The crystals were filtered out and washed with 100% hexane followed by 25% EtOAc in hexane. Finally, 50% EtOAc in hexane was used as a final wash. The product was analyzed via NMR and GC/MS.

2-(4-Methylbenzylidene)-1-(benzofuran-3-one) [1]: used 4 equivalents of the hydrazide scavenger and 5 mL/0.5 mmol of DCM and DMF to produce 0.15 g (62%) of the desired product as a pale yellow solid (mp = 97-99 °C). ^{1}H NMR (500 MHz, $CDCl_3$): δ= 2.29 (s, 3H), 6.76 (s, 1H), 7.08 (t, J=7.4 Hz, 1H), 7.13 (d, J=8.4 Hz, 2H), 7.19 (d, J=8.6 Hz, 1H), 7.51 (t, J=8.6 Hz, 1H), 7.68 (d, J=8.4 Hz, 3H); ^{13}C NMR (125 MHz, $CDCl_3$): δ= 21.67, 112.93, 113.27, 121.74, 123.34, 124.48, 129.53, 129.70 (2C), 131.62 (2C), 136.68, 140.43, 146.47, 165.91, 184.51; GC RT: (24.81 minutes); MS (200 °C, 70 eV): 236 (M^+, 95.1), 235 (96.3), 221 (100.0), 92 (46.7), 76 (58.6).

2-(4-Methylbenzylidene)-1-(5'-fluorobenzofuran-3-one) [2]: purified via column chromatography using silica gel and 10:90 EtOAc/hexane as eluent followed by extraction in 1M HCl and EtOAc to give 0.15 g (59%) of the product as a pale yellow solid (mp = 122-124 °C). ^{1}H NMR (500 MHz, $CDCl_3$): δ= 2.41 (s, 3H), 6.91 (s, 1H), 7.26 (d, *J*=8.2 Hz, 2H), 7.30 (dd, *J*=8.6, 3.4 Hz, 1H), 7.36 (td, *J*=8.6, 2.3 Hz, 1H), 7.45 (dd, *J*=6.3, 2.8 Hz, 1H), 7.80 (d, *J*=5.8 Hz, 2H); ^{13}C NMR (125 MHz,

$CDCl_3$): δ= 21.69, 109.91 (d, J=95 Hz), 114.07 (d, J=33 Hz), 114.21, 122.39 (d, J=33 Hz), 123.93 (d, J=104 Hz), 129.47 (d, J=248 Hz), 129.77 (2C), 131.74 (2C), 140.85, 147.03, 157.78, 161.90, 183.86; GC RT: (24.00 minutes); MS (200 °C, 70 eV): 254 (M^+, 47.9), 253 (49.0), 239 (100.0), 126 (53.9), 115 (41.7).

2-(4-Methylbenzylidene)-1-(6'-fluorobenzofuran-3-one) [3]: a new batch of the hydrazide scavenger was reintroduced after column chromatography giving 0.1 g (39%) of the product as a pale yellow solid (mp = 172-174 °C). ^{1}H NMR (500 MHz, $CDCl_3$): δ= 2.40 (s, 3H), 6.87 (s, 1H), 6.90 (td, *J*=14.9, 3.4 Hz, 1H), 7.0 (dd, *J*=14.3, 3.4 Hz, 1H), 7.25 (d, *J*=13.2 Hz, 2H), 7.77 (d, *J*=13.2 Hz, 2H), 7.80 (d, *J*=13.8 Hz, 1H); ^{13}C NMR (125 MHz, $CDCl_3$): δ= 21.74, 100.77(d, J=104 Hz), 111.86 (d, J=95 Hz), 113.75, 118.47, 126.45 (d, J=48 Hz), 129.55 (d, J=286 Hz), 129.83 (2C), 131.70 (2C), 140.83, 146.89, 167.14 (d, J=33 Hz), 169.25, 182.97; GC RT: (23.88 minutes); MS (200 °C, 70 eV): 254 (M^+, 56.3), 253 (65.4), 239 (100.0), 126 (61.2), 115 (50.6).

2-(4-Methylbenzylidene)-1-(4'-chlorobenzofuran-3-one) [4]: required 4 equivalents of the hydrazide scavenger and 5 mL/0.5 mmol of DCM and DMF to yield 0.2 g (74%) of the desired product as a pale yellow solid (mp = 172-173 °C). ^{1}H NMR (500 MHz, $CDCl_3$): δ= 2.33 (s, 3H), 6.77 (s, 1H), 7.04 (d, *J*=8.0 Hz, 1H), 7.12 (d, *J*=8.0 Hz, 1H), 7.15 (d, *J*=8.0 Hz, 2H), 7.43 (t, *J*=8.0 Hz, 1H), 7.67 (d, *J*=8.1 Hz, 2H); ^{13}C NMR (125 MHz, $CDCl_3$): δ= 21.75, 111.39, 113.95, 119.03, 124.56, 129.24, 129.79 (2C), 131.69 (2C), 132.27, 136.76, 140.83,

146.11, 166.43, 181.77; GC RT: (26.50 minutes); MS (200 °C, 70 eV): 272 (M+2, 12.2), 270 (M^+, 34.2), 269 (40.3), 257 (31.1), 255 (100.0), 134 (58.7), 115 (53.5).

2-(4-Methylbenzylidene)-1-(5'-chlorobenzofuran-3-one) [5]: another 2 equivalents of the hydrazide scavenger were reintroduced in 5 mL/mmol DCM and DMF to yield 0.2 g (72%) of the desired product as a pale yellow solid (mp = 143-146 °C). ^{1}H NMR (500 MHz, $CDCl_3$): δ= 2.39 (s, 3H), 6.86 (s, 1H), 7.23 (d, *J*=6.8 Hz, 2H), 7.55 (dd, *J*=8.6, 2.3 Hz, 1H), 7.71 (d, *J*=2.3 Hz, 1H), 7.75 (d, *J*=8.0 Hz, 2H); ^{13}C NMR (125 MHz, $CDCl_3$): δ= 21.76, 114.25, 114.41, 122.91, 124.09, 129.06, 129.17, 129.79 (2C), 131.78 (2C), 136.40, 140.98, 146.57, 164.05, 183.21; GC RT: (25.93 minutes); MS (200 °C, 70 eV): 272 (M+2, 14.2), 270 (M^+, 43.9), 269 (44.6), 257 (33.8), 255 (100.0), 134 (59.6), 115 (76.4).

2-(4-Methylbenzylidene)-1-(6'-chlorobenzofuran-3-one) [6]: another 2 equivalents of the hydrazide scavenger were reintroduced in 5 mL/mmol DCM and DMF to yield 0.19 g (70%) of the product as a pale yellow solid (mp = 165-166 °C). ^{1}H NMR (500 MHz, $CDCl_3$): δ= 2.37 (s, 3H), 6.83 (s, 1H), 7.13 (dd, *J*=8.1, 1.7 Hz ,1H), 7.21 (d, *J*=8.0 Hz, 2H), 7.29 (s, 1H), 7.66 (d, *J*=8.2 Hz, 1H), 7.72 (d, *J*=8.6 Hz, 2H); ^{13}C NMR (125 MHz, $CDCl_3$): δ= 21.76, 113.65, 114.23, 120.48, 124.30, 125.38, 129.24, 129.84 (2C), 131.75 (2C), 140.94, 142.72, 146.59, 166.06, 183.21; GC RT: (25.81 minutes); MS (200 °C, 70 eV): 272 (M+2, 15.3), 270 (M^+, 46.0), 269 (46.3), 257 (31.3), 255 (100.0), 134 (63.5), 115 (79.4).

2-(4-Methylbenzylidene)-1-(7'-chlorobenzofuran-3-one) [7]: another 2 equivalents of the hydrazide scavenger were reintroduced in 5 mL/mmol DCM and DMF to yield 0.14 g (50%) of the product as a pale yellow solid (mp = 172-174 °C). ^{1}H NMR (500 MHz, $CDCl_3$): δ= 2.37 (s, 3H), 6.90 (s, 1H), 7.11 (t, *J*=7.7 Hz, 1H), 7.23 (d, *J*=8.0 Hz, 2H), 7.59 (d, *J*=7.4 Hz, 1H), 7.65 (d, *J*=7.4 Hz, 1H), 7.80 (d, *J*=8.1 Hz, 2H); ^{13}C NMR (125 MHz, $CDCl_3$): δ= 21.80, 115.01, 118.62, 122.86, 123.65, 124.14, 129.17, 129.93 (2C), 132.02 (2C), 136.34, 141.16, 146.26, 161.53, 183.79; GC RT: (25.54 minutes); MS (200 °C, 70 eV): 272 (M+2, 16.6), 270 (M^+, 47.8), 269 (45.0), 257 (33.5), 255 (100.0), 134 (63.7), 115 (81.1).

2-(4-Methylbenzylidene)-1-(5'-bromobenzofuran-3-one) [8]: another 2 equivalents of the hydrazide scavenger were reintroduced in 5 mL/mmol DCM and DMF to yield 0.15 g (42%) of the desired product as a pale yellow solid (mp = 154-155 °C). ^{1}H NMR (500 MHz, $CDCl_3$): δ= 2.41 (s, 3H), 6.90 (s, 1H), 7.23 (d, *J*=8.6 Hz, 1H), 7.25 (d, *J*=8.2 Hz, 2H), 7.71 (dd, *J*=8.6, 1.7 Hz, 1H), 7.78 (d, *J*=8.6 Hz, 2H), 7.90 (d, *J*=2.3 Hz, 1H); ^{13}C NMR (125 MHz, $CDCl_3$): δ= 21.63, 114.42, 114.57, 116.14, 123.36, 127.14, 129.04, 129.69 (2C), 131.66 (2C), 139.07, 140.91, 146.29, 164.41, 183.03; GC RT: (26.89 minutes); MS (200 °C, 70 eV): 316 ($[M+2-1]^+$, 31.8), 315 (M^+, 34.5), 314 ($[M-1]^+$, 32.4), 301 (69.9), 299 (74.2), 115 (100.0), 91 (14.8).

2-(4-Methylbenzylidene)-1-(6'-bromobenzofuran-3-one) [9]: scavenger was reintroduced three times to produce 0.26 g (83%) of the desired product as a

pale yellow solid (mp = 157-158 °C). ^{1}H NMR (500 MHz, $CDCl_3$): δ= 2.37 (s, 3H), 6.84 (s, 1H), 7.20 (d, *J*=8.1 Hz, 2H), 7.29 (d, *J*=8.0 Hz, 1H), 7.47 (s, 1H), 7.59 (d, *J*=8.0 Hz, 1H), 7.72 (d, *J*=8.2 Hz, 2H); ^{13}C NMR (125 MHz, $CDCl_3$): δ= 21.79, 114.30, 116.64, 120.86, 125.47, 127.11, 129.25, 129.85 (2C), 131.15, 131.77 (2C), 140.96, 146.45, 165.96, 183.36; GC RT: (26.79 minutes); MS (200 °C, 70 eV): 316 ($[M+2-1]^+$, 29.3), 315 (M^+, 35.2), 314 ($[M-1]^+$, 30.2), 301 (53.6), 299 (58.9), 115 (100.0), 91 (16.8).

2-(4-Methylbenzylidene)-1-(7'-bromobenzofuran-3-one) [10]: scavenger was reintroduced two times to yield 0.18 g (53%) of the product as a pale yellow solid (mp = 187-192 °C). ^{1}H NMR (500 MHz, $CDCl_3$): δ= 2.40 (s, 3H), 6.93 (s, 1H), 7.08 (t, *J*=7.7 Hz, 1H), 7.26 (d, *J*=8.0 Hz, 2H), 7.72 (d, *J*=8.2 Hz, 1H), 7.77 (d, *J*=8.1 Hz, 1H), 7.84 (d, *J*=8.0 Hz, 2H); ^{13}C NMR (125 MHz, $CDCl_3$): δ= 21.66, 106.08, 114.85, 123.39, 124.43, 129.06 129.81 (2C), 131.91 (2C), 139.11, 141.02, 146.15, 162.75, 183.85; GC RT: (26.41 minutes); MS (200 °C, 70 eV): 316 ($[M+2-1]^+$, 37.5), 315 (M^+, 38.2),314 ($[M-1]^+$, 38.6), 301 (72.2), 299 (75.4), 115 (79.6), 91 (25.2).

2-(4-Methylbenzylidene)-1-(5'-methylbenzofuran-3-one) [11]: scavenger was reintroduced one more time to produce 0.14 g (56%) of the desired product as a pale yellow solid (mp = 117-119 °C). ^{1}H NMR (300 MHz, $CDCl_3$): δ= 2.37 (s, 3H), 2.38 (s, 3H), 6.83 (s, 1H), 7.17 (d, *J*=8.6 Hz, 1H), 7.22 (d, *J*=7.9 Hz, 2H), 7.41 (d, *J*=8.6 Hz, 1H), 7.55 (s, 1H), 7.77 (d, *J*=7.9 Hz, 2H); ^{13}C NMR (125 MHz, $CDCl_3$):

δ= 20.84, 21.71, 112.54, 113.04, 121.67, 124.20, 129.67, 129.73 (2C), 131.60 (2C), 133.09, 137.91, 140.39, 146.97, 164.51, 184.83; GC RT: (25.50 minutes); MS (200 °C, 70 eV): 250 (M^+, 69.2), 249 (74.0), 235 (100), 134 (54.5), 124 (77.5), 89 (60.2).

2-(4-Methylbenzylidene)-1-(5',6'-dimethylbenzofuran-3-one) [12]: another 2 equivalents of the hydrazide scavenger were reintroduced in 5 mL/mmol DCM and DMF to yield 0.11 g (40%) of the product as a pale yellow solid (mp = 181-183 °C). ^{1}H NMR (500 MHz, $CDCl_3$): δ= 2.22 (s, 3H), 2.31 (s, 3H), 2.36 (s, 3H), 6.76 (s, 1H), 7.03 (s, 1H), 7.20 (d, *J*=8.0 Hz, 2H), 7.45 (s, 1H), 7.73 (d, *J*=8.1 Hz, 2H); ^{13}C NMR (125 MHz, $CDCl_3$): δ= 19.28, 21.30, 21.48, 112.23, 113.18, 119.33, 124.16, 129.48 (2C), 129.59, 131.26 (2C), 132.01, 139.95, 146.93, 147.59, 164.98, 184.27; GC RT: (27.00 minutes); MS (200 °C, 70 eV): 264 (M^+, 68.2), 263 (84.2), 249 (91.45), 148 (42.3), 131 (55.9), 117 (53.7), 91 (100.0).

2-(4-Methylbenzylidene)-1-(7'-methylbenzofuran-3-one) [13]: 0.13 g (52%) yield as a pale yellow solid (mp = 107-108 °C). ^{1}H NMR (500 MHz, CDCl3): δ= 2.37 (s, 3H), 2.44 (s, 3H), 6.83 (s, 1H), 7.05 (t, *J*=7.4 Hz, 1H), 7.22 (d, *J*=8.0 Hz, 2H), 7.39 (d, *J*=6.9 Hz, 1H), 7.58 (d, *J*=8.0 Hz, 1H), 7.76 (d, *J*=8.0 Hz, 2H); ^{13}C NMR (125 MHz, CDCl3): δ= 14.38, 21.72, 113.11, 121.26, 121.90, 122.97, 123.29, 128.74 (3C- two are coincident), 131.62 (2C), 137.60, 140.42, 146.74, 164.79, 185.20; GC RT: (25.08 minutes); MS (200 °C, 70 eV): 250 (M^+, 58.9), 249 (66.0), 235 (100), 124 (61.0), 106 (70.0), 89 (59.3).

2-(4-Methylbenzylidene)-1-(6'-hydroxybenzofuran-3-one) [14]: 0.05 g (20%) yield as an off-white solid (mp > 210 °C). ^{1}H NMR (500 MHz, $(CD_3)_2CO$): δ= 2.36 (s, 3H), 6.68 (s, 1H), 6.76 (dd, *J*=8.6, 2.3 Hz, 1H), 6.82 (d, *J*=1.7 Hz, 1H), 7.28 (d, *J*=8.0 Hz, 2H), 7.60 (d, *J*=8.0 Hz, 1H), 7.83 (d, *J*=8.6 Hz, 2H); ^{13}C NMR (125 MHz, $(CD_3)_2CO$): δ= 20.67, 98.79, 110.41, 112.82, 113.96, 125.79, 129.65 (2C), 129.88, 131.22 (2C), 139.93, 147.49, 166.08, 168.39, 181.58; GC RT: (28.08 minutes); MS (200 °C, 70 eV): 252 (M^+, 58.5), 251 (54.8), 63 (76.1), 44 (100.0).

2-(4-Methylbenzylidene)-1-(4'-hydroxybenzofuran-3-one) [15]: 0.04 g (15%) yield, as a pale yellow solid (mp = 156-158 °C). ^{1}H NMR (500 MHz, $CDCl_3$): δ= 2.41 (s, 3H), 6.61 (d, J=8.1 Hz, 1H), 6.79 (d, J=8.1 Hz, 1H), 6.83 (s,1H), 7.25 (d, J=7.4 Hz, 2H), 7.49 (t, J=8.3 Hz, 1H), 7.80 (d, J=8.0 Hz, 2H), 7.83 (x, 1H, OH); ^{13}C NMR (125 MHz, $CDCl_3$): δ= 21.77, 103.71, 109.77, 109.81, 113.59, 129.32, 129.85 (2C), 131.75 (2C), 139.19, 140.89, 146.28, 156.72, 164.90, 185.75; GC RT: (25.09 minutes); MS (200 °C, 70 eV): 252 (M^+, 98.7), 251 (77.8), 237 (100.0).

2-(4-Methylbenzylidene)-1-(6'-hydroxy-4'-methylbenzofuran-3-one) [16]: 0.03 g (11%) yield as a pale yellow solid (mp >210 °C). ^{1}H NMR (500 MHz, $CDCl_3$): δ= 2.39 (s, 3H), 2.62 (s, 3H), 6.44 (s, 1H), 6.57 (s, 1H), 6.75 (s, 1H), 7.23 (d, J=8.2 Hz, 2H), 7.76 (d, J=8.6 Hz, 2H); ^{13}C NMR (500 MHz, $(CD_3)_2CO$): δ= 16.98, 20.66, 96.34, 109.58, 112.35, 113.43, 129.61 (2C), 130.01, 131.08 (2C), 139.67, 141.38, 147.65, 165.36, 168.61, 182.43; GC RT: (28.46 minutes); MS

(200 °C, 70 eV): 266 (M^+, 72.7), 265 (100.0), 251 (70.7), 132 (82.9), 77 (90.0), 51 (84.9).

2-(4-Methylbenzylidene)-1-(6’-hydroxy-7’-methylbenzofuran-3-one) [17]: 0.05 g (19%) yield as a pale yellow solid (mp >210 °C). ^{1}H NMR (500 MHz, $(CD_3)_2CO$): δ= 2.25 (s, 3H), 2.43 (s, 3H), 6.42 (d, *J*=2.3 Hz, 1H), 6.52 (s, 1H), 6.52 (d, *J*=1.7 Hz, 1H), 7.17 (d, *J*=7.4 Hz, 2H), 7.71 (d, *J*=8.1 Hz, 2H); ^{13}C NMR (125 MHz, $(CD_3)_2CO$): δ= 16.96, 20.65, 96.33, 109.55, 112.36, 113.43, 129.62 (2C), 130.02, 131.08 (2C), 139.67, 141.37, 148.57, 165.39, 168.49, 182.42; GC RT: (28.34 minutes); MS (200 °C, 70 eV): 266 (M^+, 31.9), 265 (48.9), 251 (43.6), 77 (53.6), 44 (100.0).

General procedure for UV–vis spectroscopy

Stock solutions ranging from 87 µM to 134 µM of the aurones were made by dissolving a known mass of the aurones in acetonitrile. Each stock solution was used for subsequent dilutions to achieve a final concentration ranging from 1.5 µM to 3.3 µM. Using five different known concentration points for each aurone, the molar extinction coefficient was determined by measurement of the absorbance at the λ_{max} closest to the visible range. The absorbance measurement of the highest concentration point was used for the absorbance shift study. 2-(4-methylbenzylidene)-1-(6’-hydroxy-7’-methylbenzofuran-3-one (**17**) was the exception as the exact concentration of the solution could not be determined due to its poor solubility in acetonitrile.

Hep G2 cytotoxicity assay

Hep G2 human liver cancer cells were cultured in phenol red Dulbecco's Modified Eagle Medium (DMEM) with 10% fetal bovine serum (FBS) and 1% Pen-strep (PSG) under 37°C and 5% CO_2 atmospheric conditions. The cells were washed with phosphate buffered saline (PBS) and diluted with phenol red free DMEM to a concentration between 200,000 to 250,000 cells/mL. The cells were distributed in a 96-well plate with each well containing 100 µL of the cell solution and were allowed to settle for 2 hours in the incubator. The cells were then treated with 200 µM of the aurones in DMSO and the known dyes in 10:90 (v:v) DMSO/ultrapure water in triplicates and were left in the incubator for 3 days. The vehicles were used as the negative controls (DMSO for the aurones and 10:90 DMSO:ultrapure water for the known dyes). 20 µL of 5 mg/mL MTT (3-(4,5-dimethylthiazol-2-yl)-2,5-diphenyltetrazolium bromide) was added to each well containing treated cells after the 3-day incubation period and was allowed to settle in the incubator for 3.5 more hours. Finally, the media was removed from the wells and replaced by 100 µL of DMSO to lyse the cells and dissolve the formazan crystals that were formed. The absorbance was then recorded on the SpectraMax M5 Multi-Mode Microplate Reader. The procedure was repeated two more times using different batches of cultured Hep G2 cells on different days.

Dyeing and mordanting processes [1]

Multi-fiber fabric (Kimble 43, 13 fabric strip) was washed with warm water first then air dried. Mordanting solutions with $FeSO_4$ were made fresh at 1 g/25 mL of deionized H_2O for both the pre-mordanting and post-mordanting processes. Both (**9**) (2-

(4-methylbenzylidene)-1-(7’-bromobenzofuran-3-one)) and (**14**) (2-(4-methylbenzylidene)-1-(4’-hydroxybenzofuran-3-one)) were used as the dye samples with each undergoing pre, simultaneous, and post-mordanting processes.

References

1. Praveena, R.; Deepha, V.; Sivakumar, R. Investigations on C-glycosyl flavonoids from Rhynchosia capitata, its antimicrobial and dyeing properties. *Fibers Polym.* **2014***, 15*, 525-533.

2-(4-methylbenzylidene)-1-(5’-fluorobenzofuran-3-one) [**2**]

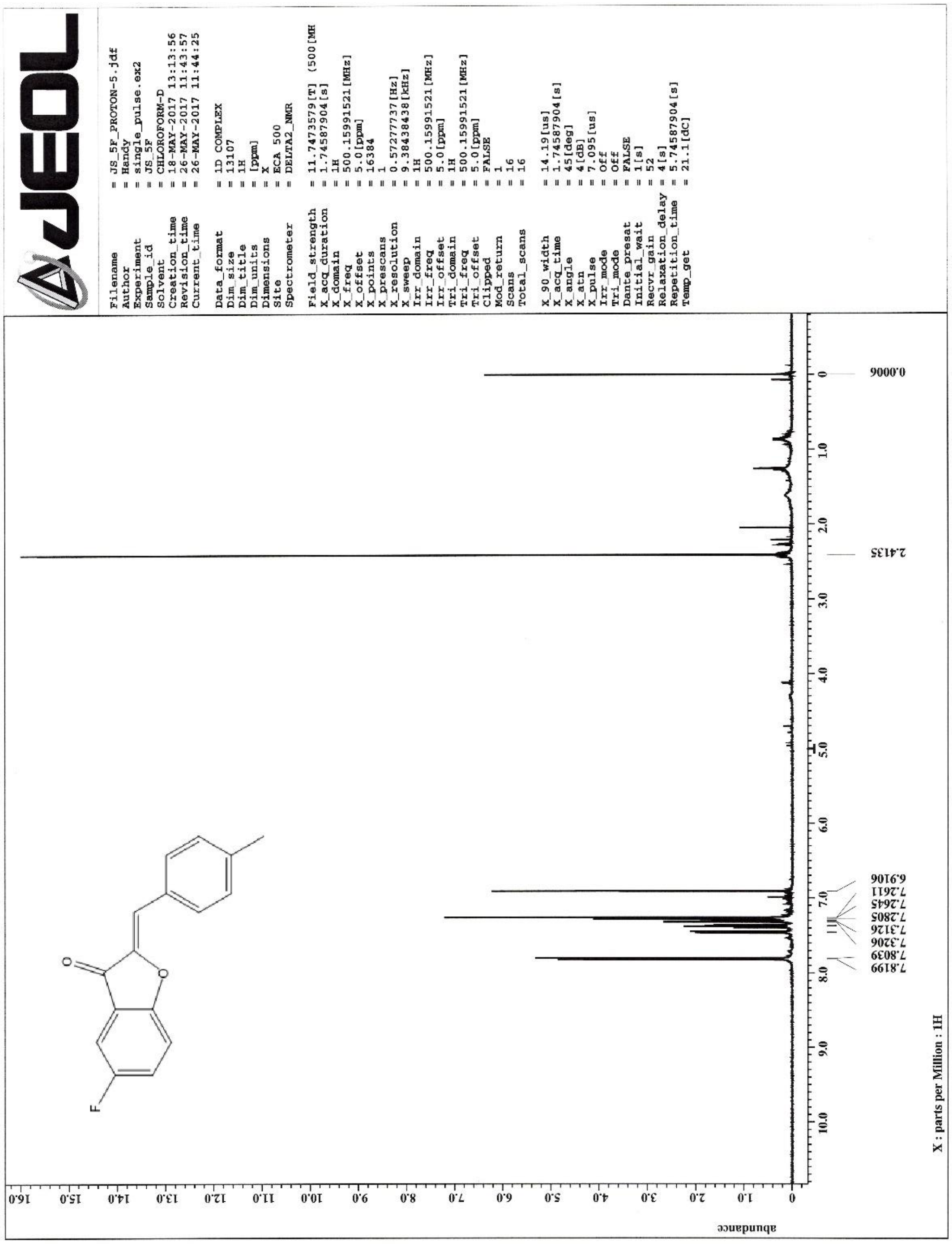

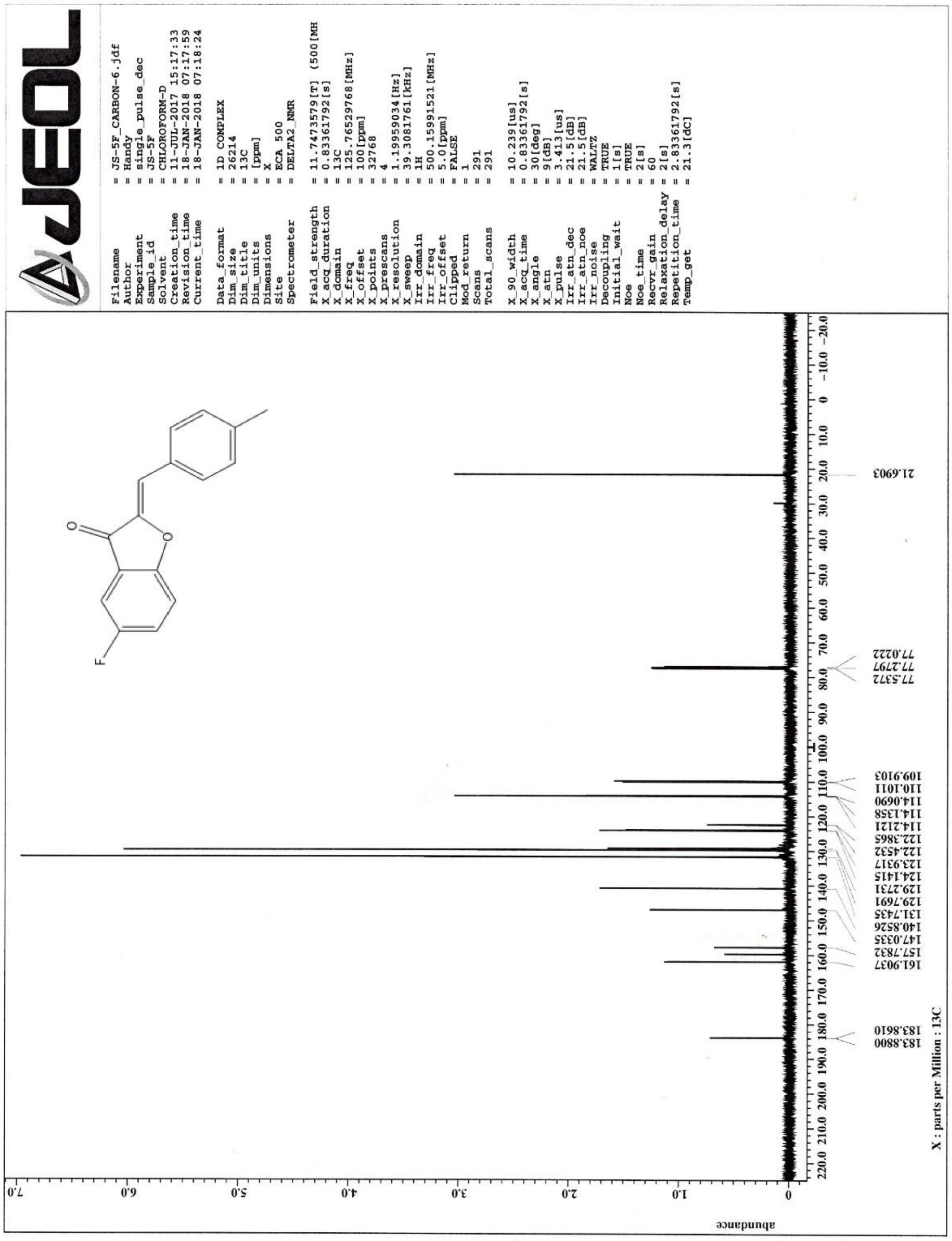
JEOL
Filename = JS-5F_CARBON-6.jdf
Author = Handy
Experiment = single_pulse_dec
Sample_id = JS-5F
Solvent = CHLOROFORM-D
Creation_time = 11-JUL-2017 15:17:33
Revision_time = 18-JAN-2018 07:17:59
Current_time = 18-JAN-2018 07:18:24
Data_format = 1D COMPLEX
Dim_size = 26214
Dim_title = 13C
Dim_units = [ppm]
Dimensions = X
Site = ECA 500
Spectrometer = DELTA2_NMR
Field_strength = 11.7473579[T] (500[MH
X_acq_duration = 0.83361792[s]
X_domain = 13C
X_freq = 125.76529768[MHz]
X_offset = 100[ppm]
X_points = 32768
X_prescans = 4
X_resolution = 1.19959034[Hz]
X_sweep = 39.3081761[kHz]
Irr_domain = 1H
Irr_freq = 500.15991521[MHz]
Irr_offset = 5.0[ppm]
Clipped = FALSE
Mod_return = 1
Scans = 291
Total_scans = 291
X_90_width = 10.239[us]
X_acq_time = 0.83361792[s]
X_angle = 30[deg]
X_atn = 9[dB]
X_pulse = 3.413[us]
Irr_atn_dec = 21.5[dB]
Irr_atn_noe = 21.5[dB]
Irr_noise = WALTZ
Decoupling = TRUE
Initial_wait = 1[s]
Noe = TRUE
Noe_time = 2[s]
Recvr_gain = 60
Relaxation_delay = 2[s]
Repetition_time = 2.83361792[s]
Temp_get = 21.3[dC]
F
O
O
183.8800
183.8610
161.9037
157.7832
147.0335
140.8526
131.7435
129.7691
129.2731
124.1415
123.9317
122.4532
122.3865
114.2121
114.1358
114.0690
110.1011
109.9103
77.5372
77.2797
77.0222
21.6903
X : parts per Million : 13C
abundance

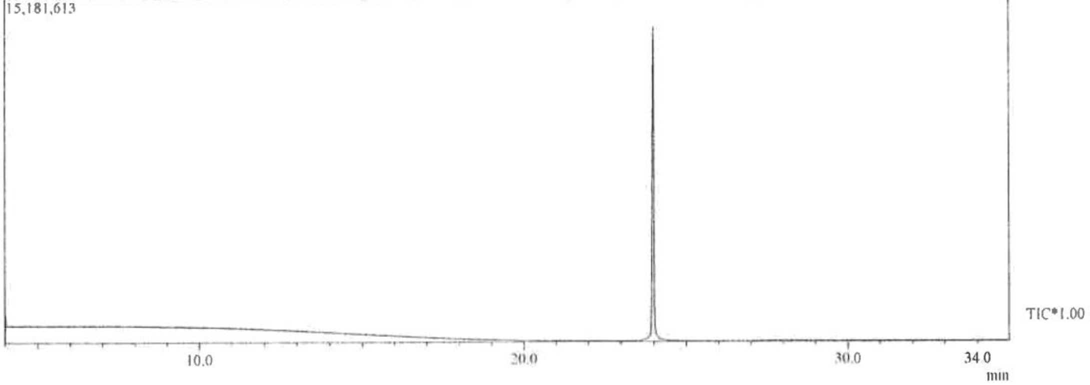

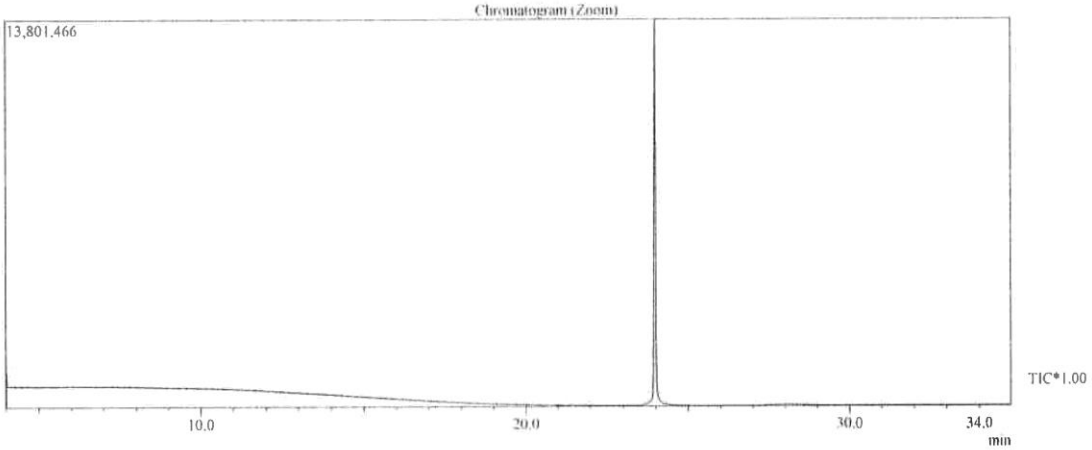

Spectrum

Line#:1 R.Time:23.975(Scan#:3996)
MassPeaks:188
RawMode:Single 23.975(3996) BasePeak:239 10(1431148)
BG Mode:None Group 1 - Event 1 Scan

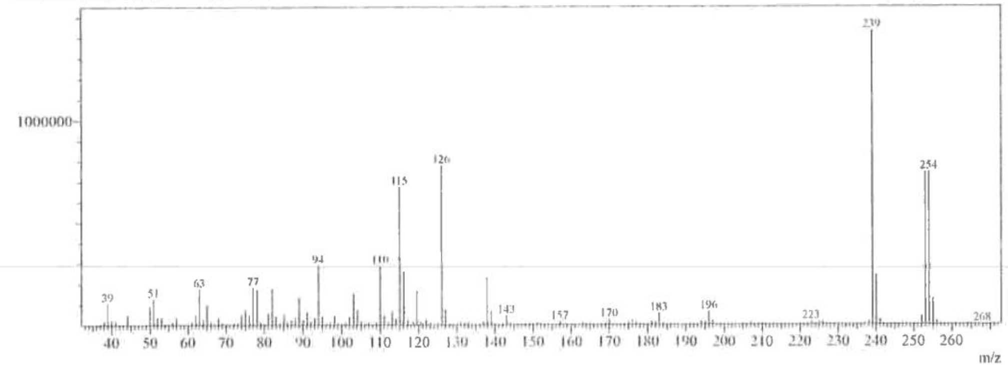

2-(4-methylbenzylidene)-1-(6’-fluorobenzofuran-3-one) [**3**]

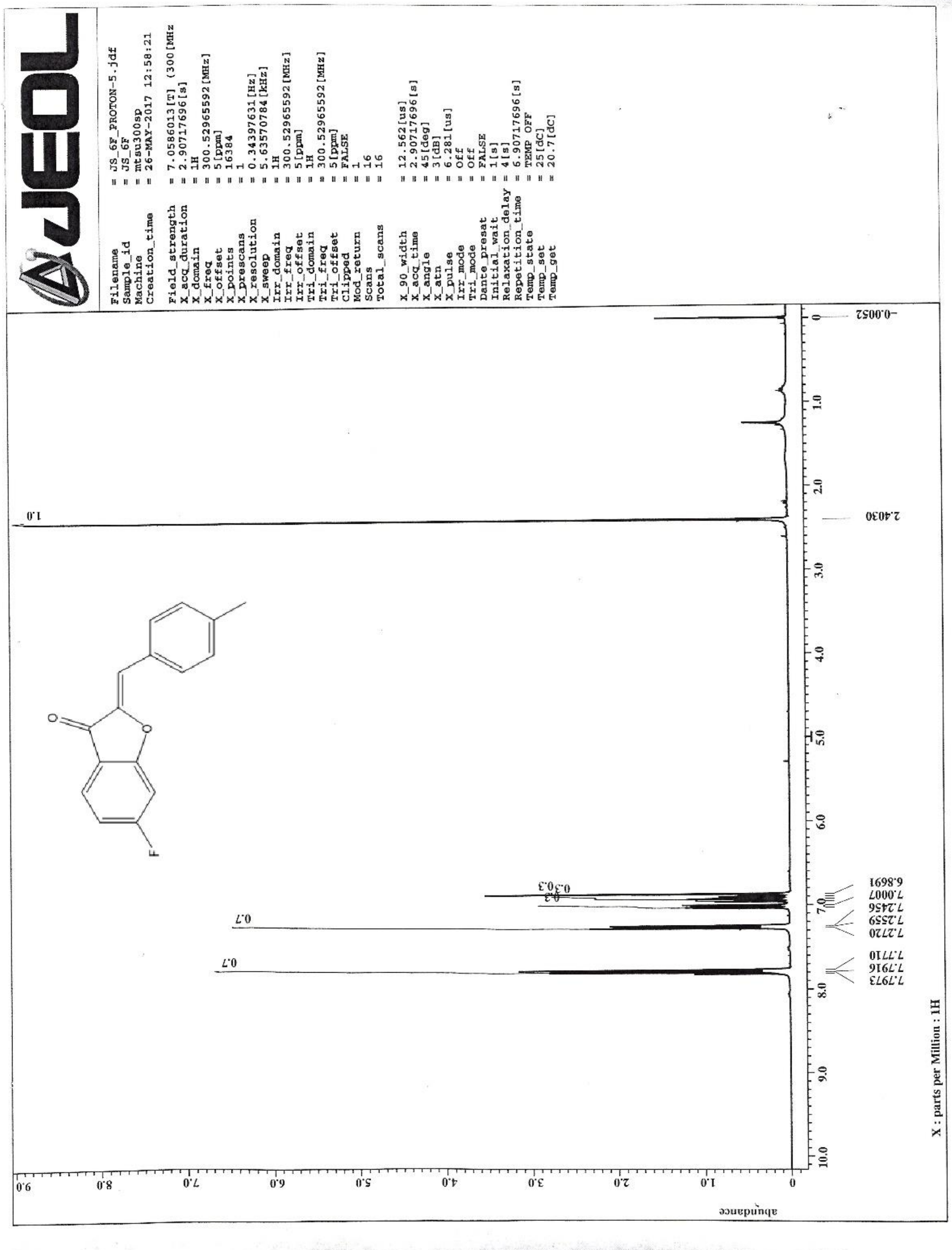

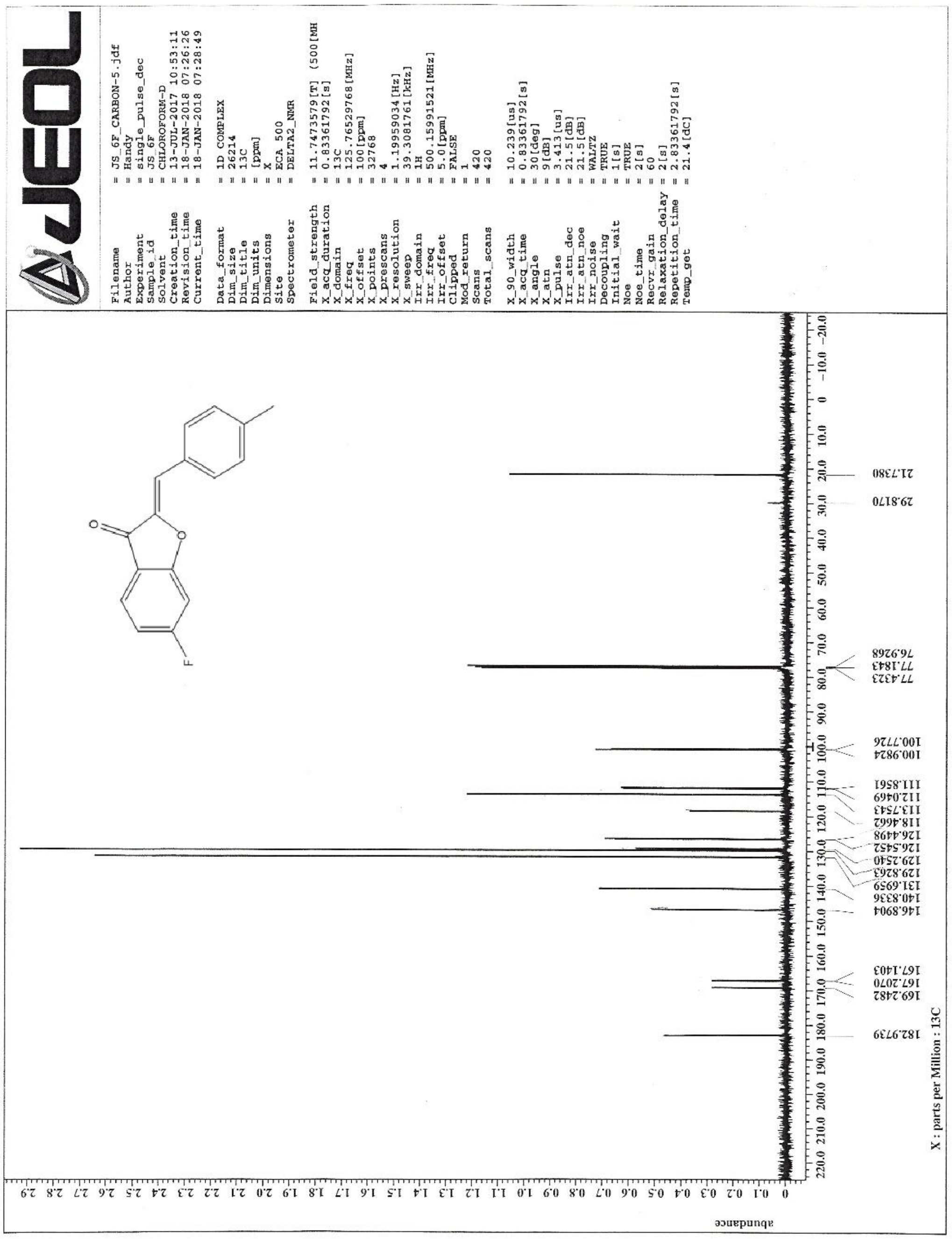
JEOL
Filename = JS_6F_CARBON-5.jdf
Author = Handy
Experiment = single_pulse_dec
Sample_id = JS_6F
Solvent = CHLOROFORM-D
Creation_time = 13-JUL-2017 10:53:11
Revision_time = 18-JAN-2018 07:26:26
Current_time = 18-JAN-2018 07:28:49
Data_format = 1D COMPLEX
Dim_size = 26214
Dim_title = 13C
Dim_units = [ppm]
Dimensions = X
Site = ECA 500
Spectrometer = DELTA2_NMR
Field_strength = 11.7473579[T] (500[MH
X_acq_duration = 0.83361792[s]
X_domain = 13C
X_freq = 125.76529768[MHz]
X_offset = 100[ppm]
X_points = 32768
X_prescans = 4
X_resolution = 1.19959034[Hz]
X_sweep = 39.3081761[kHz]
Irr_domain = 1H
Irr_freq = 500.15991521[MHz]
Irr_offset = 5.0[ppm]
Clipped = FALSE
Mod_return = 1
Scans = 420
Total_scans = 420
X_90_width = 10.239[us]
X_acq_time = 0.83361792[s]
X_angle = 30[deg]
X_atn = 9[dB]
X_pulse = 3.413[us]
Irr_atn_dec = 21.5[dB]
Irr_atn_noe = 21.5[dB]
Irr_noise = WALTZ
Decoupling = TRUE
Initial_wait = 1[s]
Noe = TRUE
Noe_time = 2[s]
Recvr_gain = 60
Relaxation_delay = 2[s]
Repetition_time = 2.83361792[s]
Temp_get = 21.4[dC]
F
O
O
21.7380
29.8170
76.9268
77.1843
77.4323
100.7726
100.9824
111.8561
112.0469
113.7543
118.4662
126.4498
126.5452
129.2540
129.8263
131.6959
140.8336
146.8904
167.1403
167.2070
169.2482
182.9739
X : parts per Million : 13C
abundance

C:\Sync\ShimadzuGCMS\Documents\Handy\Schmitt\6F.qgd

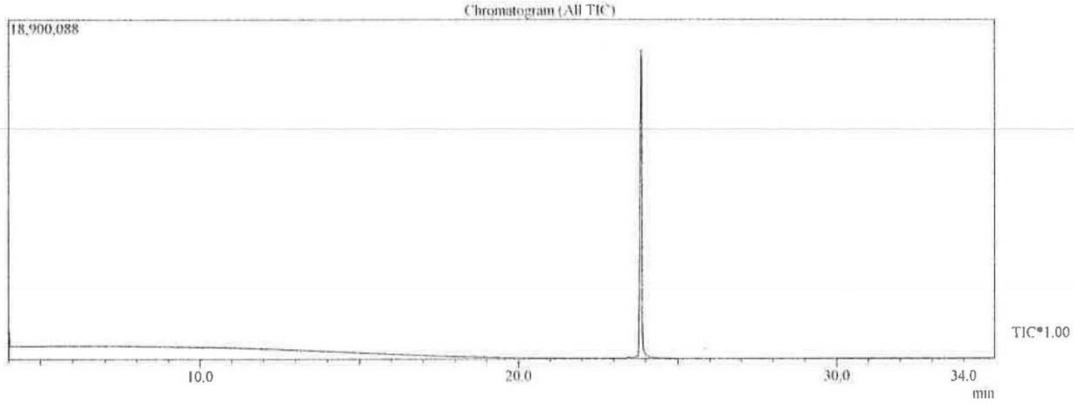

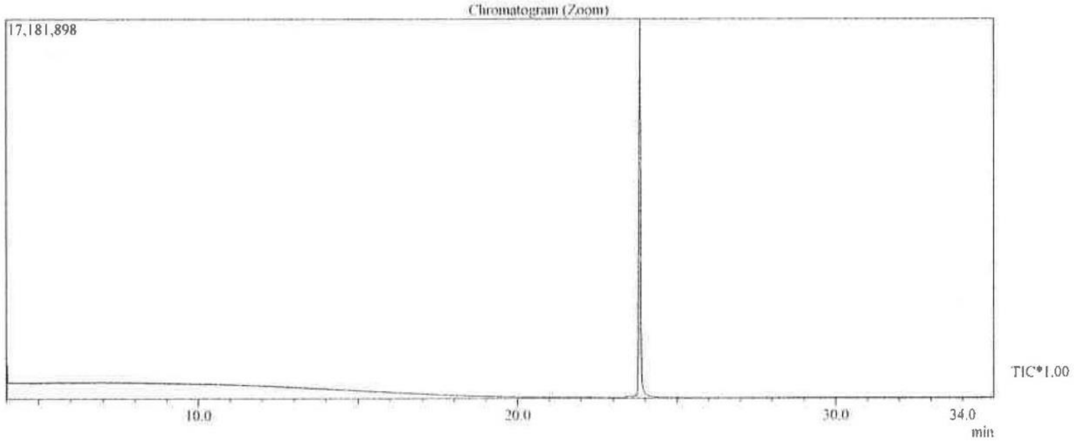

Spectrum

Line#:1 R.Time:23.865(Scan#:3974)
MassPeaks:197
RawMode:Single 23.865(3974) BasePeak:239.05(2166300)
BG Mode:None Group 1 - Event 1 Scan

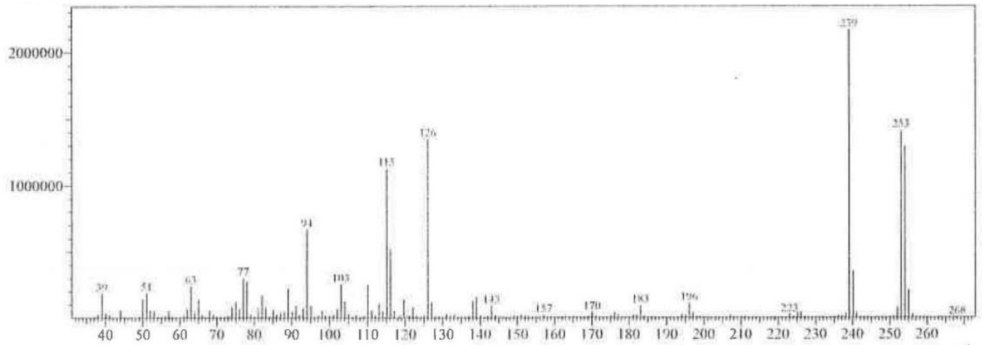

2-(4-methylbenzylidene)-1-(4’-chlorobenzofuran-3-one) [**4**]

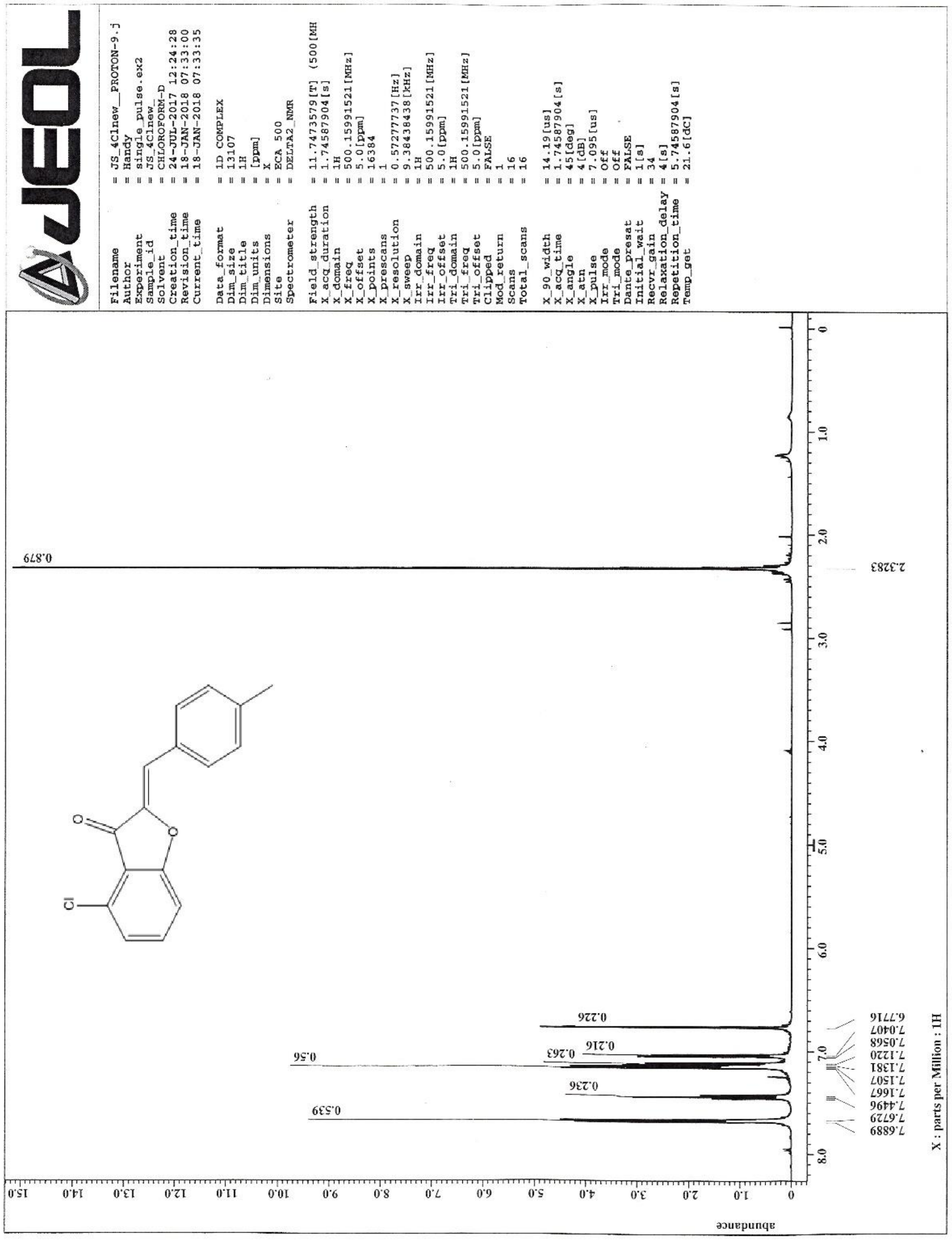

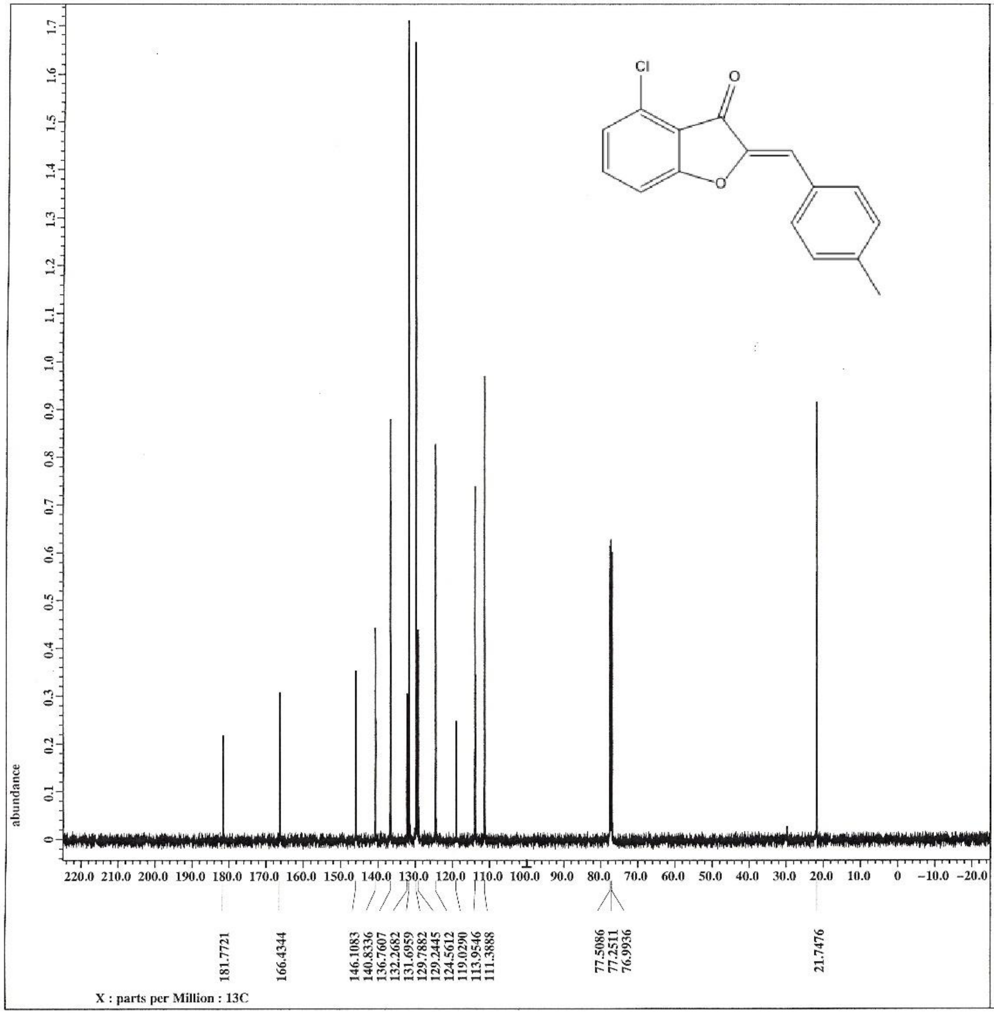

Filename	= JS_4Clnew__CARBON-6.j
Author	= Handy
Experiment	= single_pulse_dec
Sample_id	= JS_4Clnew_
Solvent	= CHLOROFORM-D
Creation_time	= 24-JUL-2017 13:13:03
Revision_time	= 18-JAN-2018 07:36:54
Current_time	= 18-JAN-2018 07:37:10
Data_format	= 1D COMPLEX
Dim_size	= 26214
Dim_title	= 13C
Dim_units	= [ppm]
Dimensions	= X
Site	= ECA 500
Spectrometer	= DELTA2_NMR
Field_strength	= 11.7473579[T] (500[MH
X_acq_duration	= 0.83361792[s]
X_domain	= 13C
X_freq	= 125.76529768[MHz]
X_offset	= 100[ppm]
X_points	= 32768
X_prescans	= 4
X_resolution	= 1.19959034[Hz]
X_sweep	= 39.3081761[kHz]
Irr_domain	= 1H
Irr_freq	= 500.15991521[MHz]
Irr_offset	= 5.0[ppm]
Clipped	= FALSE
Mod_return	= 1
Scans	= 1000
Total_scans	= 1000
X_90_width	= 10.239[us]
X_acq_time	= 0.83361792[s]
X_angle	= 30[deg]
X_atn	= 9[dB]
X_pulse	= 3.413[us]
Irr_atn_dec	= 21.5[dB]
Irr_atn_noe	= 21.5[dB]
Irr_noise	= WALTZ
Decoupling	= TRUE
Initial_wait	= 1[s]
Noe	= TRUE
Noe_time	= 2[s]
Recvr_gain	= 60
Relaxation_delay	= 2[s]
Repetition_time	= 2.83361792[s]
Temp_get	= 21.9[dC]

C:\Sync\ShimadzuGCMS\Documents\Handy\Schmitt\4Cl.qgd

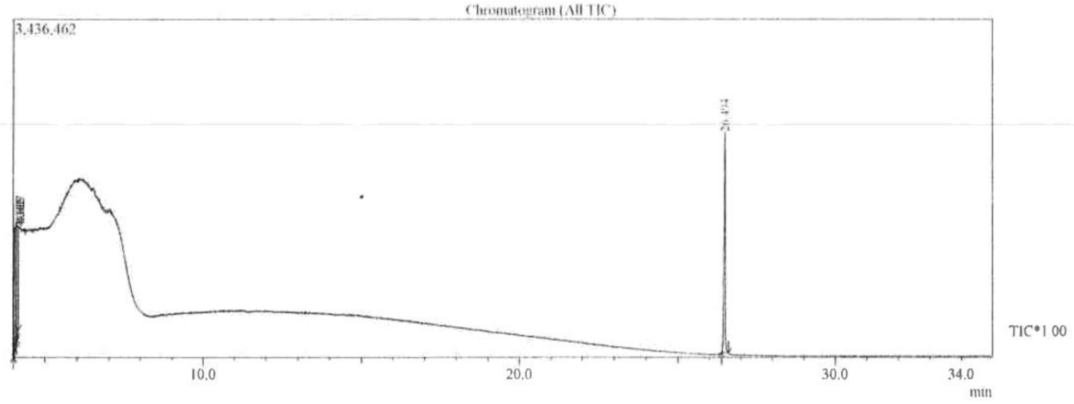

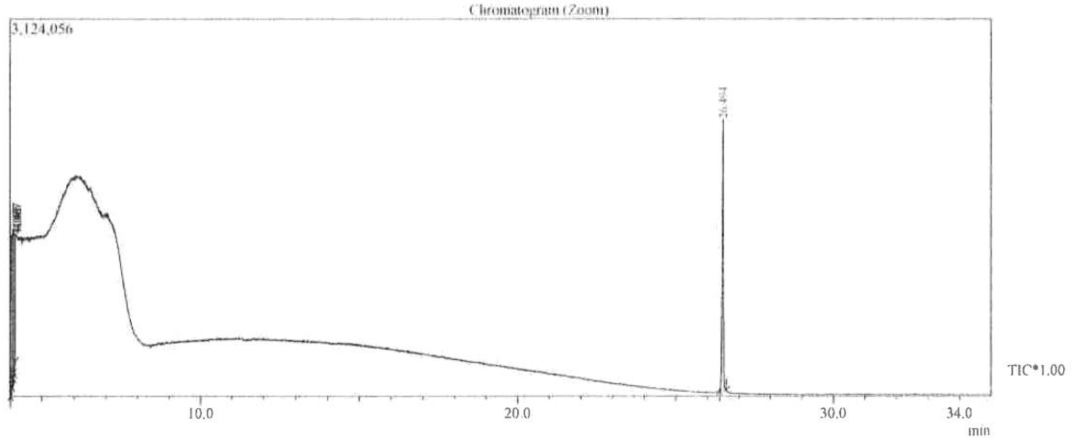

Spectrum

Line#:1 R.Time:26.495(Scan#:4500)
MassPeaks:156
RawMode:Single 26.495(4500) BasePeak:255.15(221250)
BG Mode:None Group 1 - Event 1 Scan

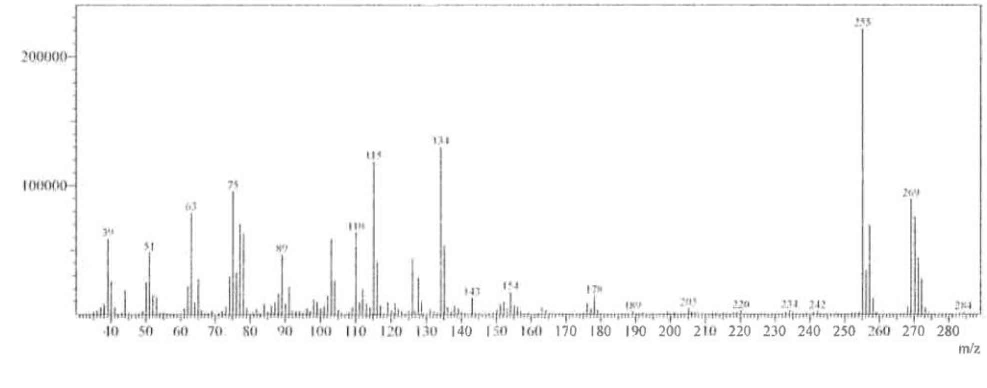

2-(4-methylbenzylidene)-1-(5′-chlorobenzofuran-3-one) [**5**]

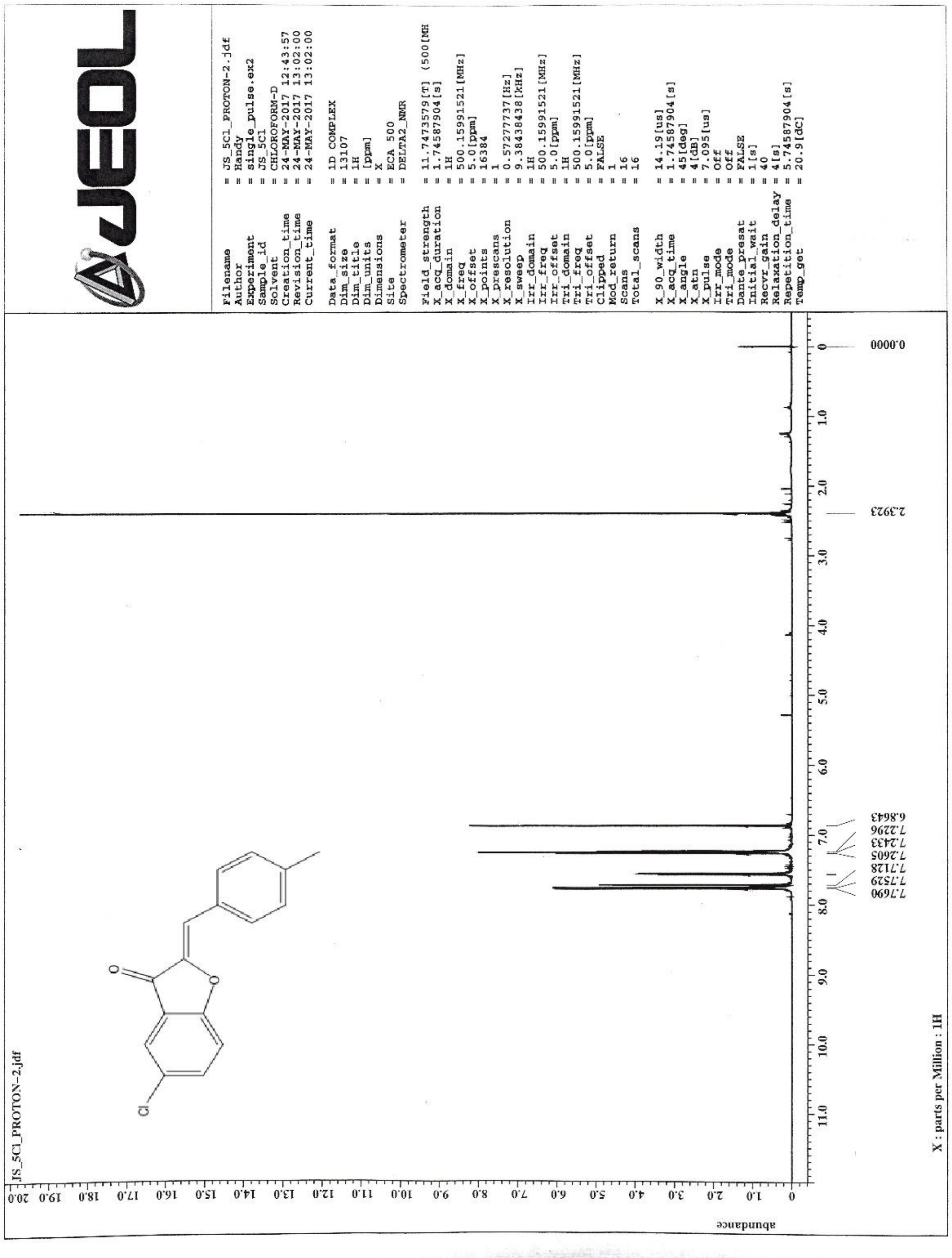

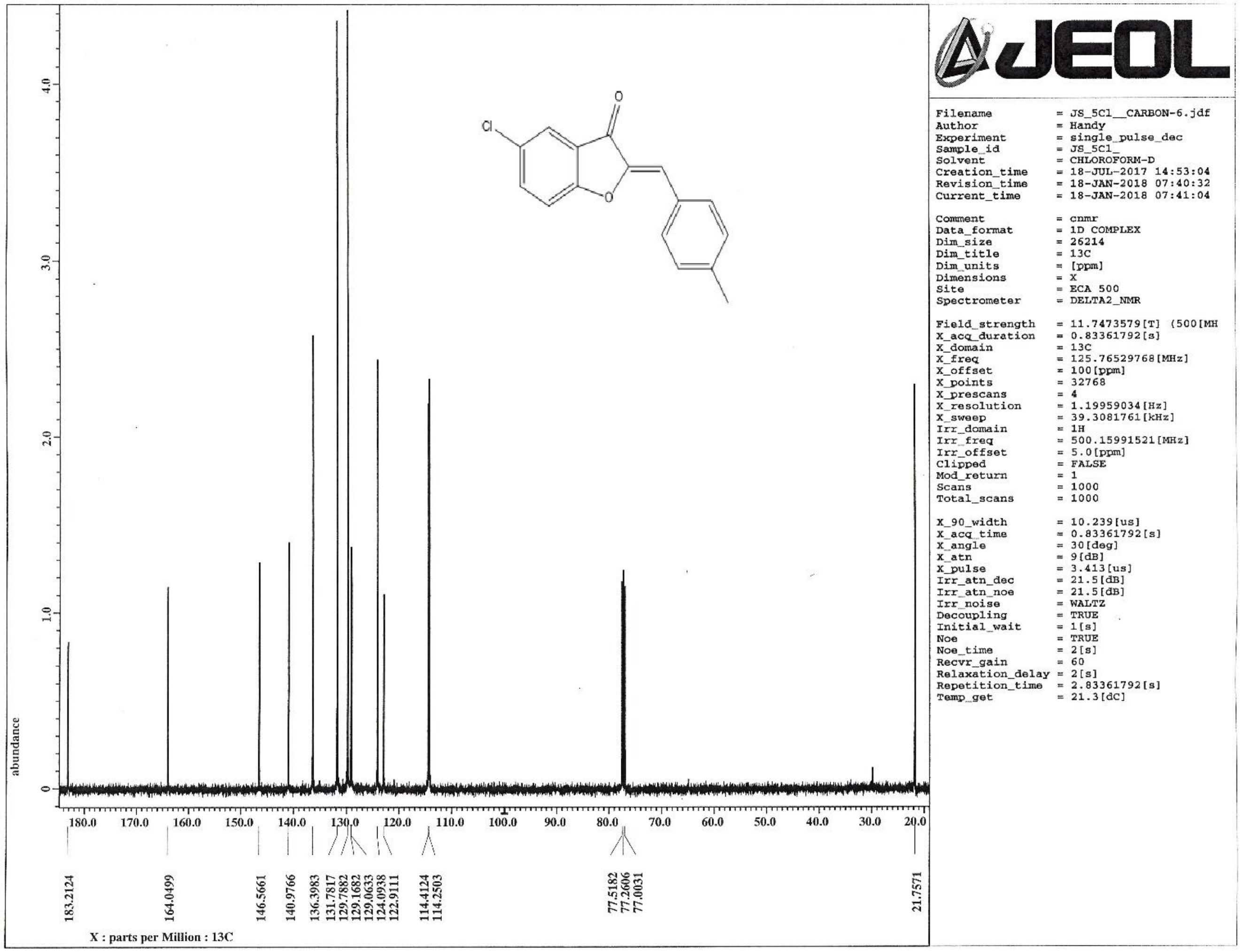
Filename = JS_5Cl__CARBON-6.jdf
Author = Handy
Experiment = single_pulse_dec
Sample_id = JS_5Cl_
Solvent = CHLOROFORM-D
Creation_time = 18-JUL-2017 14:53:04
Revision_time = 18-JAN-2018 07:40:32
Current_time = 18-JAN-2018 07:41:04
Comment = cnmr
Data_format = 1D COMPLEX
Dim_size = 26214
Dim_title = 13C
Dim_units = [ppm]
Dimensions = X
Site = ECA 500
Spectrometer = DELTA2_NMR
Field_strength = 11.7473579[T] (500[MH
X_acq_duration = 0.83361792[s]
X_domain = 13C
X_freq = 125.76529768[MHz]
X_offset = 100[ppm]
X_points = 32768
X_prescans = 4
X_resolution = 1.19959034[Hz]
X_sweep = 39.3081761[kHz]
Irr_domain = 1H
Irr_freq = 500.15991521[MHz]
Irr_offset = 5.0[ppm]
Clipped = FALSE
Mod_return = 1
Scans = 1000
Total_scans = 1000
X_90_width = 10.239[us]
X_acq_time = 0.83361792[s]
X_angle = 30[deg]
X_atn = 9[dB]
X_pulse = 3.413[us]
Irr_atn_dec = 21.5[dB]
Irr_atn_noe = 21.5[dB]
Irr_noise = WALTZ
Decoupling = TRUE
Initial_wait = 1[s]
Noe = TRUE
Noe_time = 2[s]
Recvr_gain = 60
Relaxation_delay = 2[s]
Repetition_time = 2.83361792[s]
Temp_get = 21.3[dC]
Cl
O
abundance
183.2124
164.0499
146.5661
140.9766
136.3983
131.7817
129.7882
129.1682
129.0633
124.0938
122.9111
114.4124
114.2503
77.5182
77.2606
77.0031
21.7571
X : parts per Million : 13C

C:\Sync\ShimadzuGCMS\Documents\Handy\Schmitt\5Cl.qgd

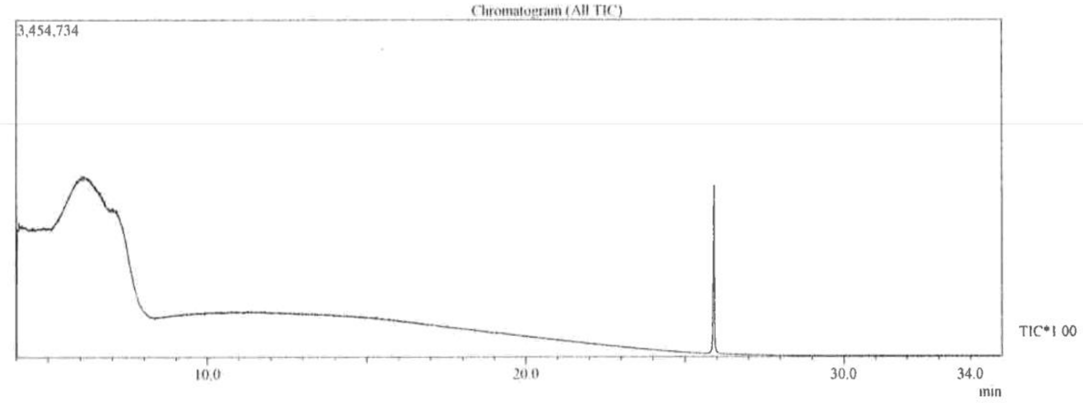

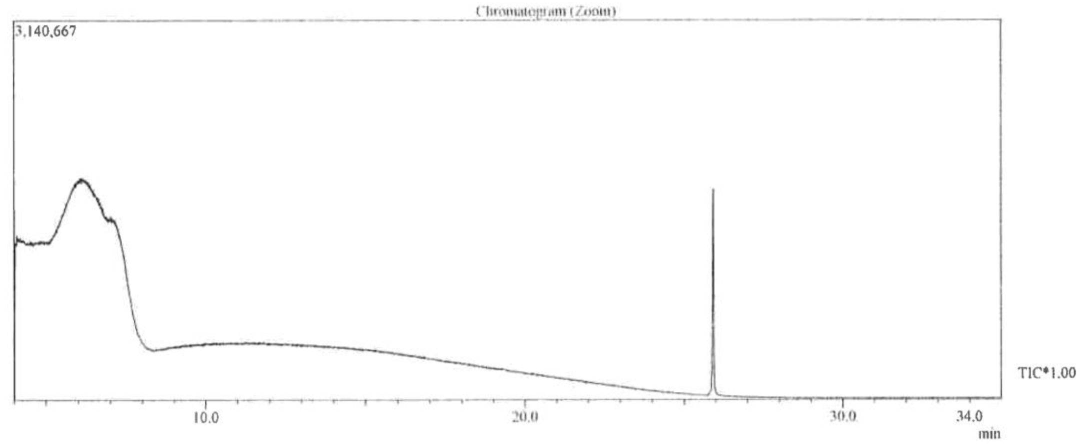

Spectrum

Line#:1 R.Time:25.910(Scan#:4383)
MassPeaks:128
RawMode:Single 25.910(4383) BasePeak:255.10(111482)
BG Mode:None Group 1 - Event 1 Scan

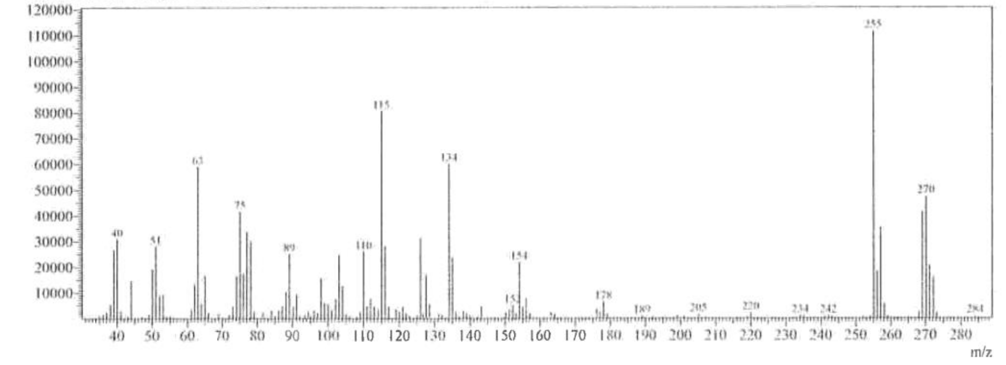

2-(4-methylbenzylidene)-1-(6’-chlorobenzofuran-3-one) [**6**]

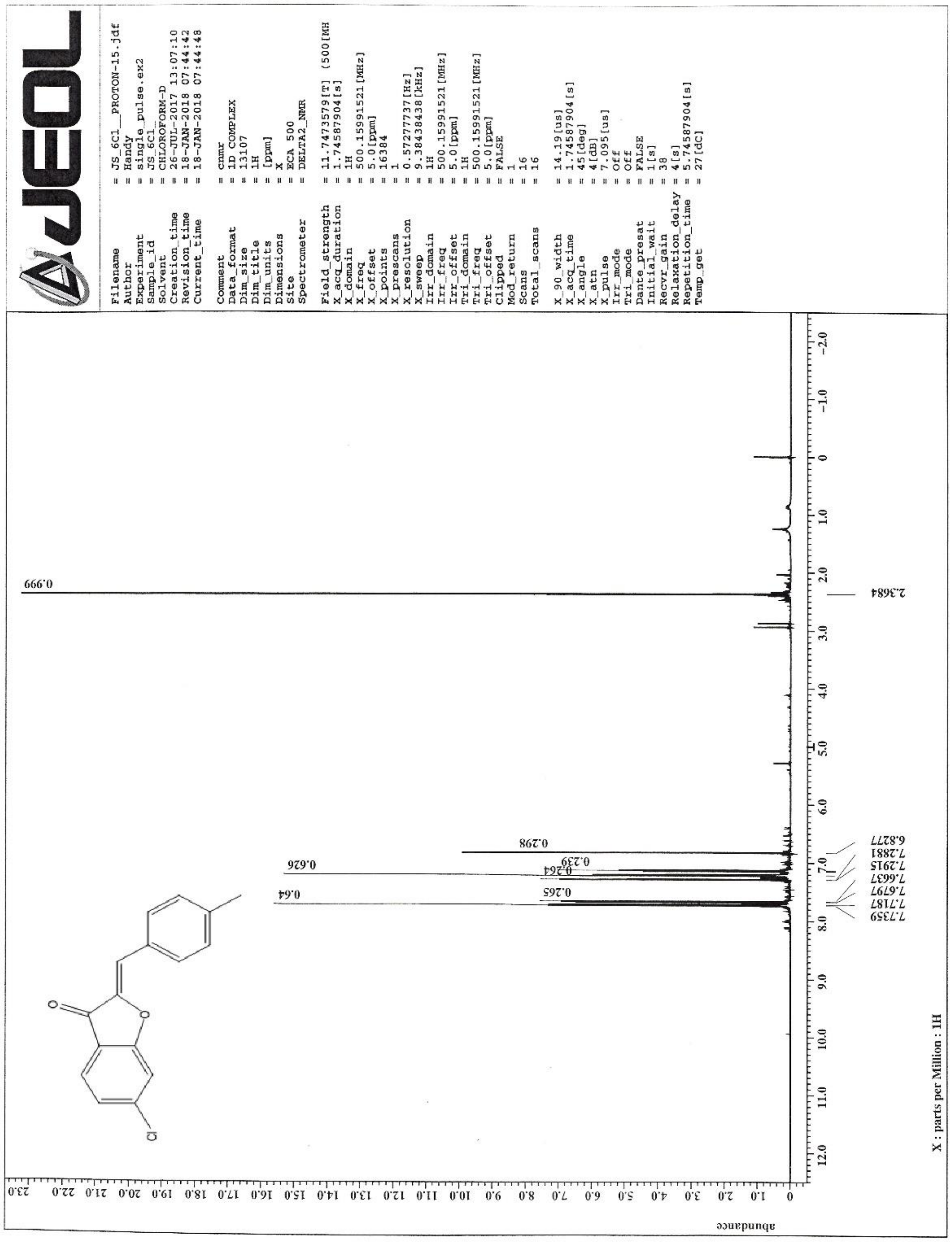

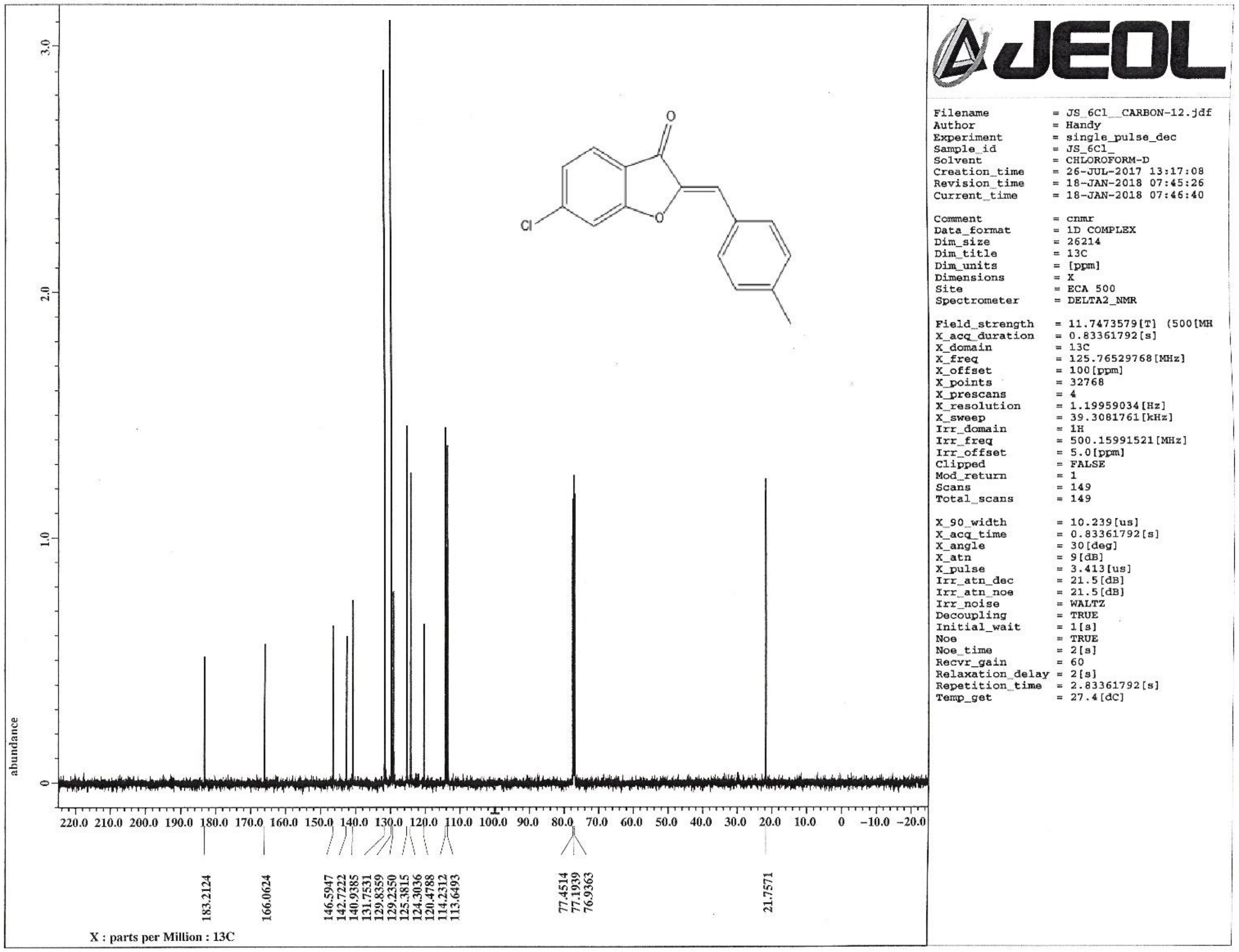
Filename = JS_6Cl__CARBON-12.jdf
Author = Handy
Experiment = single_pulse_dec
Sample_id = JS_6Cl_
Solvent = CHLOROFORM-D
Creation_time = 26-JUL-2017 13:17:08
Revision_time = 18-JAN-2018 07:45:26
Current_time = 18-JAN-2018 07:46:40
Comment = cnmr
Data_format = 1D COMPLEX
Dim_size = 26214
Dim_title = 13C
Dim_units = [ppm]
Dimensions = X
Site = ECA 500
Spectrometer = DELTA2_NMR
Field_strength = 11.7473579[T] (500[MH
X_acq_duration = 0.83361792[s]
X_domain = 13C
X_freq = 125.76529768[MHz]
X_offset = 100[ppm]
X_points = 32768
X_prescans = 4
X_resolution = 1.19959034[Hz]
X_sweep = 39.3081761[kHz]
Irr_domain = 1H
Irr_freq = 500.15991521[MHz]
Irr_offset = 5.0[ppm]
Clipped = FALSE
Mod_return = 1
Scans = 149
Total_scans = 149
X_90_width = 10.239[us]
X_acq_time = 0.83361792[s]
X_angle = 30[deg]
X_atn = 9[dB]
X_pulse = 3.413[us]
Irr_atn_dec = 21.5[dB]
Irr_atn_noe = 21.5[dB]
Irr_noise = WALTZ
Decoupling = TRUE
Initial_wait = 1[s]
Noe = TRUE
Noe_time = 2[s]
Recvr_gain = 60
Relaxation_delay = 2[s]
Repetition_time = 2.83361792[s]
Temp_get = 27.4[dC]
183.2124
166.0624
146.5947
142.7222
140.9385
131.7531
129.8359
129.2350
125.3815
124.3036
120.4788
114.2312
113.6493
77.4514
77.1939
76.9363
21.7571
abundance
X : parts per Million : 13C

C:\Sync\ShimadzuGCMS\Documents\Handy\Schmitt\6Cl.qgd

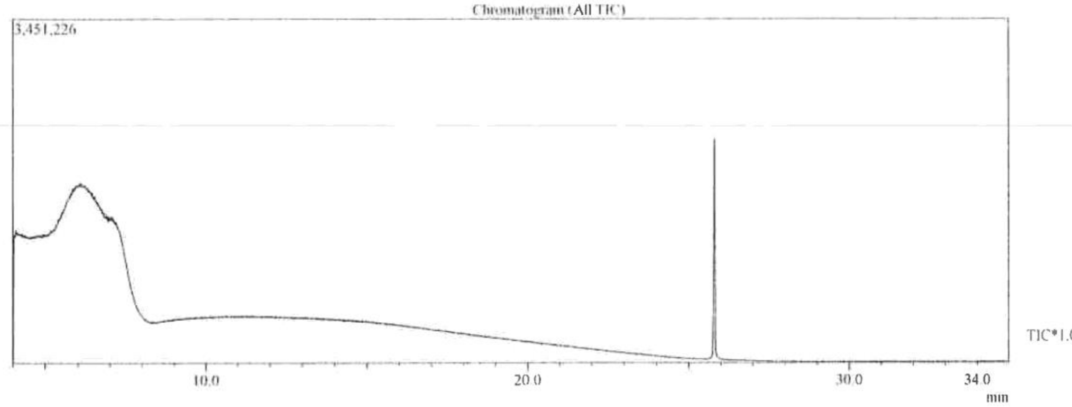

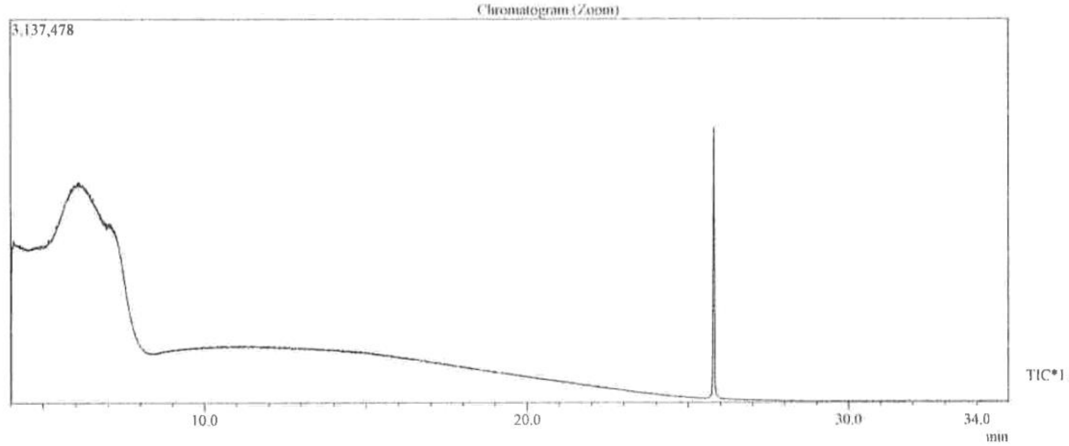

Spectrum

Line#:1 R Time:25.800(Scan#:4361)
MassPeaks:147
RawMode:Single 25.800(4361) BasePeak:255.10(150625)
BG Mode:None Group 1 - Event 1 Scan

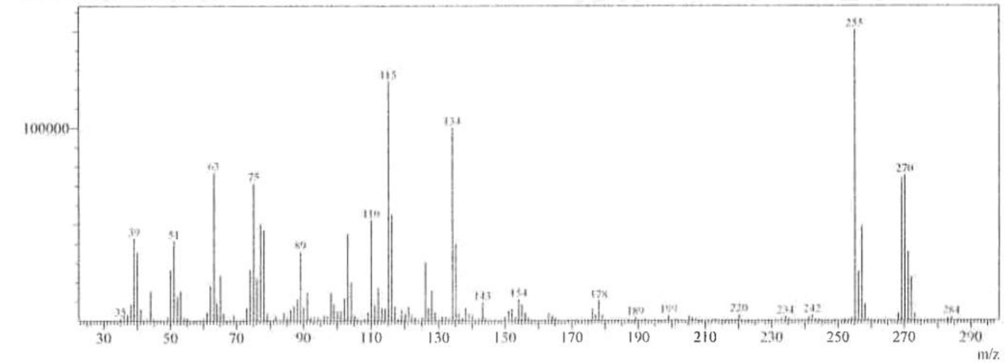

2-(4-methylbenzylidene)-1-(7’-chlorobenzofuran-3-one) [**7**]

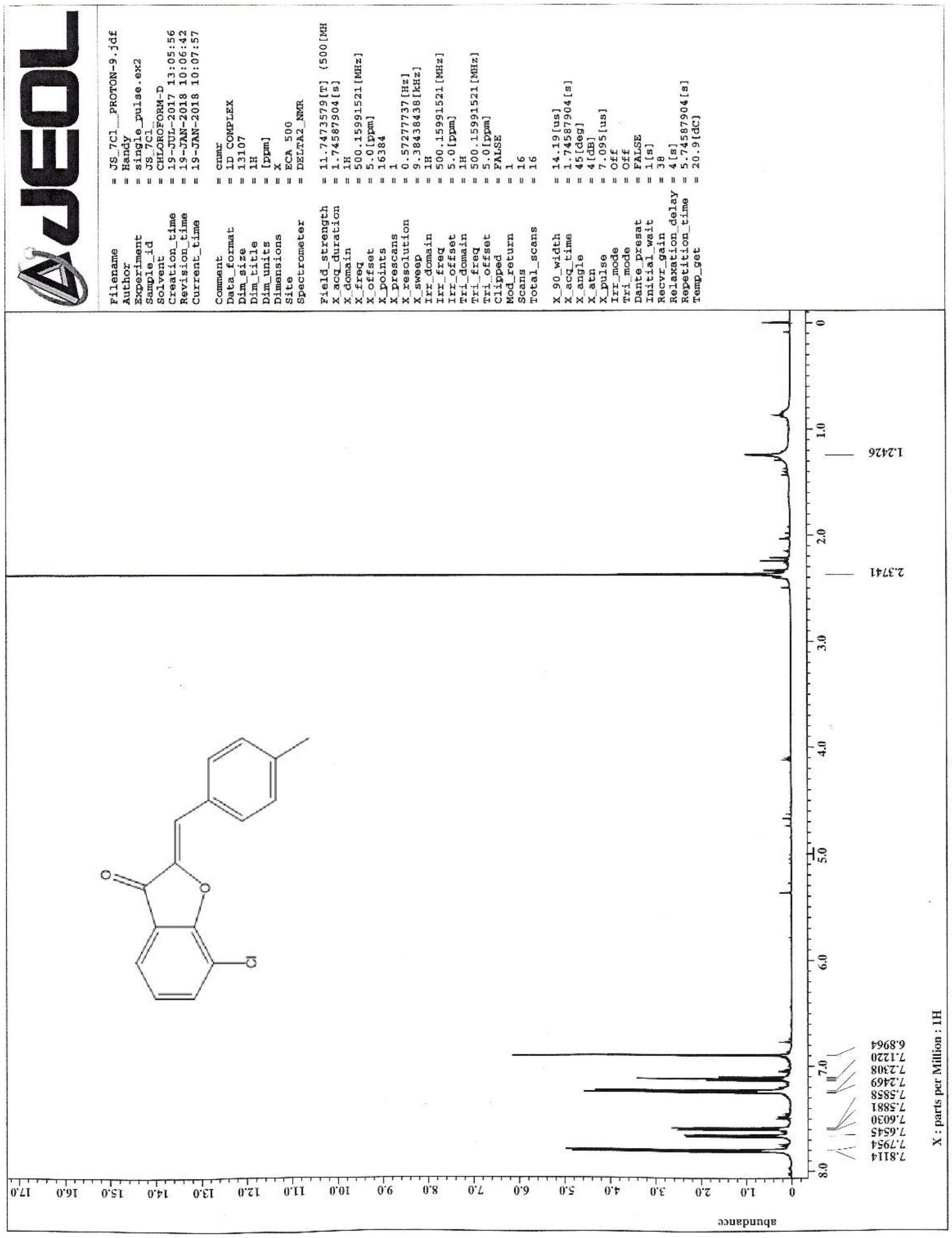

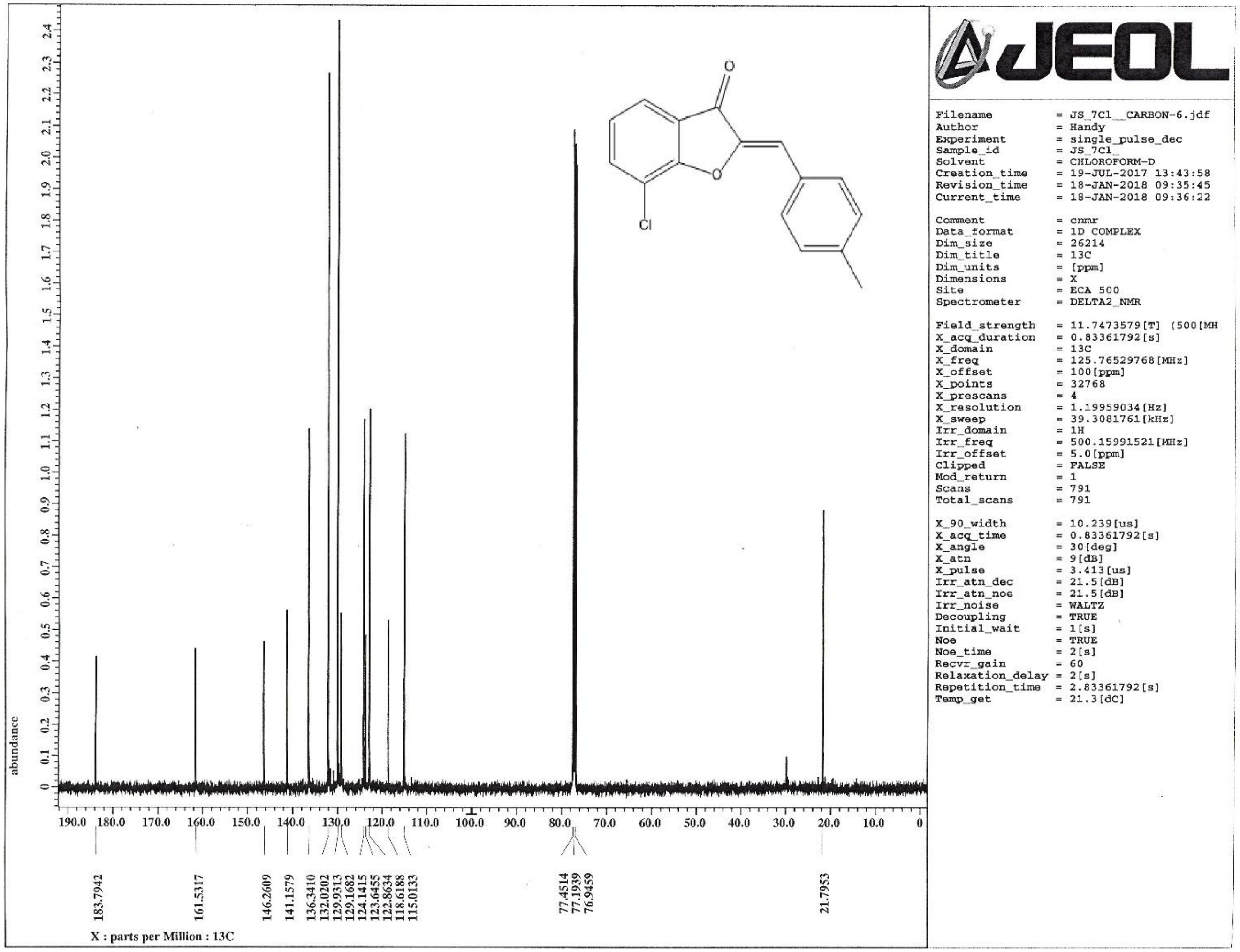
JEOL
Filename = JS_7Cl__CARBON-6.jdf
Author = Handy
Experiment = single_pulse_dec
Sample_id = JS_7Cl_
Solvent = CHLOROFORM-D
Creation_time = 19-JUL-2017 13:43:58
Revision_time = 18-JAN-2018 09:35:45
Current_time = 18-JAN-2018 09:36:22
Comment = cnmr
Data_format = 1D COMPLEX
Dim_size = 26214
Dim_title = 13C
Dim_units = [ppm]
Dimensions = X
Site = ECA 500
Spectrometer = DELTA2_NMR
Field_strength = 11.7473579[T] (500[MH
X_acq_duration = 0.83361792[s]
X_domain = 13C
X_freq = 125.76529768[MHz]
X_offset = 100[ppm]
X_points = 32768
X_prescans = 4
X_resolution = 1.19959034[Hz]
X_sweep = 39.3081761[kHz]
Irr_domain = 1H
Irr_freq = 500.15991521[MHz]
Irr_offset = 5.0[ppm]
Clipped = FALSE
Mod_return = 1
Scans = 791
Total_scans = 791
X_90_width = 10.239[us]
X_acq_time = 0.83361792[s]
X_angle = 30[deg]
X_atn = 9[dB]
X_pulse = 3.413[us]
Irr_atn_dec = 21.5[dB]
Irr_atn_noe = 21.5[dB]
Irr_noise = WALTZ
Decoupling = TRUE
Initial_wait = 1[s]
Noe = TRUE
Noe_time = 2[s]
Recvr_gain = 60
Relaxation_delay = 2[s]
Repetition_time = 2.83361792[s]
Temp_get = 21.3[dC]
O
Cl
abundance
183.7942
161.5317
146.2609
141.1579
136.3410
132.0202
129.9313
129.1682
124.1415
123.6455
122.8634
118.6188
115.0133
77.4514
77.1939
76.9459
21.7953
X : parts per Million : 13C

C:\Sync\ShimadzuGCMS\Documents\Handy\Schmitt\7Cl.qgd

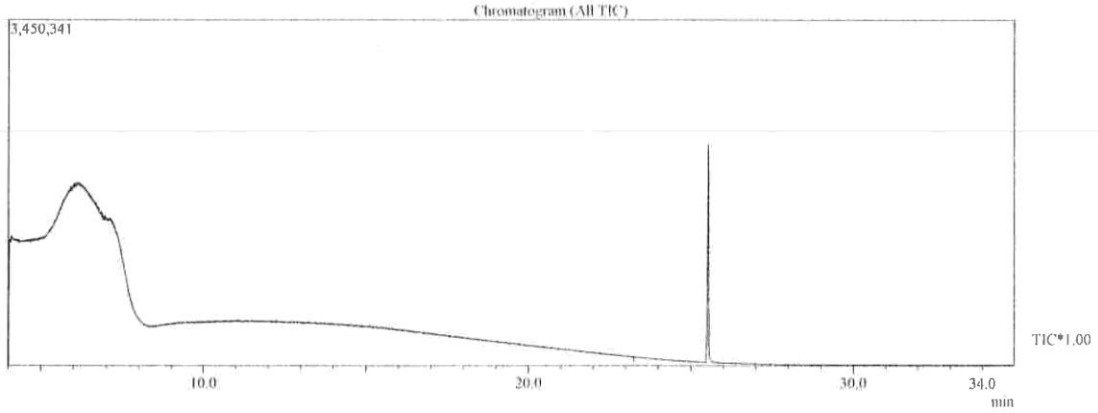

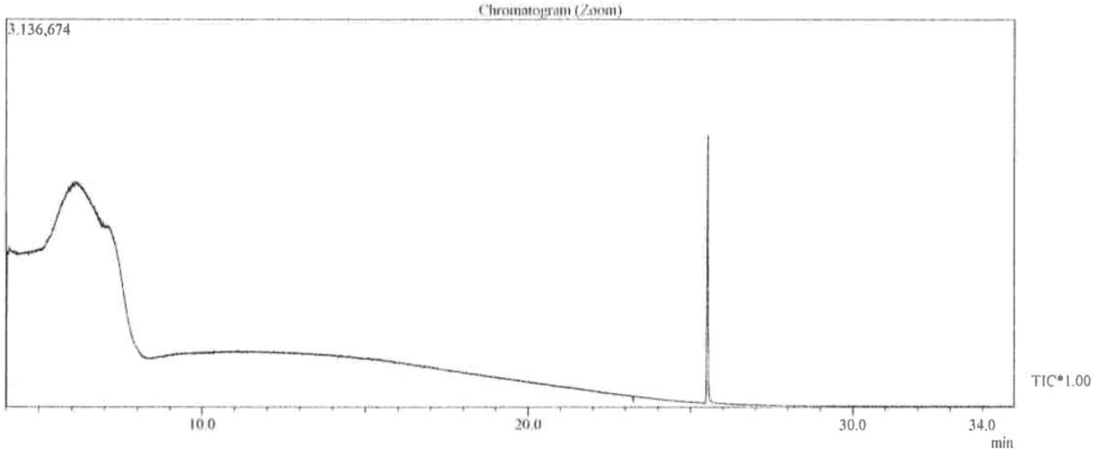

Spectrum

Line#:1 R.Time:25.530(Scan#:4307)
MassPeaks:145
RawMode:Single 25.530(4307) BasePeak:255.15(158383)
BG Mode:None Group 1 - Event 1 Scan

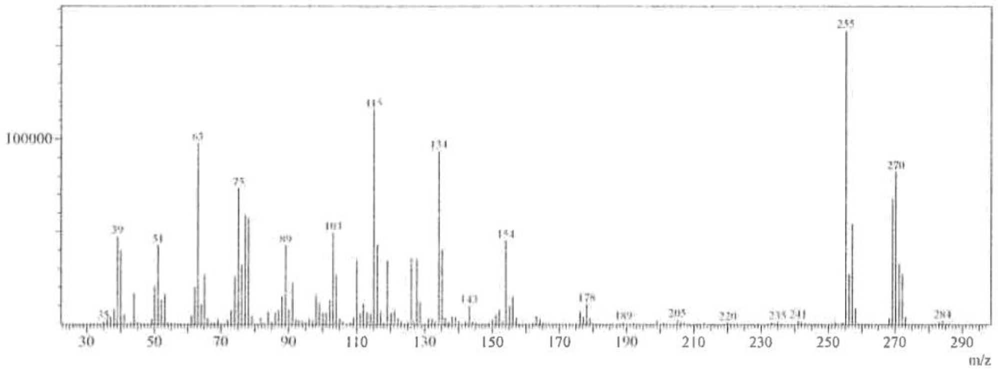

2-(4-methylbenzylidene)-1-(5’-bromobenzofuran-3-one) [**8**]

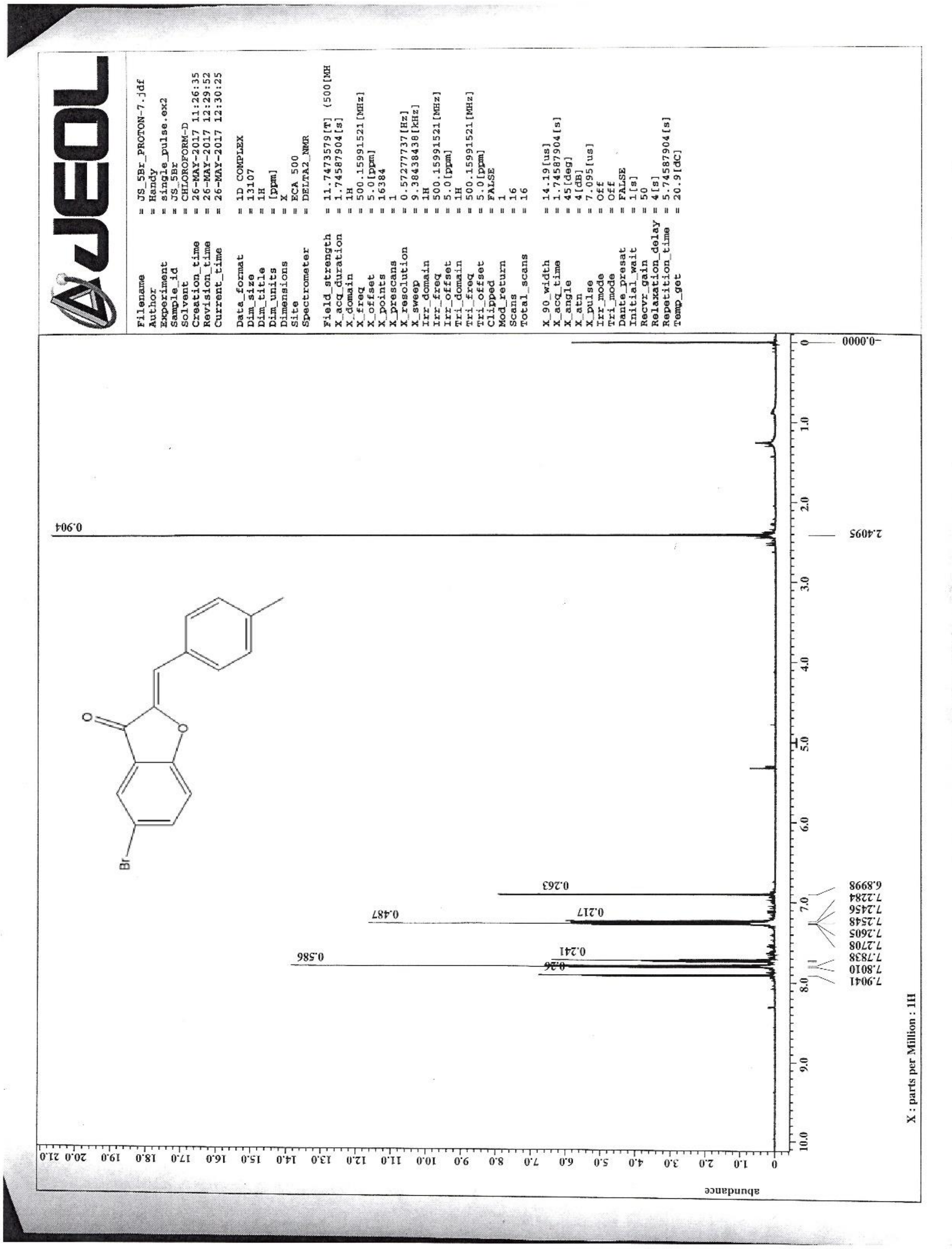

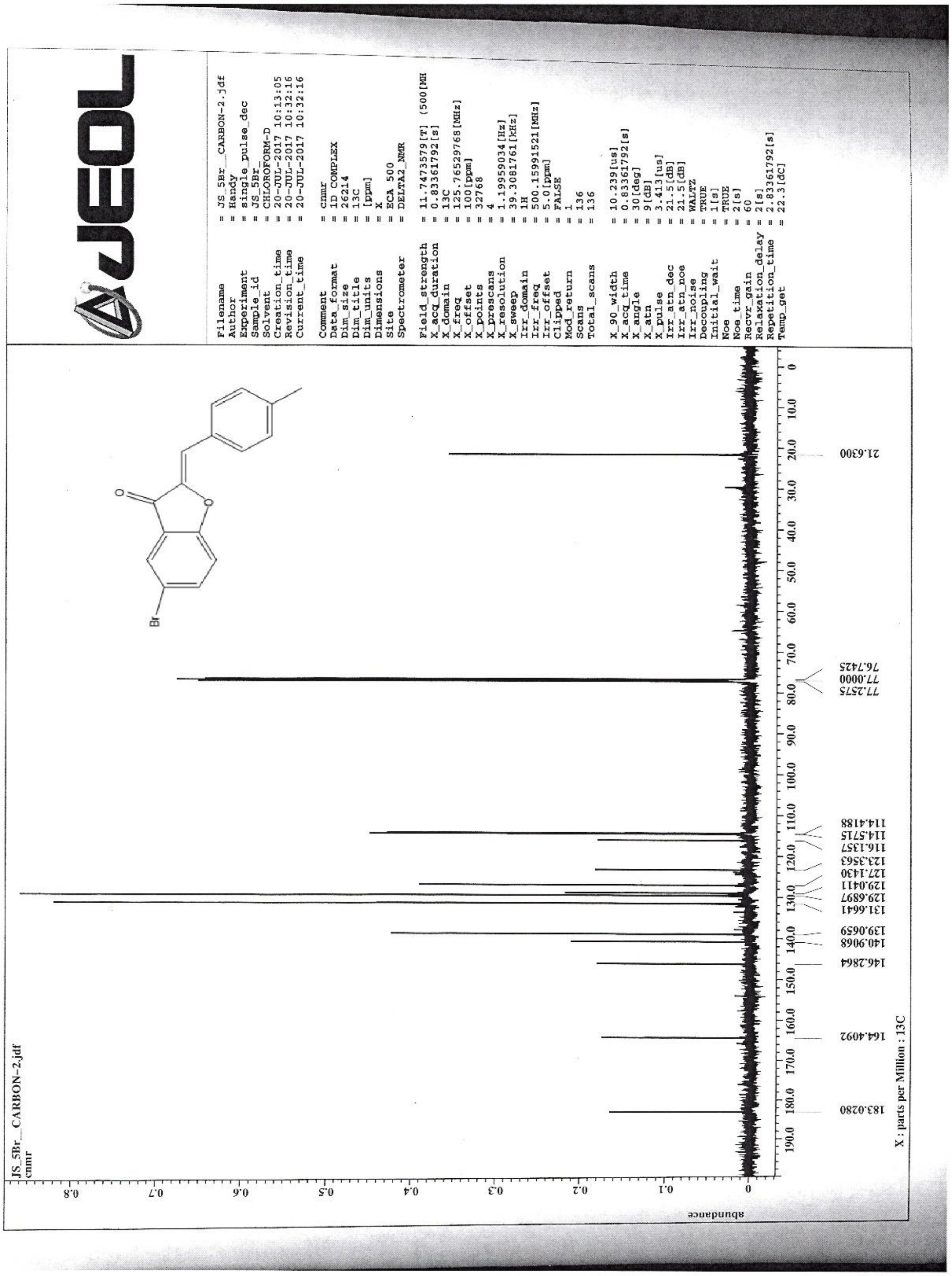

C:\Sync\ShimadzuGCMS\Documents\Handy\Schmitt\5Br.qgd

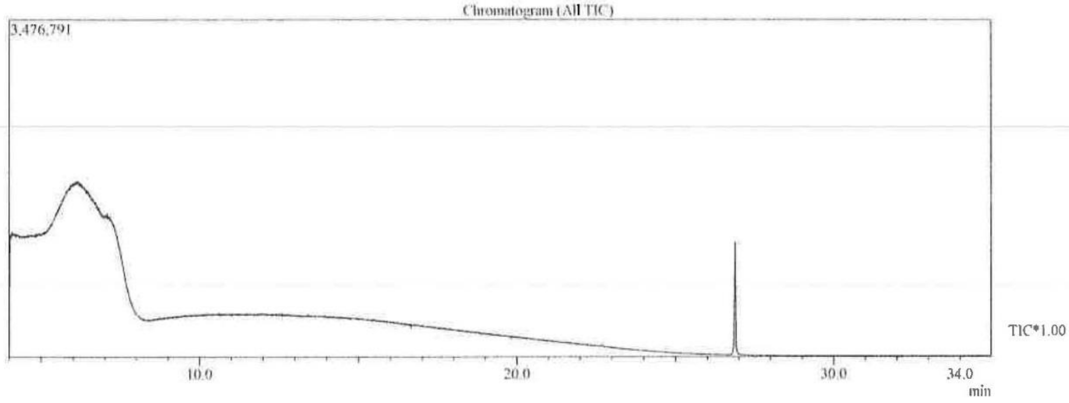

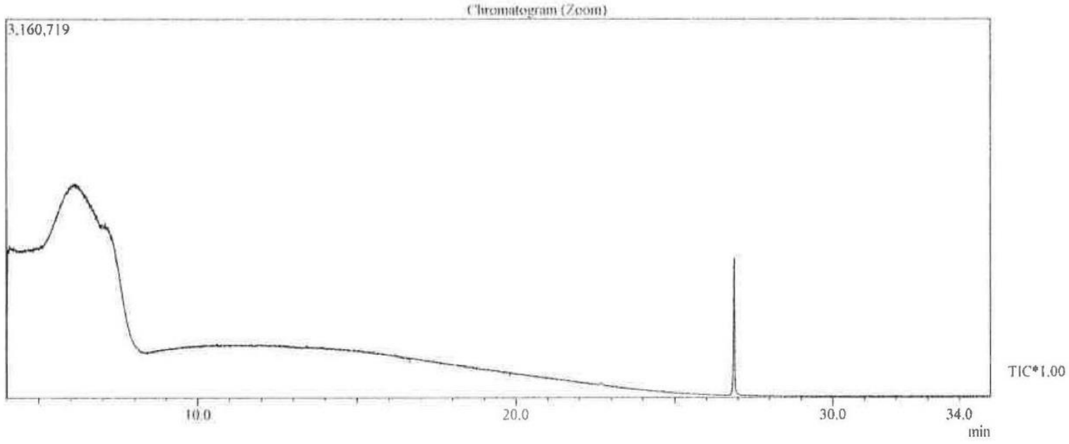

Spectrum

Line#:1 R.Time:26.900(Scan#:4581)
MassPeaks:82
RawMode:Single 26.900(4581) BasePeak:63.00(16604)
BG Mode:None Group 1 - Event 1 Scan

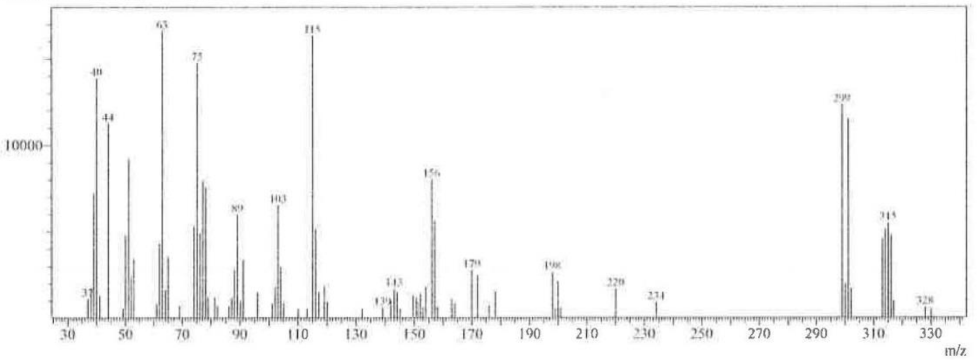

2-(4-methylbenzylidene)-1-(6’-bromobenzofuran-3-one) [**9**]

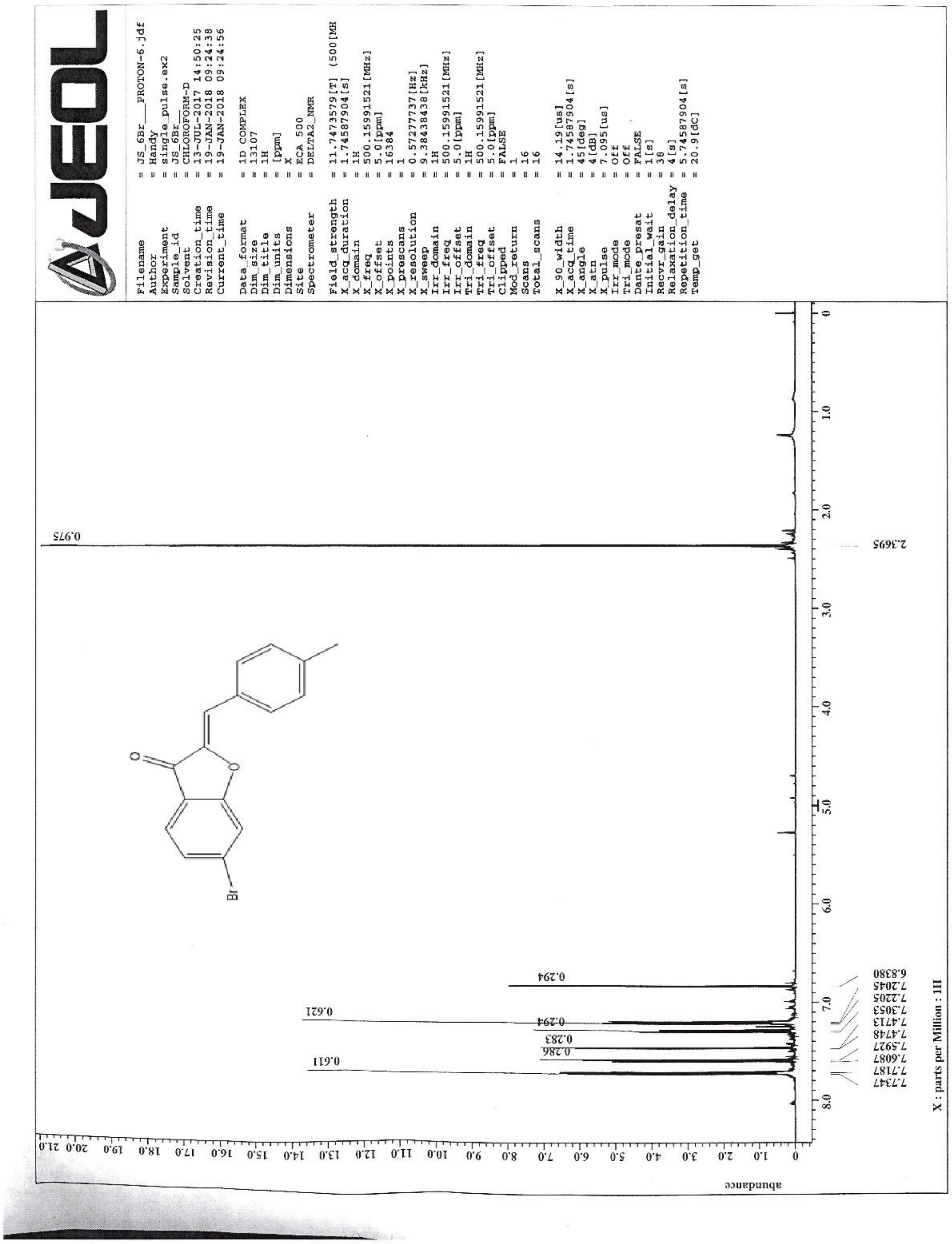

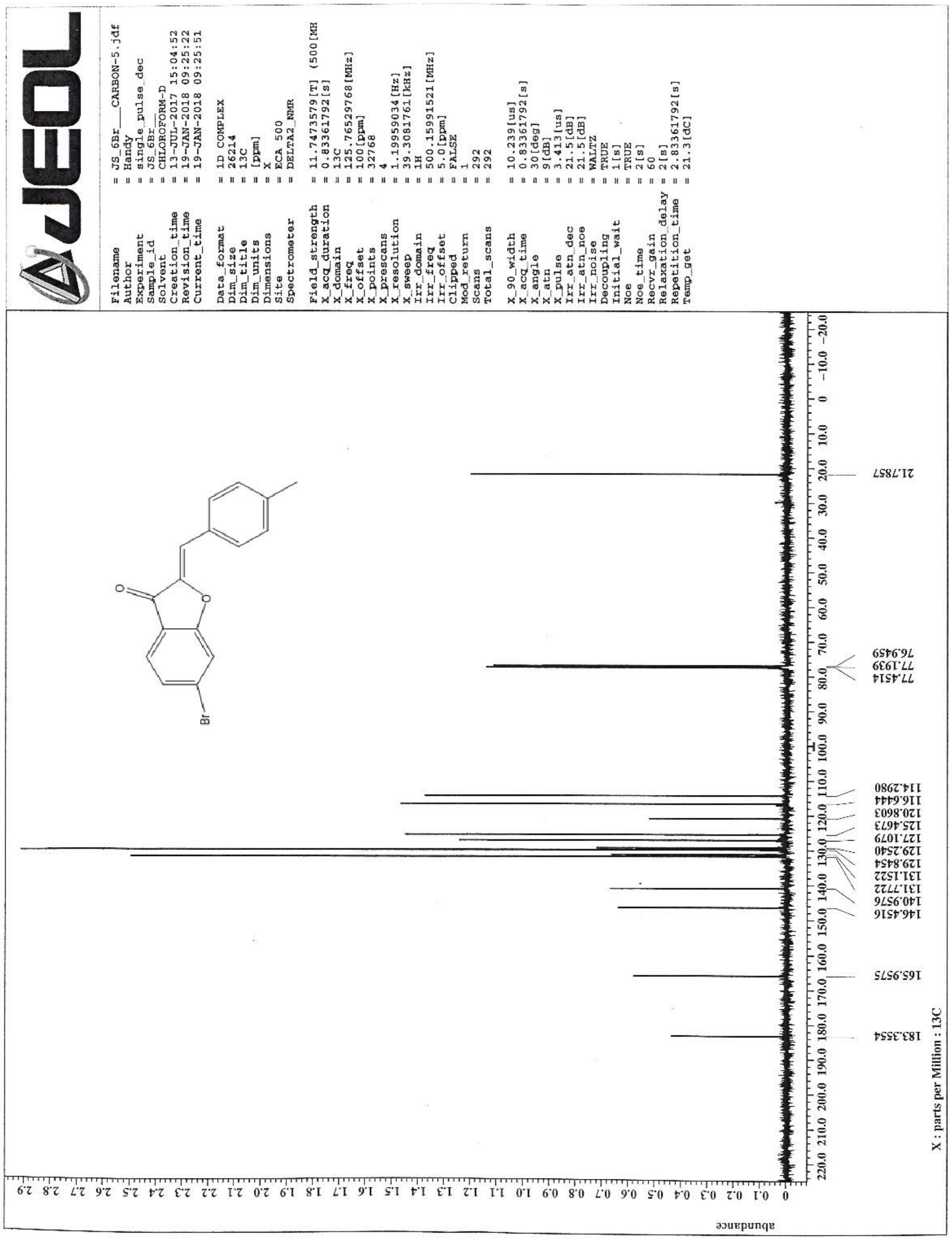

JEOL
Filename = JS_6Br___CARBON-5.jdf
Author = Handy
Experiment = single_pulse_dec
Sample_id = JS_6Br___
Solvent = CHLOROFORM-D
Creation_time = 13-JUL-2017 15:04:52
Revision_time = 19-JAN-2018 09:25:22
Current_time = 19-JAN-2018 09:25:51
Data_format = 1D COMPLEX
Dim_size = 26214
Dim_title = 13C
Dim_units = [ppm]
Dimensions = X
Site = ECA 500
Spectrometer = DELTA2_NMR
Field_strength = 11.7473579[T] (500[MH
X_acq_duration = 0.83361792[s]
X_domain = 13C
X_freq = 125.76529768[MHz]
X_offset = 100[ppm]
X_points = 32768
X_prescans = 4
X_resolution = 1.19959034[Hz]
X_sweep = 39.3081761[kHz]
Irr_domain = 1H
Irr_freq = 500.15991521[MHz]
Irr_offset = 5.0[ppm]
Clipped = FALSE
Mod_return = 1
Scans = 292
Total_scans = 292
X_90_width = 10.239[us]
X_acq_time = 0.83361792[s]
X_angle = 30[deg]
X_atn = 9[dB]
X_pulse = 3.413[us]
Irr_atn_dec = 21.5[dB]
Irr_atn_noe = 21.5[dB]
Irr_noise = WALTZ
Decoupling = TRUE
Initial_wait = 1[s]
Noe = TRUE
Noe_time = 2[s]
Recvr_gain = 60
Relaxation_delay = 2[s]
Repetition_time = 2.83361792[s]
Temp_get = 21.3[dC]
Br
O
183.3554
165.9575
146.4516
140.9576
131.7722
131.1522
129.8454
129.2540
127.1079
125.4673
120.8603
116.6444
114.2980
77.4514
77.1939
76.9459
21.7857
X : parts per Million : 13C
abundance

C:\Sync\ShimadzuGCMS\Documents\Handy\Schmitt\6Br.qgd

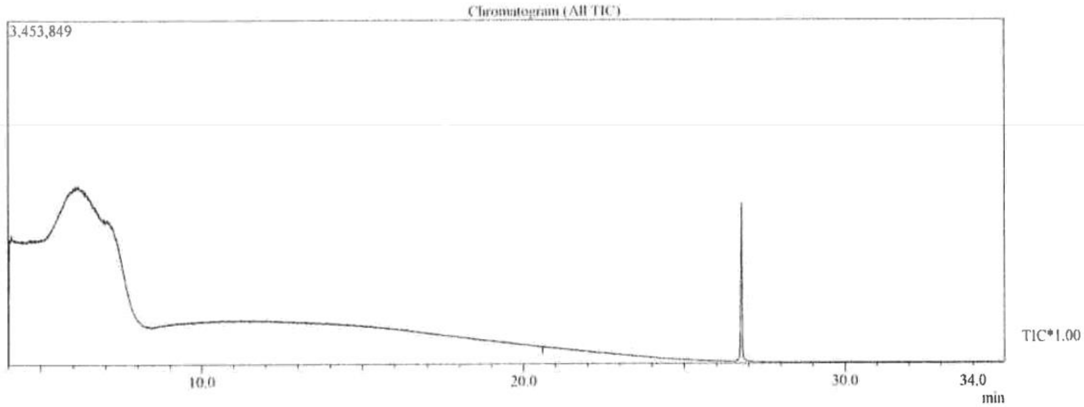

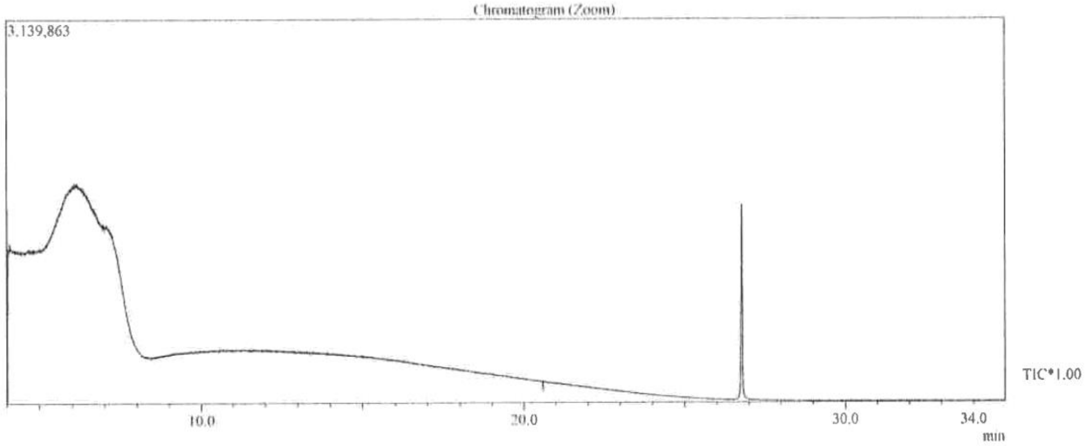

Spectrum

Line#:1 R.Time:26.765(Scan#:4554)
MassPeaks:127
RawMode:Single 26.765(4554) BasePeak:75.05(71248)
BG Mode:None Group 1 - Event 1 Scan

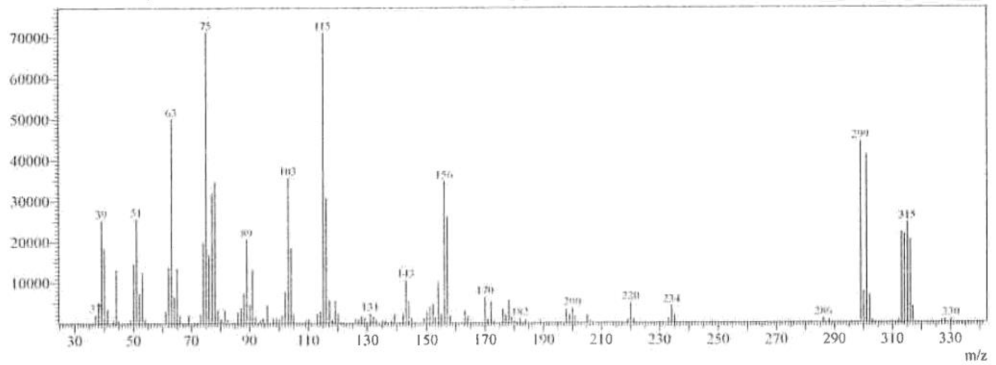

2-(4-methylbenzylidene)-1-(7’-bromobenzofuran-3-one) [**10**]

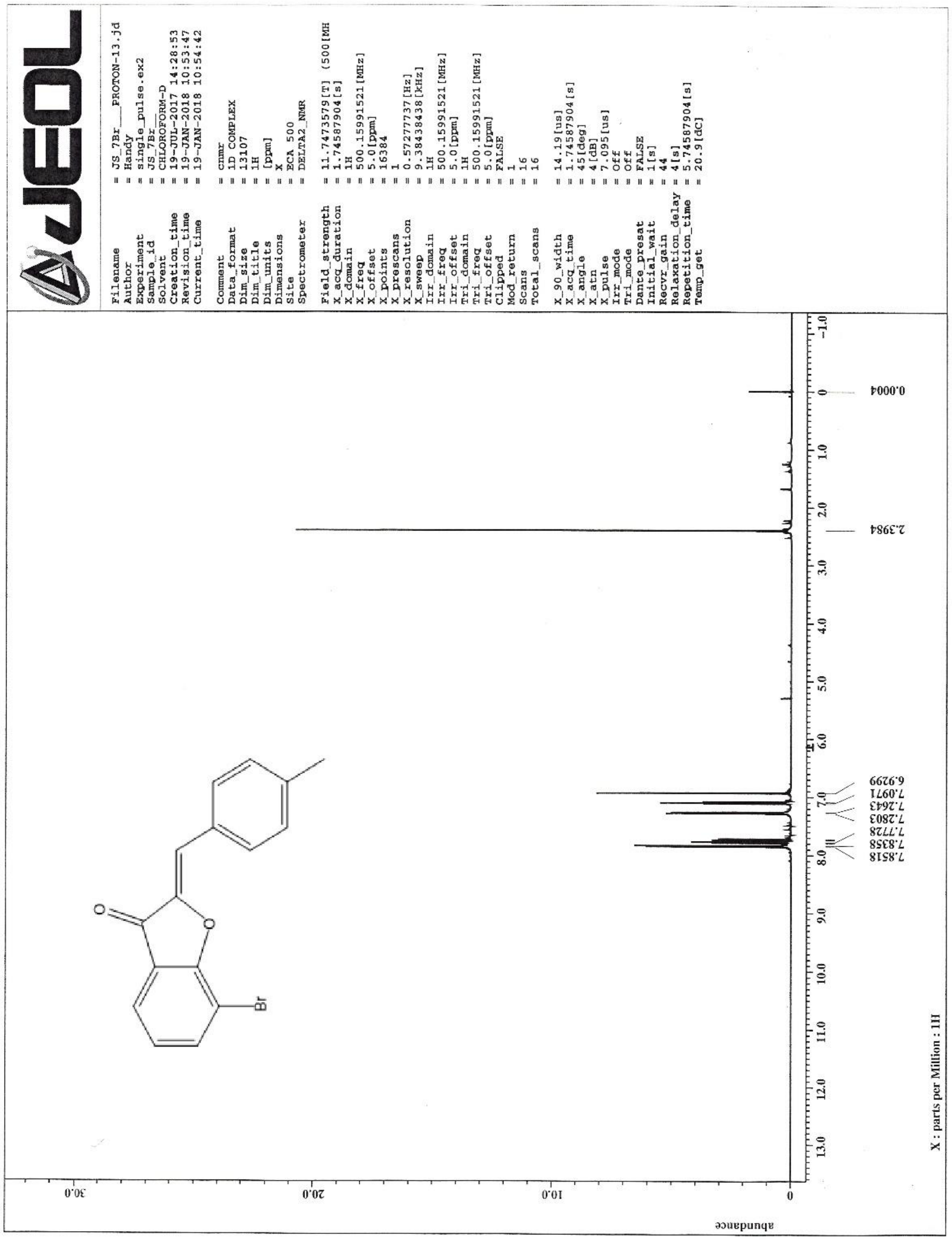

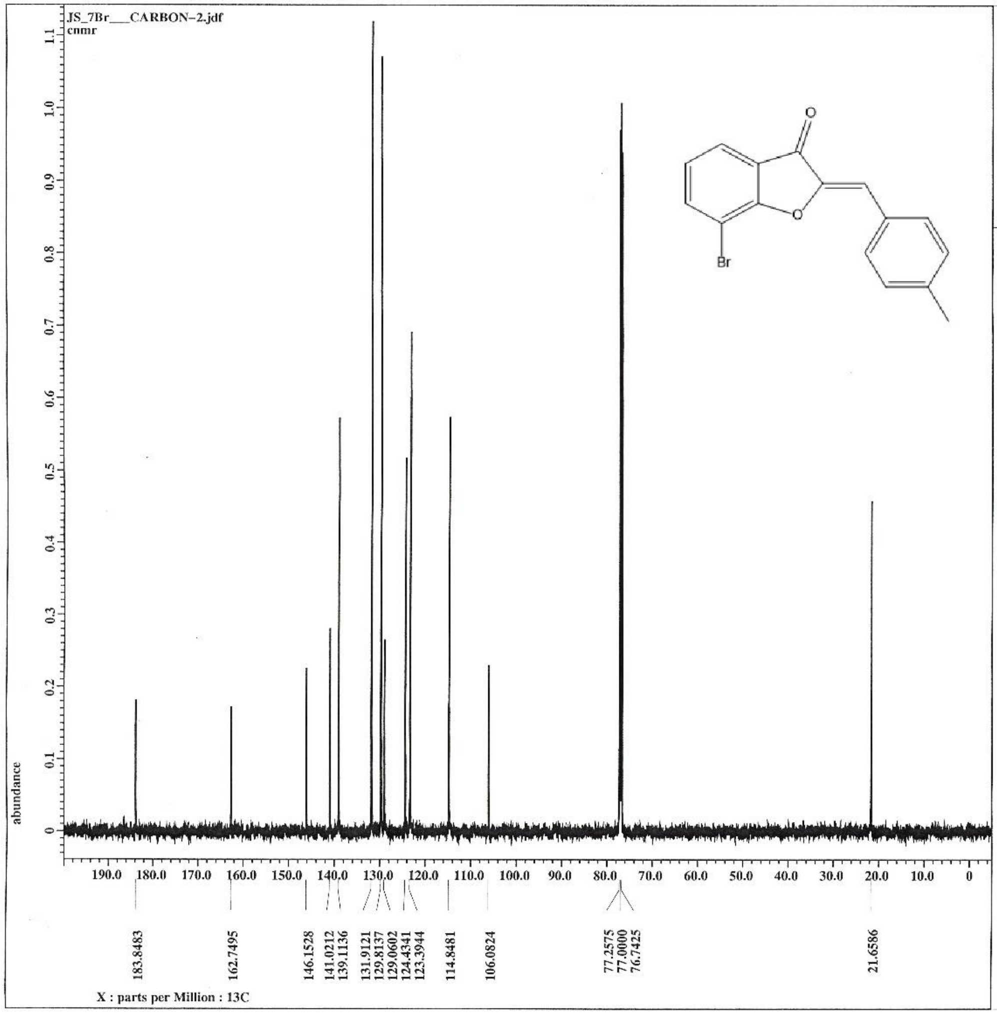

JEOL

```
Filename          = JS_7Br___CARBON-2.jdf
Author            = Handy
Experiment        = single_pulse_dec
Sample_id         = JS_7Br__
Solvent           = CHLOROFORM-D
Creation_time     = 19-JUL-2017 14:48:15
Revision_time     = 19-JUL-2017 15:07:26
Current_time      = 19-JUL-2017 15:07:26

Comment           = cnmr
Data_format       = 1D COMPLEX
Dim_size          = 26214
Dim_title         = 13C
Dim_units         = [ppm]
Dimensions        = X
Site              = ECA 500
Spectrometer      = DELTA2_NMR

Field_strength    = 11.7473579[T] (500[MH
X_acq_duration    = 0.83361792[s]
X_domain          = 13C
X_freq            = 125.76529768[MHz]
X_offset          = 100[ppm]
X_points          = 32768
X_prescans        = 4
X_resolution      = 1.19959034[Hz]
X_sweep           = 39.3081761[kHz]
Irr_domain        = 1H
Irr_freq          = 500.15991521[MHz]
Irr_offset        = 5.0[ppm]
Clipped           = TRUE
Mod_return        = 1
Scans             = 337
Total_scans       = 337

X_90_width        = 10.239[us]
X_acq_time        = 0.83361792[s]
X_angle           = 30[deg]
X_atn             = 9[dB]
X_pulse           = 3.413[us]
Irr_atn_dec       = 21.5[dB]
Irr_atn_noe       = 21.5[dB]
Irr_noise         = WALTZ
Decoupling        = TRUE
Initial_wait      = 1[s]
Noe               = TRUE
Noe_time          = 2[s]
Recvr_gain        = 60
Relaxation_delay  = 2[s]
Repetition_time   = 2.83361792[s]
Temp_get          = 21.3[dC]
```

C:\Sync\ShimadzuGCMS\Documents\Handy\Schmitt\7Br.qgd

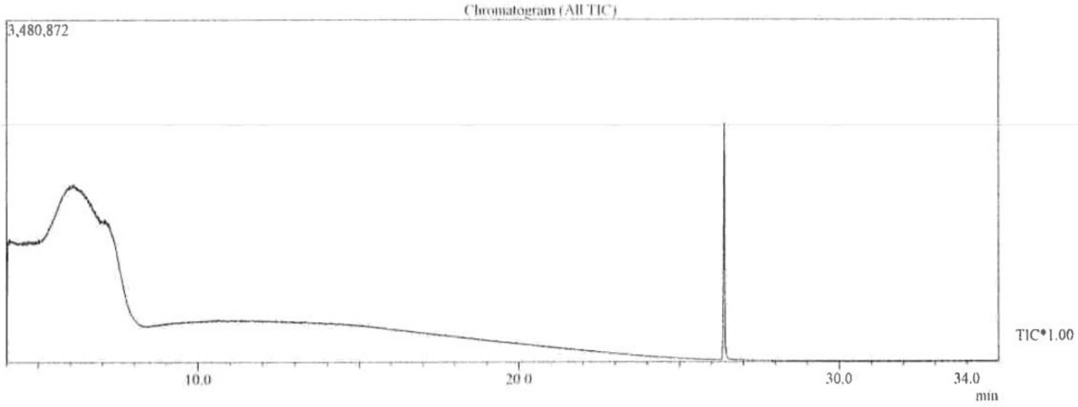

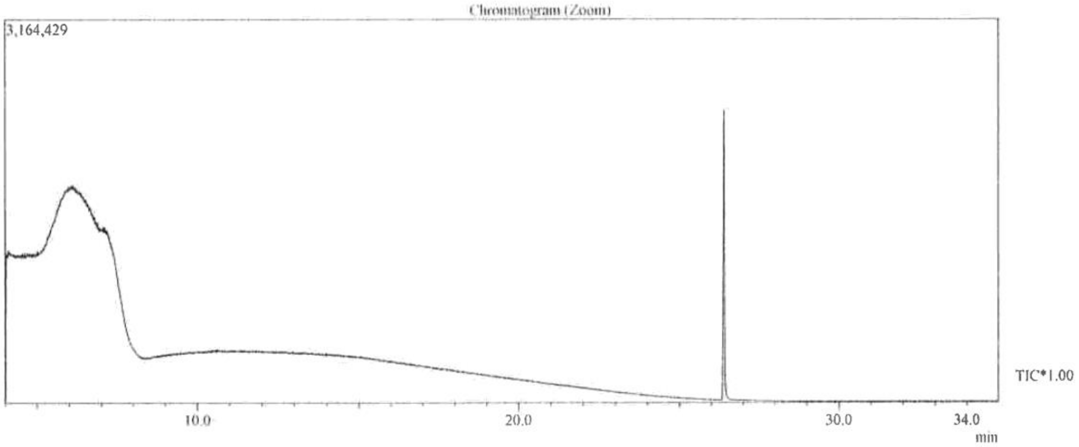

Spectrum

Line#:1 R.Time:26.405(Scan#:4482)
MassPeaks:157
RawMode:Single 26.405(4482) BasePeak:75.05(158668)
BG Mode:None Group 1 - Event 1 Scan

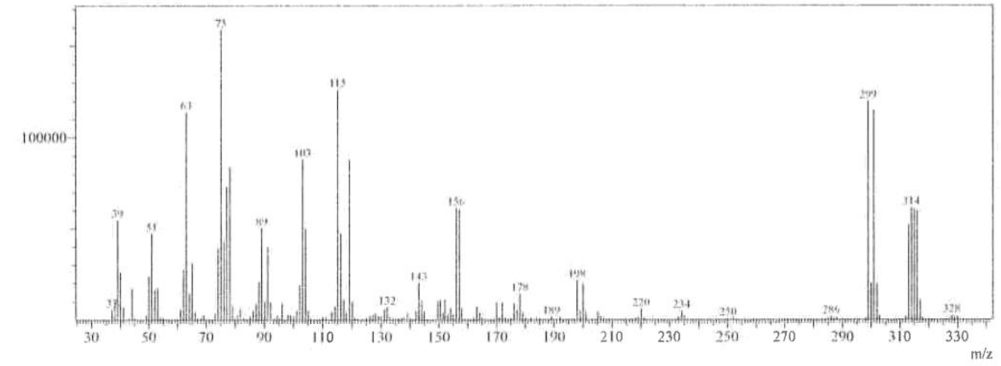

2-(4-methylbenzylidene)-1-(5’-methylbenzofuran-3-one) [**11**]

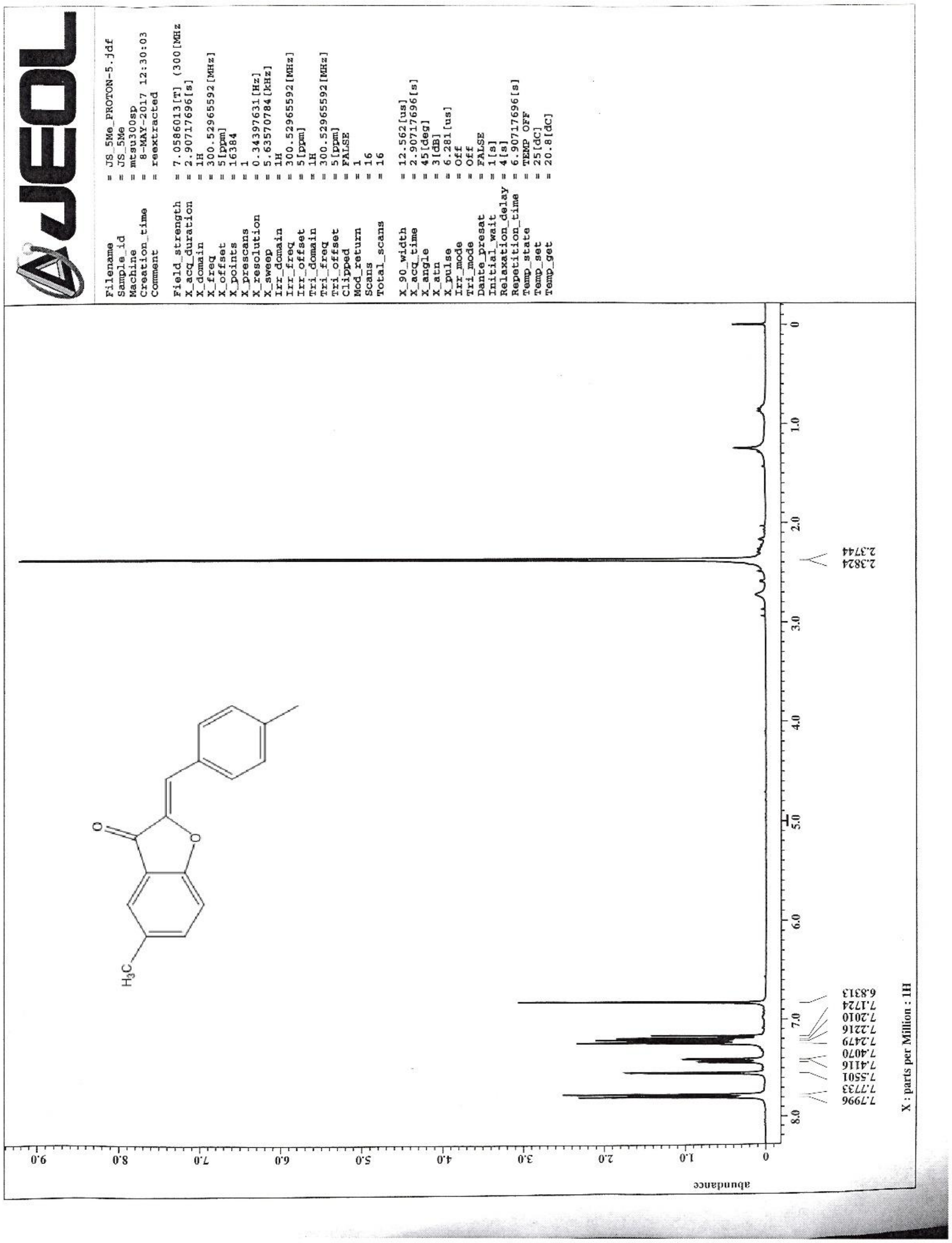

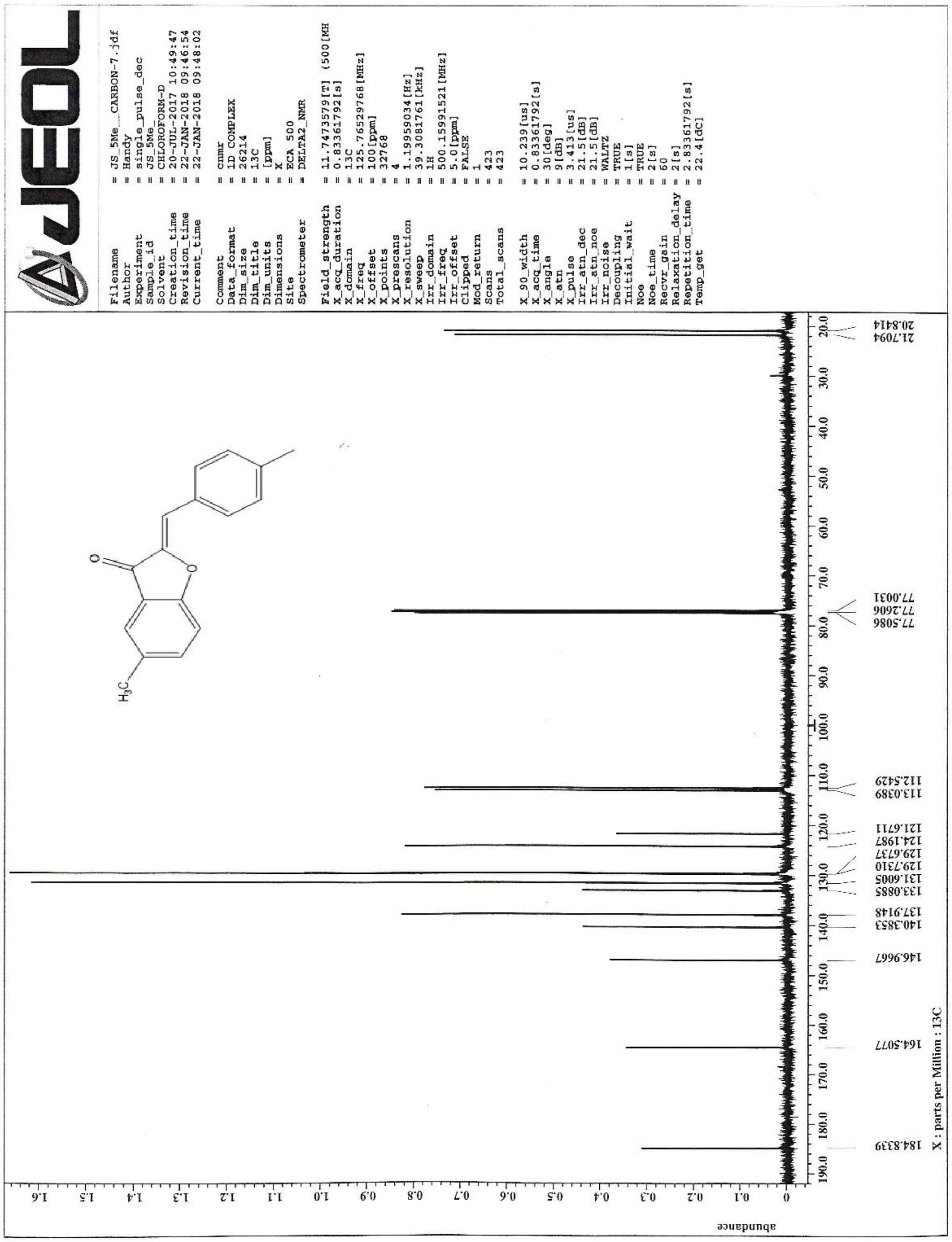
JEOL
Filename = JS_5Me__CARBON-7.jdf
Author = Handy
Experiment = single_pulse_dec
Sample_id = JS_5Me_
Solvent = CHLOROFORM-D
Creation_time = 20-JUL-2017 10:49:47
Revision_time = 22-JAN-2018 09:46:54
Current_time = 22-JAN-2018 09:48:02
Comment = cnmr
Data_format = 1D COMPLEX
Dim_size = 26214
Dim_title = 13C
Dim_units = [ppm]
Dimensions = X
Site = ECA 500
Spectrometer = DELTA2_NMR
Field_strength = 11.7473579[T] (500[MH
X_acq_duration = 0.83361792[s]
X_domain = 13C
X_freq = 125.76529768[MHz]
X_offset = 100[ppm]
X_points = 32768
X_prescans = 4
X_resolution = 1.19959034[Hz]
X_sweep = 39.3081761[kHz]
Irr_domain = 1H
Irr_freq = 500.15991521[MHz]
Irr_offset = 5.0[ppm]
Clipped = FALSE
Mod_return = 1
Scans = 423
Total_scans = 423
X_90_width = 10.239[us]
X_acq_time = 0.83361792[s]
X_angle = 30[deg]
X_atn = 9[dB]
X_pulse = 3.413[us]
Irr_atn_dec = 21.5[dB]
Irr_atn_noe = 21.5[dB]
Irr_noise = WALTZ
Decoupling = TRUE
Initial_wait = 1[s]
Noe = TRUE
Noe_time = 2[s]
Recvr_gain = 60
Relaxation_delay = 2[s]
Repetition_time = 2.83361792[s]
Temp_get = 22.4[dC]
H3C
O
20.8414
21.7094
77.0031
77.2606
77.5086
112.5429
113.0389
121.6711
124.1987
129.6737
129.7310
131.6005
133.0885
137.9148
140.3853
146.9667
164.5077
184.8339
X : parts per Million : 13C
abundance

C:\Sync\ShimadzuGCMS\Documents\Handy\Schmitt\5Me.qgd

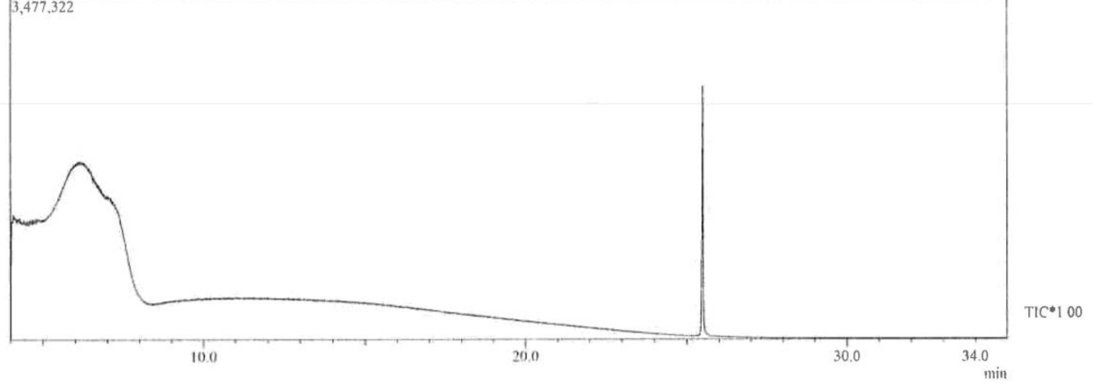

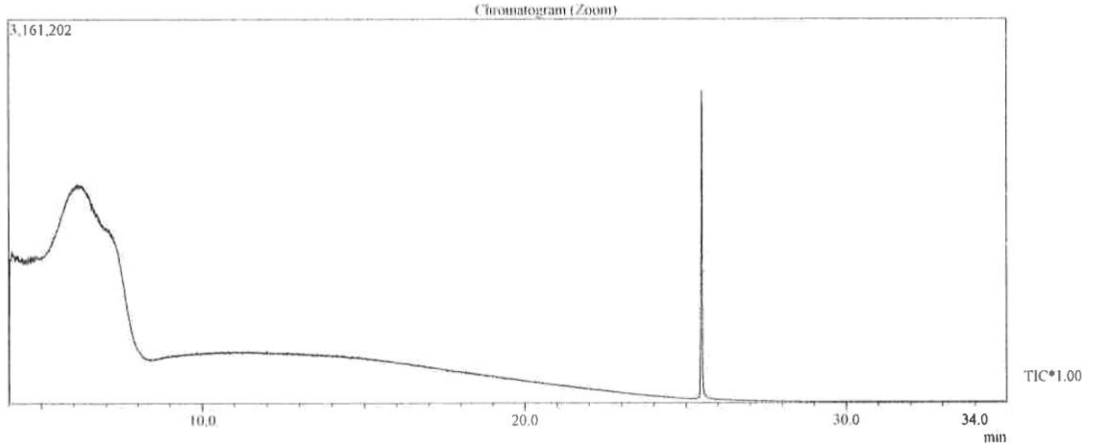

Spectrum

Line#:1 R.Time:25.505(Scan#:4302)
MassPeaks:105
RawMode:Single 25.505(4302) BasePeak:235.20(73345)
BG Mode:None Group 1 - Event 1 Scan

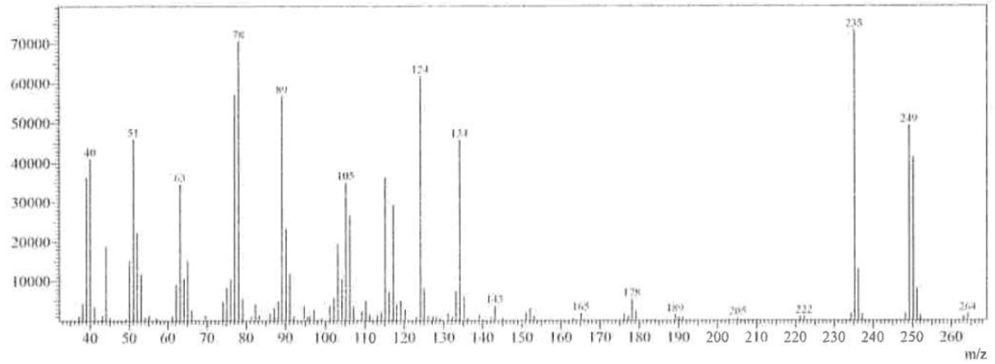

2-(4-methylbenzylidene)-1-(5’,6’-dimethylbenzofuran-3-one) [**12**]

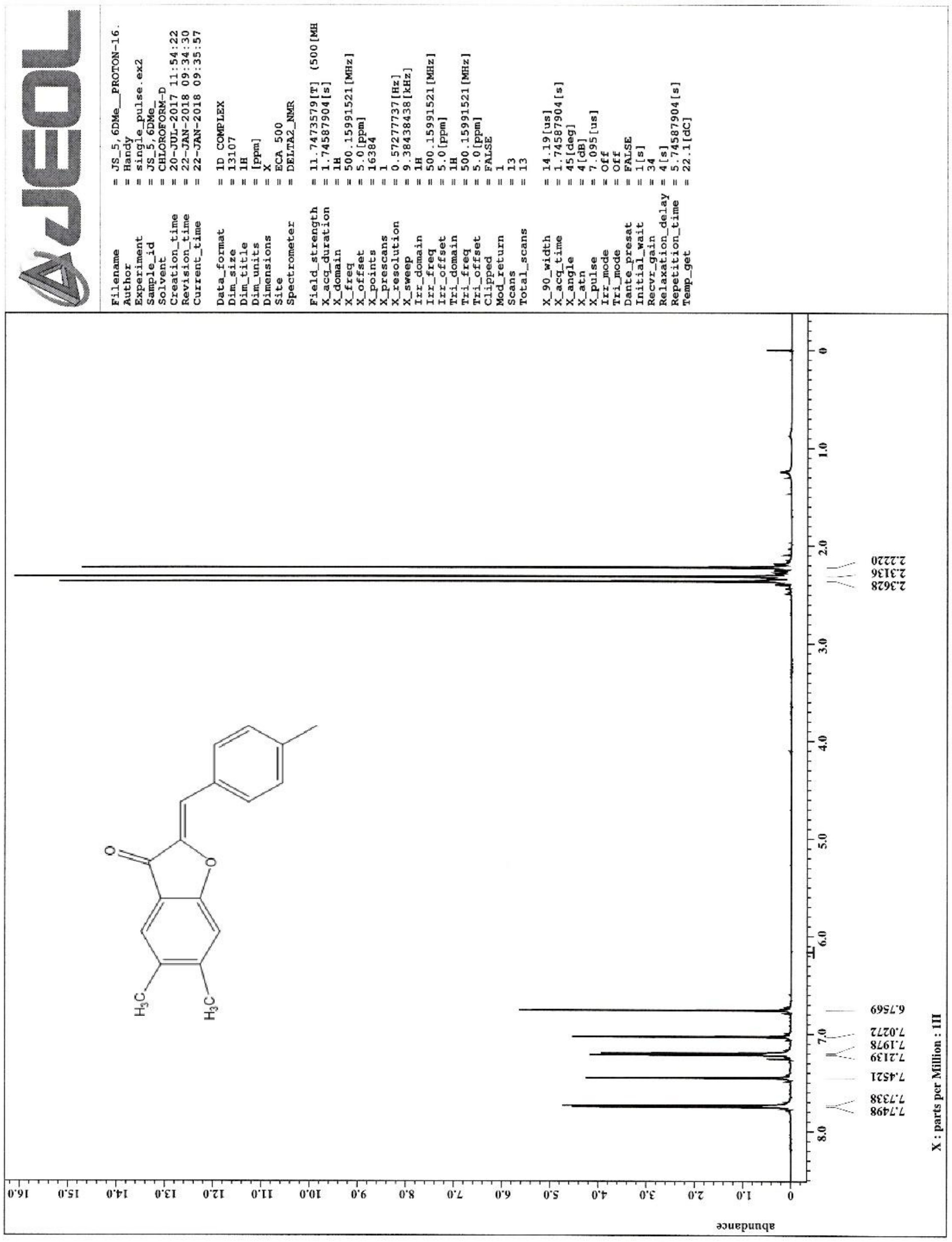

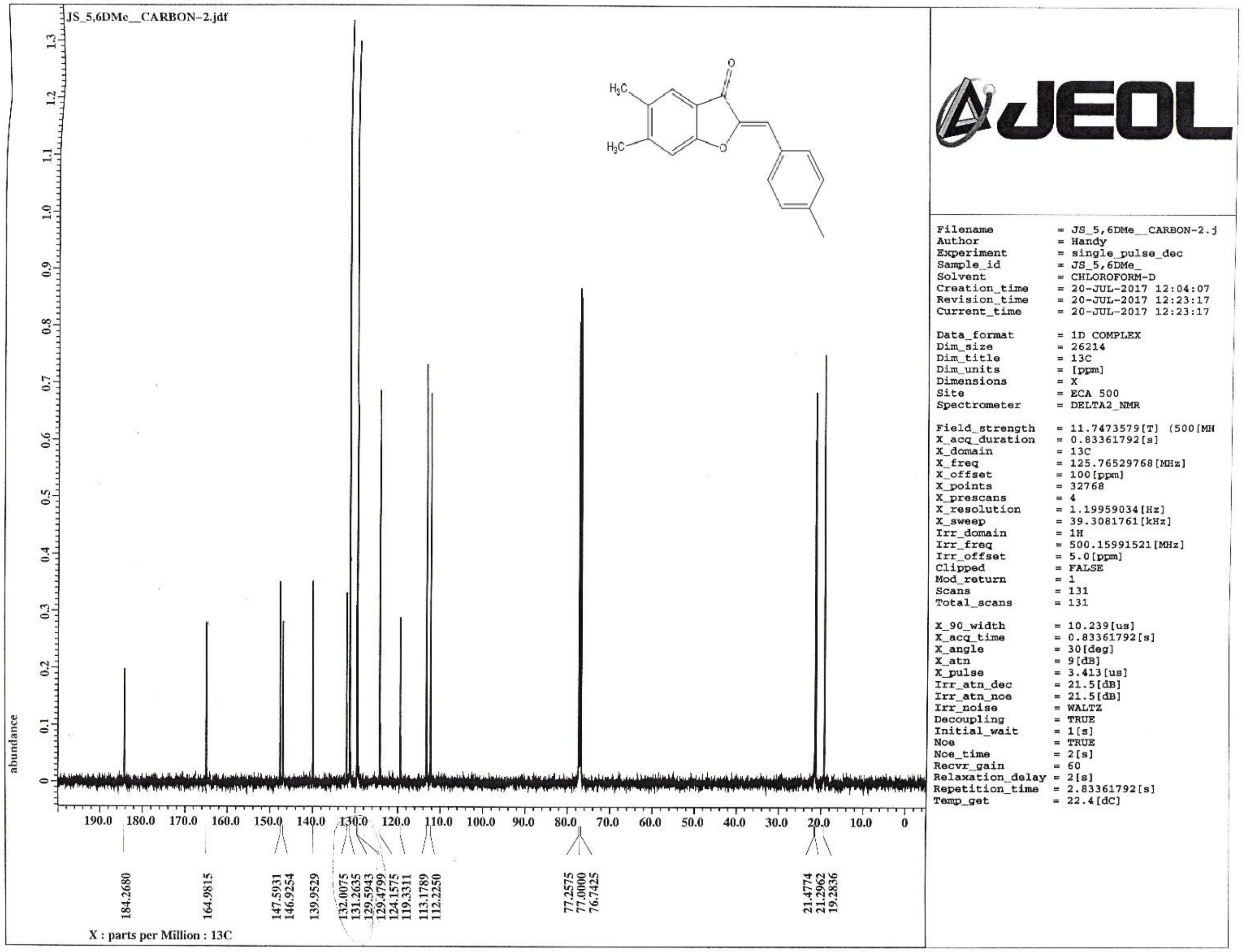
JS_5,6DMe__CARBON-2.jdf
abundance
X : parts per Million : 13C
184.2680
164.9815
147.5931
146.9254
139.9529
132.0075
131.2635
129.5943
129.4799
124.1575
119.3311
113.1789
112.2250
77.2575
77.0000
76.7425
21.4774
21.2962
19.2836
Filename = JS_5,6DMe__CARBON-2.j
Author = Handy
Experiment = single_pulse_dec
Sample_id = JS_5,6DMe_
Solvent = CHLOROFORM-D
Creation_time = 20-JUL-2017 12:04:07
Revision_time = 20-JUL-2017 12:23:17
Current_time = 20-JUL-2017 12:23:17
Data_format = 1D COMPLEX
Dim_size = 26214
Dim_title = 13C
Dim_units = [ppm]
Dimensions = X
Site = ECA 500
Spectrometer = DELTA2_NMR
Field_strength = 11.7473579[T] (500[MH
X_acq_duration = 0.83361792[s]
X_domain = 13C
X_freq = 125.76529768[MHz]
X_offset = 100[ppm]
X_points = 32768
X_prescans = 4
X_resolution = 1.19959034[Hz]
X_sweep = 39.3081761[kHz]
Irr_domain = 1H
Irr_freq = 500.15991521[MHz]
Irr_offset = 5.0[ppm]
Clipped = FALSE
Mod_return = 1
Scans = 131
Total_scans = 131
X_90_width = 10.239[us]
X_acq_time = 0.83361792[s]
X_angle = 30[deg]
X_atn = 9[dB]
X_pulse = 3.413[us]
Irr_atn_dec = 21.5[dB]
Irr_atn_noe = 21.5[dB]
Irr_noise = WALTZ
Decoupling = TRUE
Initial_wait = 1[s]
Noe = TRUE
Noe_time = 2[s]
Recvr_gain = 60
Relaxation_delay = 2[s]
Repetition_time = 2.83361792[s]
Temp_get = 22.4[dC]

C:\Sync\ShimadzuGCMS\Documents\Handy\Schmitt\5,6 dMe.qgd

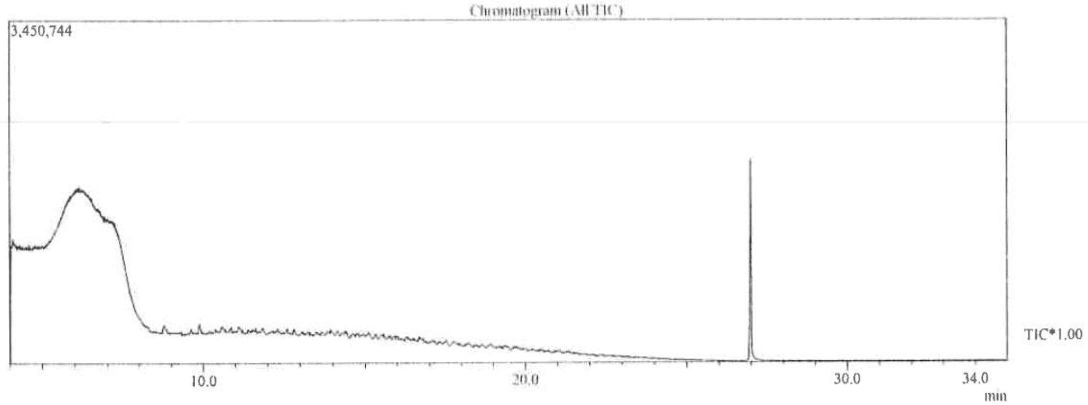

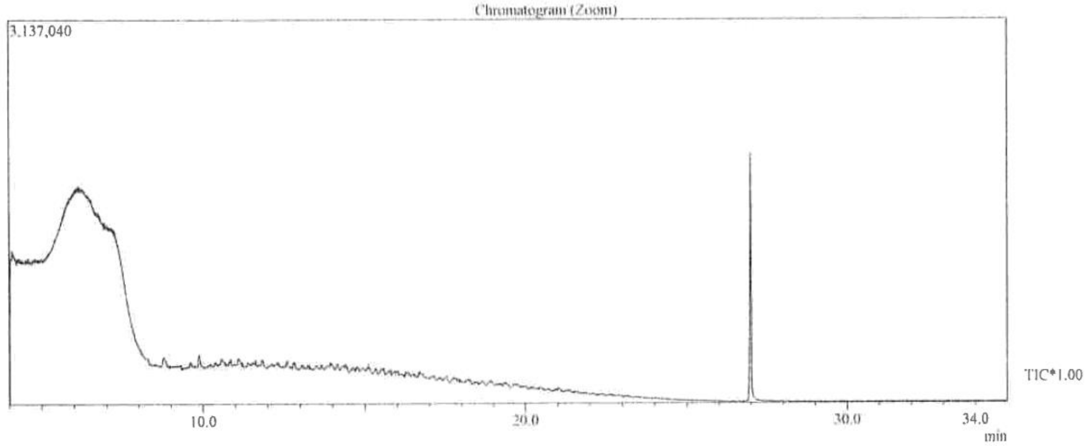

Spectrum

Line#:1 R.Time:27.015(Scan#:4604)
MassPeaks:64
RawMode:Single 27.015(4604) BasePeak:91.10(23252)
BG Mode:None Group 1 - Event 1 Scan

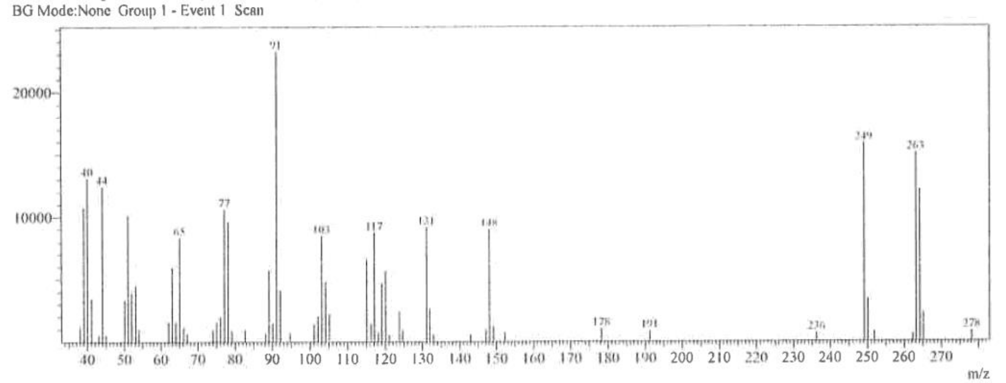

2-(4-methylbenzylidene)-1-(7'-methylbenzofuran-3-one) [**13**]

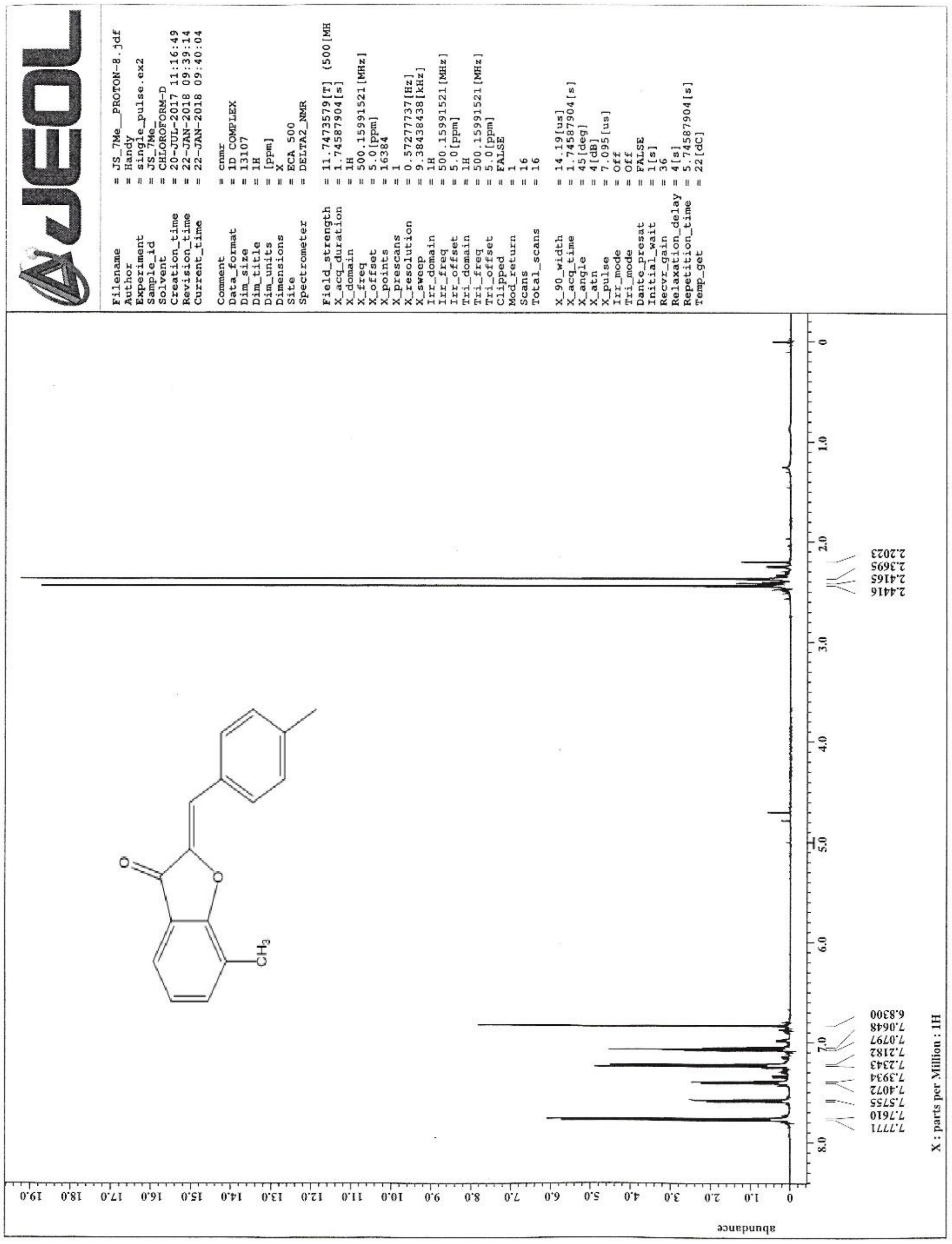

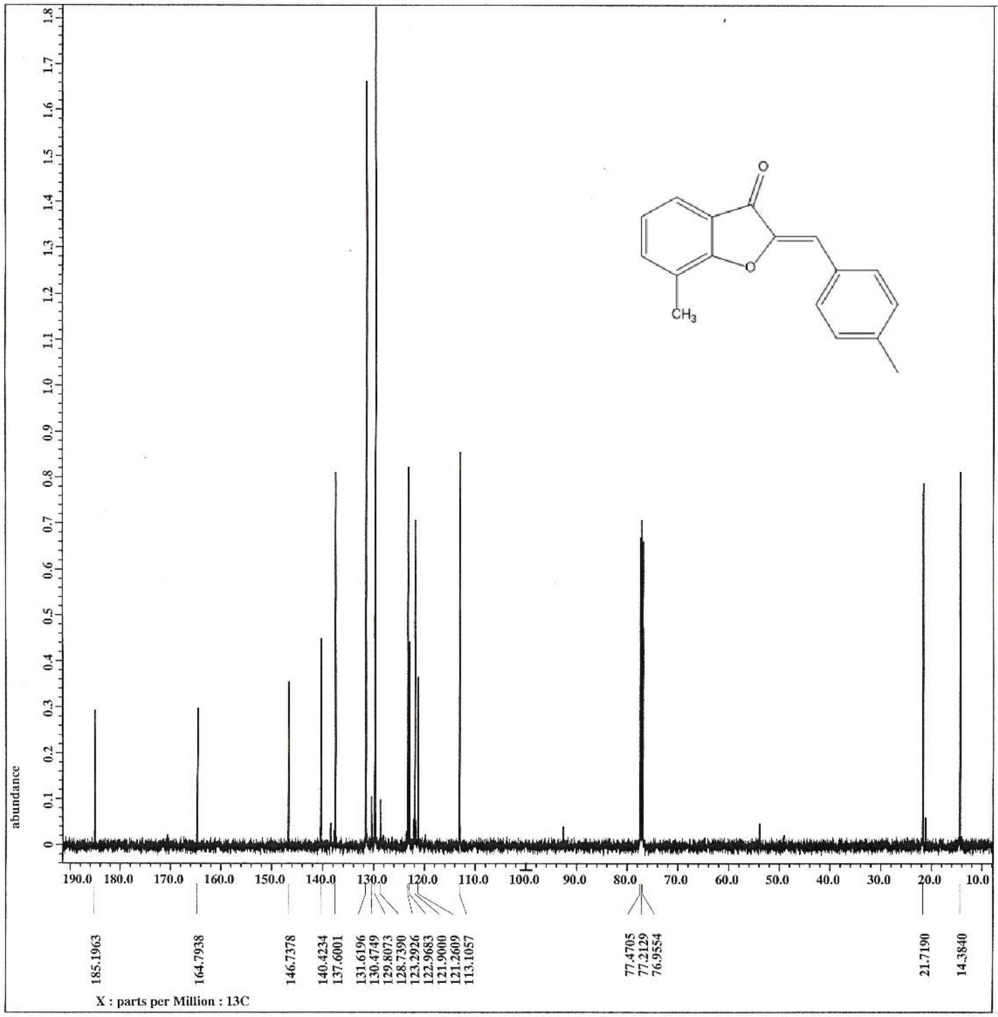

JEOL

```
Filename            = JS_7Me__CARBON-7.jdf
Author              = Handy
Experiment          = single_pulse_dec
Sample_id           = JS_7Me_
Solvent             = CHLOROFORM-D
Creation_time       = 20-JUL-2017 11:44:54
Revision_time       = 22-JAN-2018 09:42:05
Current_time        = 22-JAN-2018 09:43:36

Comment             = cnmr
Data_format         = 1D COMPLEX
Dim_size            = 26214
Dim_title           = 13C
Dim_units           = [ppm]
Dimensions          = X
Site                = ECA 500
Spectrometer        = DELTA2_NMR

Field_strength      = 11.7473579[T] (500[MH
X_acq_duration      = 0.83361792[s]
X_domain            = 13C
X_freq              = 125.76529768[MHz]
X_offset            = 100[ppm]
X_points            = 32768
X_prescans          = 4
X_resolution        = 1.19959034[Hz]
X_sweep             = 39.3081761[kHz]
Irr_domain          = 1H
Irr_freq            = 500.15991521[MHz]
Irr_offset          = 5.0[ppm]
Clipped             = FALSE
Mod_return          = 1
Scans               = 581
Total_scans         = 581

X_90_width          = 10.239[us]
X_acq_time          = 0.83361792[s]
X_angle             = 30[deg]
X_atn               = 9[dB]
X_pulse             = 3.413[us]
Irr_atn_dec         = 21.5[dB]
Irr_atn_noe         = 21.5[dB]
Irr_noise           = WALTZ
Decoupling          = TRUE
Initial_wait        = 1[s]
Noe                 = TRUE
Noe_time            = 2[s]
Recvr_gain          = 60
Relaxation_delay    = 2[s]
Repetition_time     = 2.83361792[s]
Temp_get            = 22.4[dC]
```

C:\Sync\ShimadzuGCMS\Documents\Handy\Schmitt\7Me.qgd

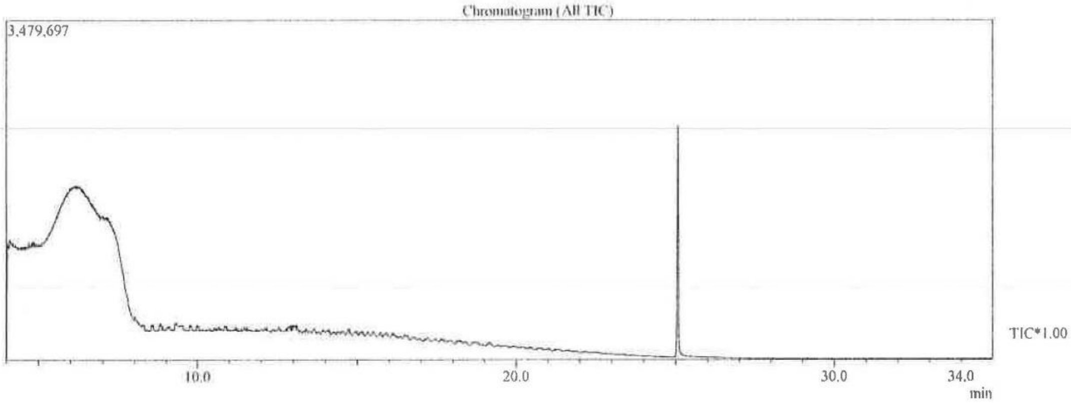

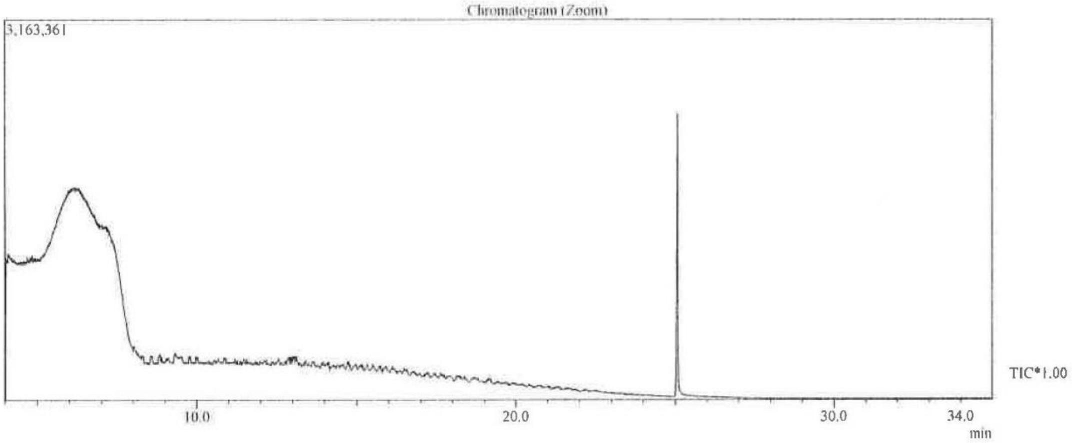

Spectrum

Line#:1 R.Time:25.080(Scan#:4217)
MassPeaks:135
RawMode:Single 25.080(4217) BasePeak:235.10(206370)
BG Mode:None Group 1 - Event 1 Scan

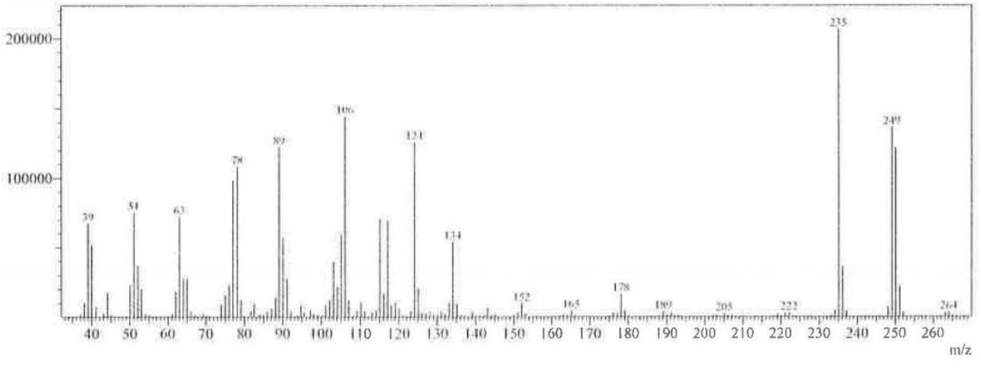

2-(4-methylbenzylidene)-1-(6’-hydroxybenzofuran-3-one) [**14**]

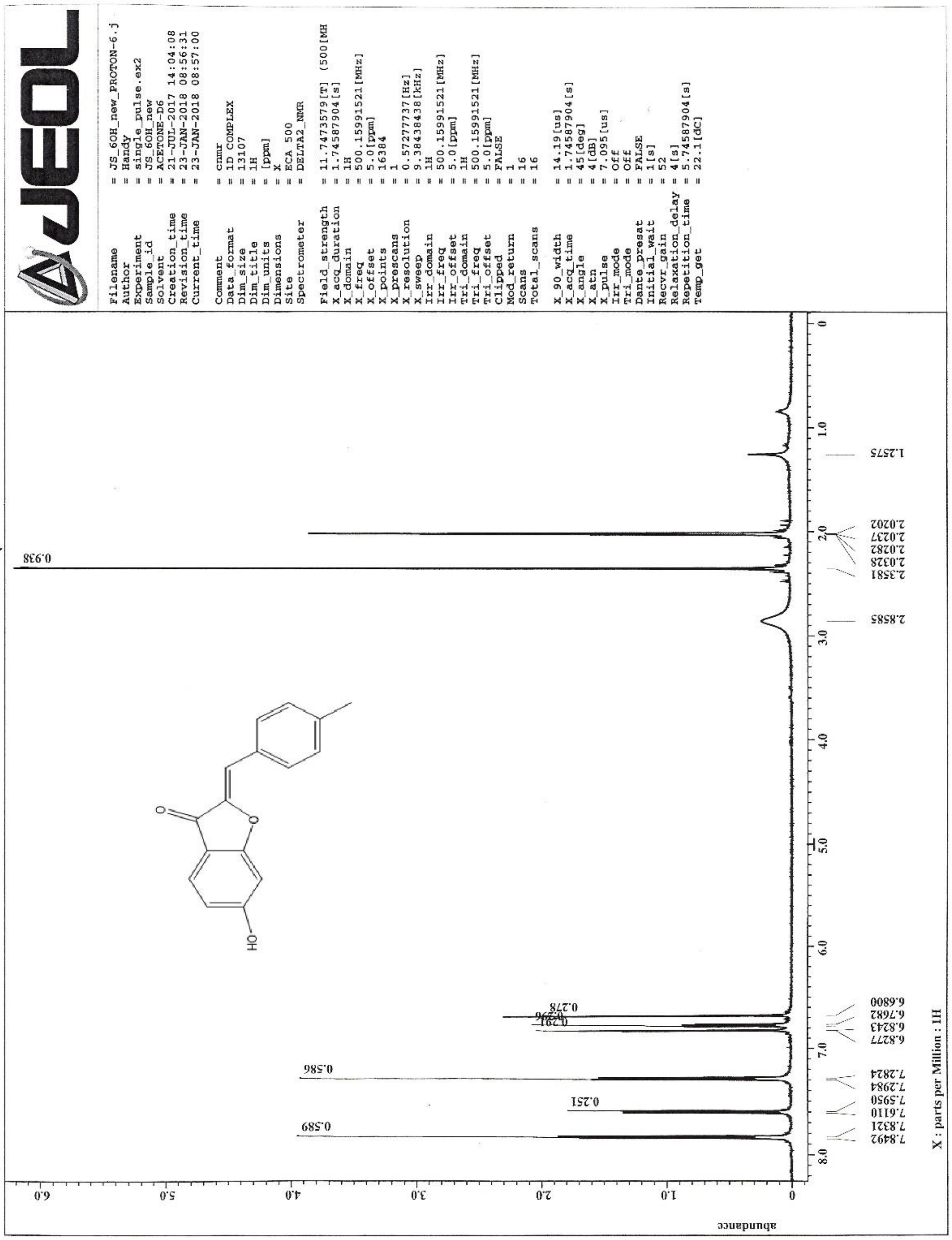

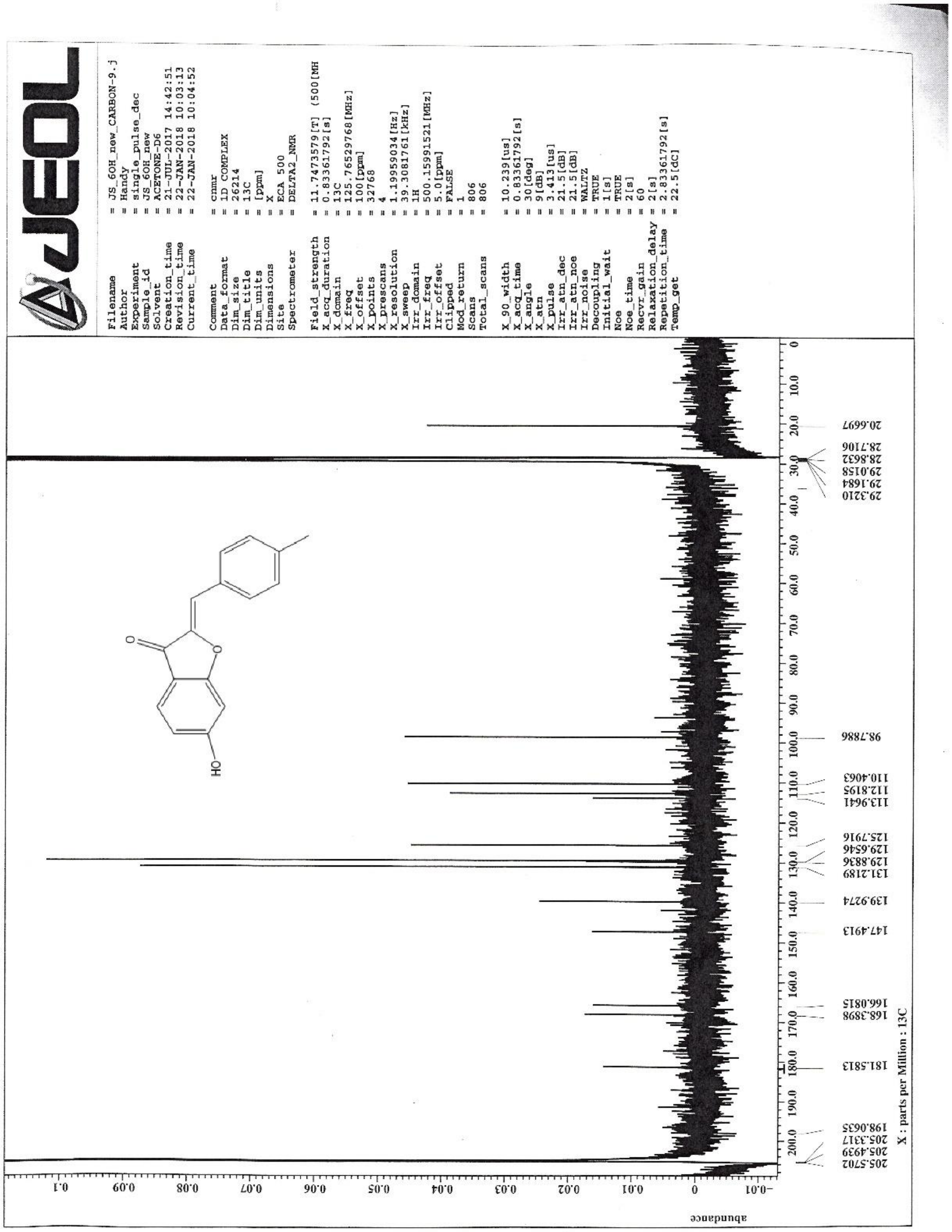
JEOL
Filename = JS_6OH_new_CARBON-9.j
Author = Handy
Experiment = single_pulse_dec
Sample_id = JS_6OH_new
Solvent = ACETONE-D6
Creation_time = 21-JUL-2017 14:42:51
Revision_time = 22-JAN-2018 10:03:13
Current_time = 22-JAN-2018 10:04:52
Comment = cnmr
Data_format = 1D COMPLEX
Dim_size = 26214
Dim_title = 13C
Dim_units = [ppm]
Dimensions = X
Site = ECA 500
Spectrometer = DELTA2_NMR
Field_strength = 11.7473579[T] (500[MH
X_acq_duration = 0.83361792[s]
X_domain = 13C
X_freq = 125.76529768[MHz]
X_offset = 100[ppm]
X_points = 32768
X_prescans = 4
X_resolution = 1.19959034[Hz]
X_sweep = 39.3081761[kHz]
Irr_domain = 1H
Irr_freq = 500.15991521[MHz]
Irr_offset = 5.0[ppm]
Clipped = FALSE
Mod_return = 1
Scans = 806
Total_scans = 806
X_90_width = 10.239[us]
X_acq_time = 0.83361792[s]
X_angle = 30[deg]
X_atn = 9[dB]
X_pulse = 3.413[us]
Irr_atn_dec = 21.5[dB]
Irr_atn_noe = 21.5[dB]
Irr_noise = WALTZ
Decoupling = TRUE
Initial_wait = 1[s]
Noe = TRUE
Noe_time = 2[s]
Recvr_gain = 60
Relaxation_delay = 2[s]
Repetition_time = 2.83361792[s]
Temp_get = 22.5[dC]
20.6697
28.7106
28.8632
29.0158
29.1684
29.3210
98.7886
110.4063
112.8195
113.9641
125.7916
129.6546
129.8836
131.2189
139.9274
147.4913
166.0815
168.3898
181.5813
198.0635
205.3317
205.4939
205.5702
OH
O
X : parts per Million : 13C
abundance

C:\Sync\ShimadzuGCMS\Documents\Handy\Schmitt\6OH.qgd

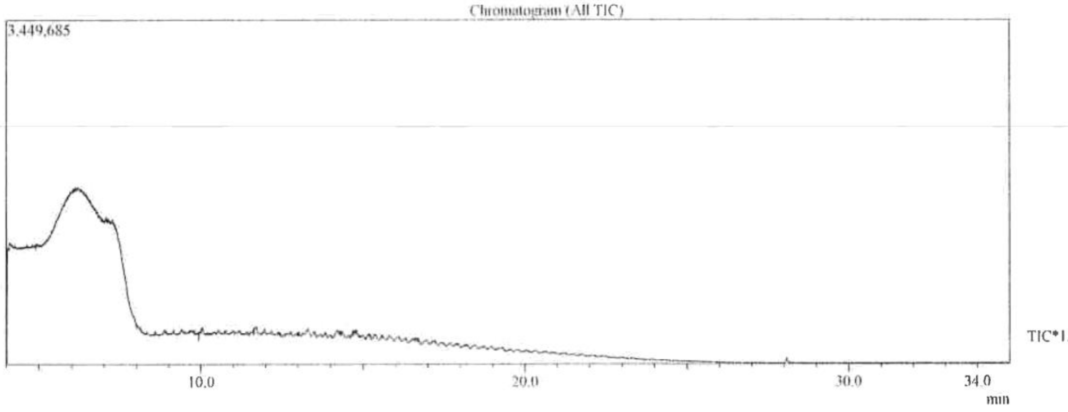

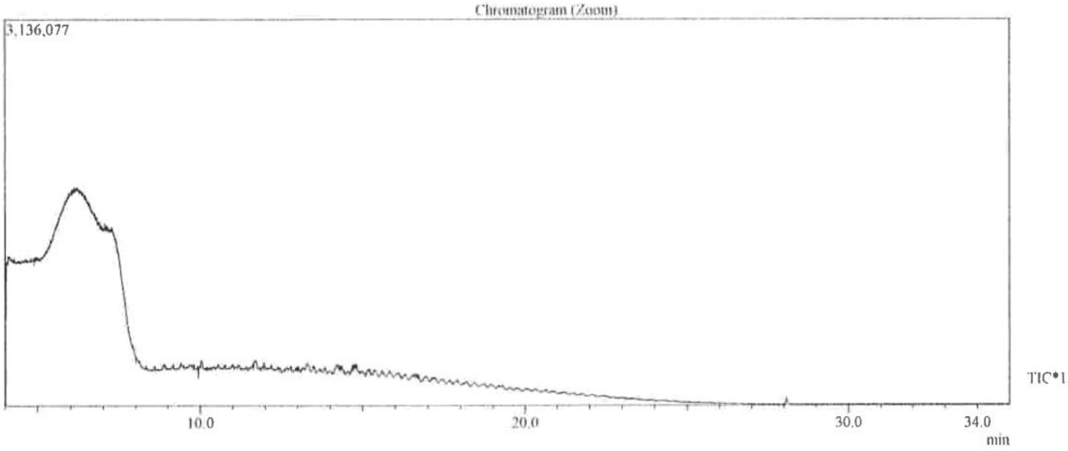

Spectrum

Line#:1 R.Time:28.090(Scan#:4819)
MassPeaks:27
RawMode:Single 28.090(4819) BasePeak 44.05(5186)
BG Mode:None Group 1 - Event 1 Scan

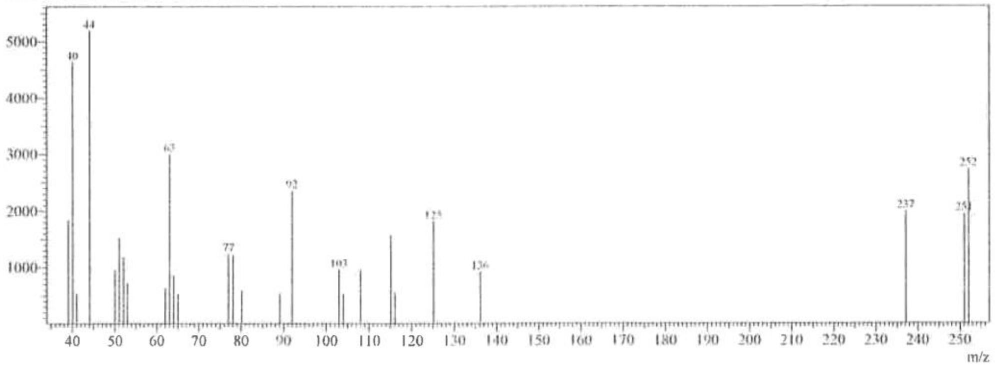

2-(4-methylbenzylidene)-1-(4’-hydroxybenzofuran-3-one) [**15**]

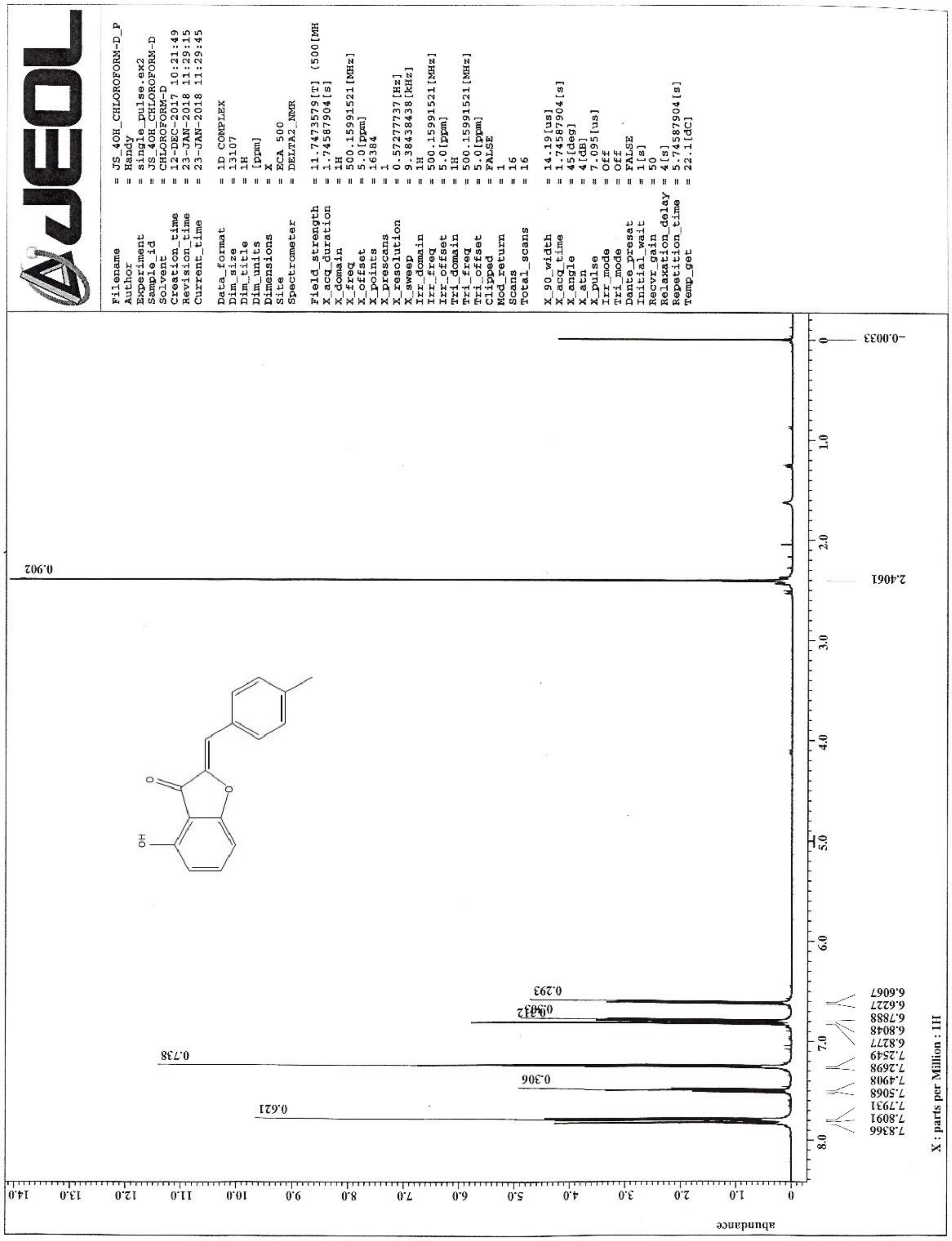

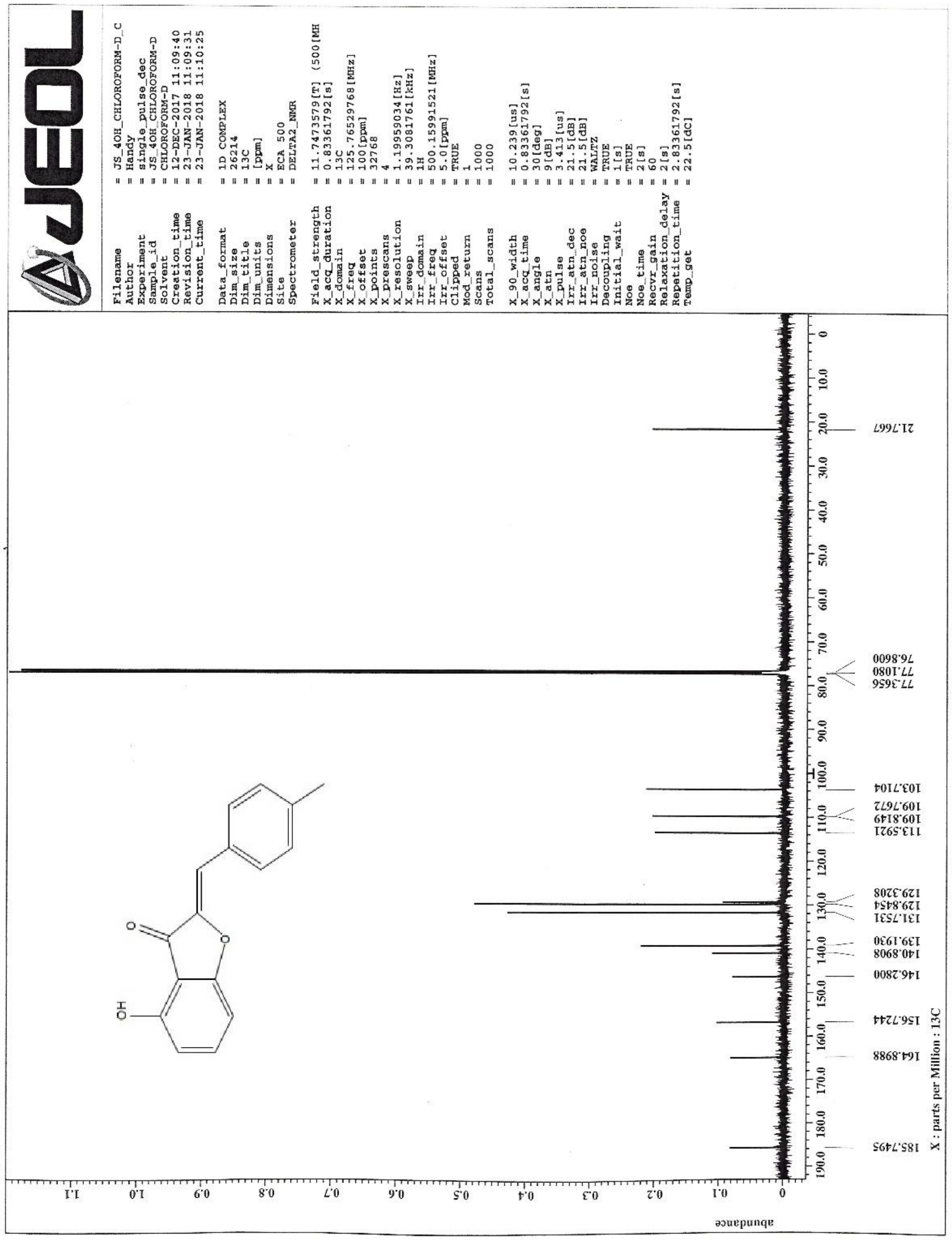
Filename = JS_4OH_CHLOROFORM-D_C
Author = Handy
Experiment = single_pulse_dec
Sample_id = JS_4OH_CHLOROFORM-D
Solvent = CHLOROFORM-D
Creation_time = 12-DEC-2017 11:09:40
Revision_time = 23-JAN-2018 11:09:31
Current_time = 23-JAN-2018 11:10:25
Data_format = 1D COMPLEX
Dim_size = 26214
Dim_title = 13C
Dim_units = [ppm]
Dimensions = X
Site = ECA 500
Spectrometer = DELTA2_NMR
Field_strength = 11.7473579[T] (500[MH
X_acq_duration = 0.83361792[s]
X_domain = 13C
X_freq = 125.76529768[MHz]
X_offset = 100[ppm]
X_points = 32768
X_prescans = 4
X_resolution = 1.19959034[Hz]
X_sweep = 39.3081761[kHz]
Irr_domain = 1H
Irr_freq = 500.15991521[MHz]
Irr_offset = 5.0[ppm]
Clipped = TRUE
Mod_return = 1
Scans = 1000
Total_scans = 1000
X_90_width = 10.239[us]
X_acq_time = 0.83361792[s]
X_angle = 30[deg]
X_atn = 9[dB]
X_pulse = 3.413[us]
Irr_atn_dec = 21.5[dB]
Irr_atn_noe = 21.5[dB]
Irr_noise = WALTZ
Decoupling = TRUE
Initial_wait = 1[s]
Noe = TRUE
Noe_time = 2[s]
Recvr_gain = 60
Relaxation_delay = 2[s]
Repetition_time = 2.83361792[s]
Temp_get = 22.5[dC]
185.7495
164.8988
156.7244
146.2800
140.8908
139.1930
131.7531
129.8454
129.3208
113.5921
109.8149
109.7672
103.7104
77.3656
77.1080
76.8600
21.7667
X : parts per Million : 13C
abundance
OH
O

C:\Sync\ShimadzuGCMS\Documents\Handy\Schmitt\4OH001.qgd

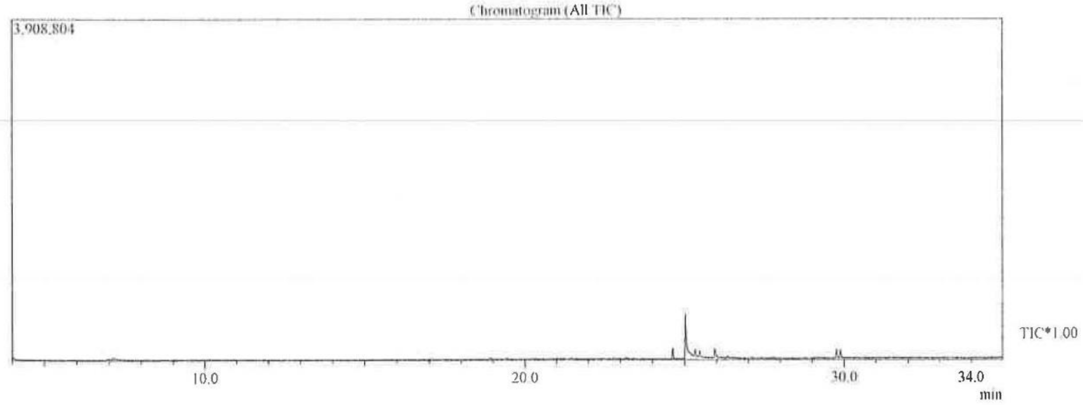

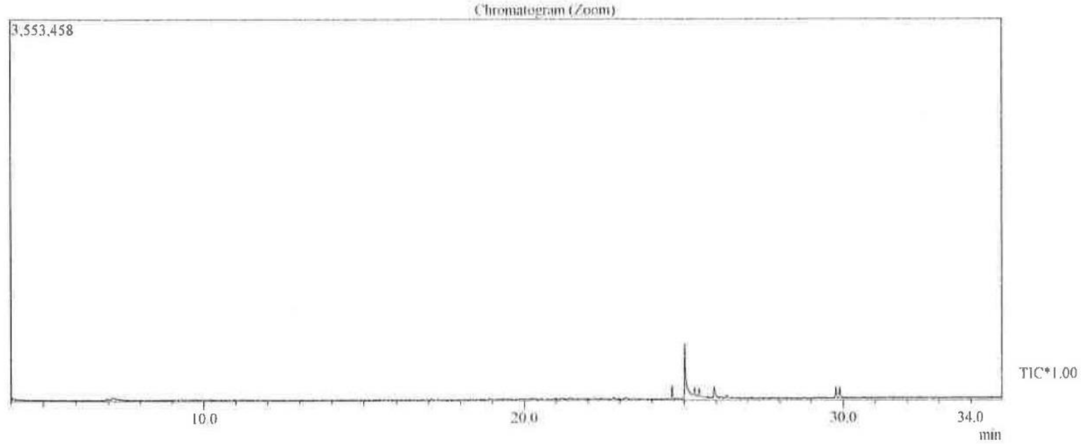

Spectrum

Line#:1 R.Time:25.035(Scan#:4208)
MassPeaks:95
RawMode:Single 25.035(4208) BasePeak:236.90(38782)
BG Mode:None Group 1 - Event 1 Scan

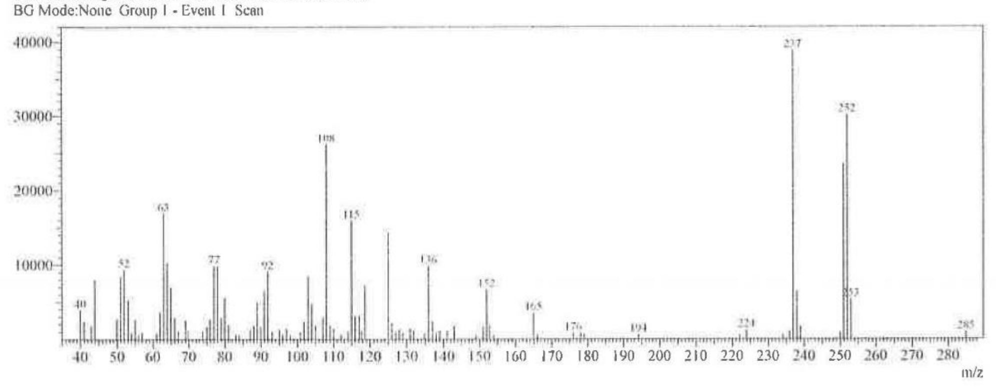

2-(4-methylbenzylidene)-1-(6’-hydroxy-4’-methylbenzofuran-3-one) [**16**]

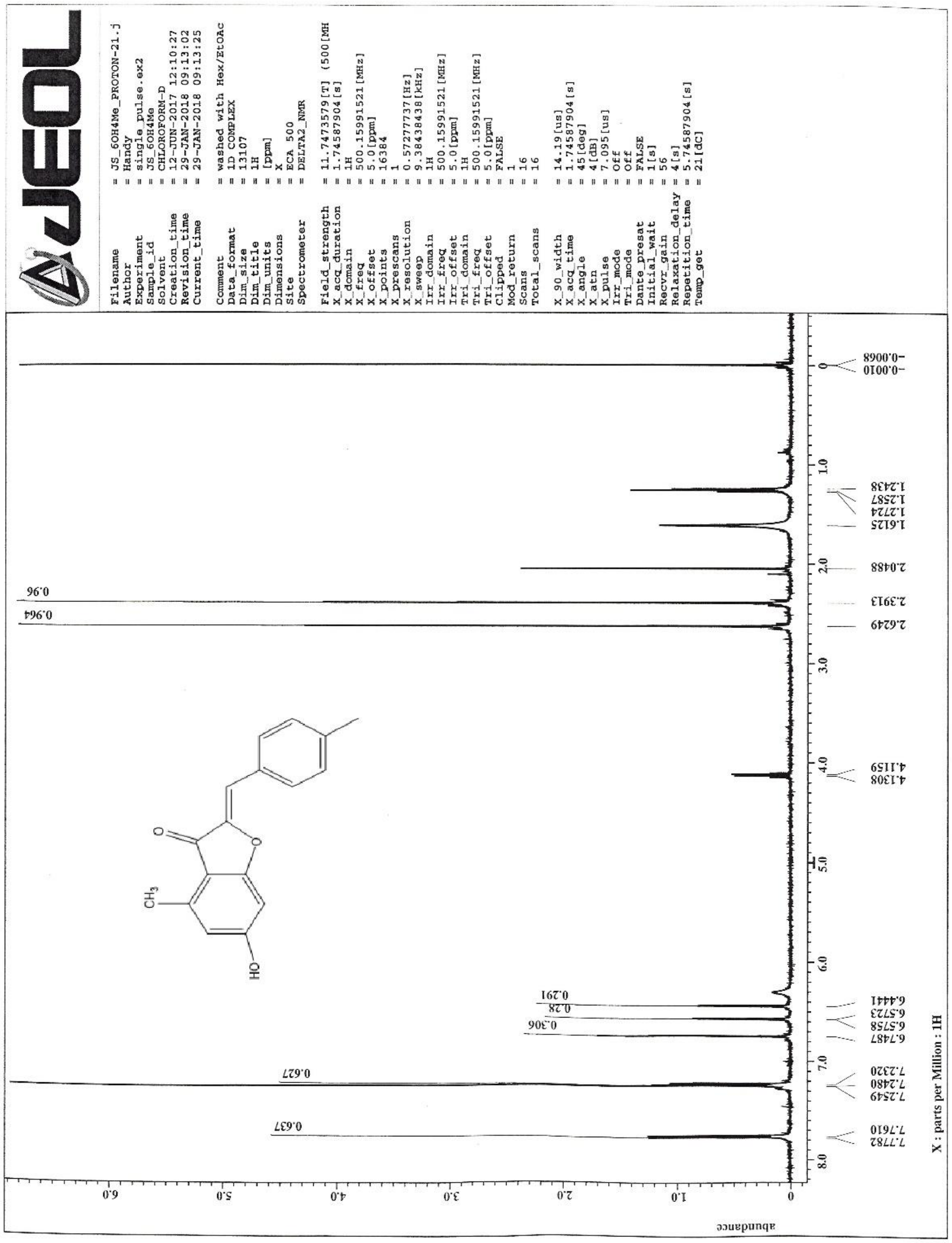

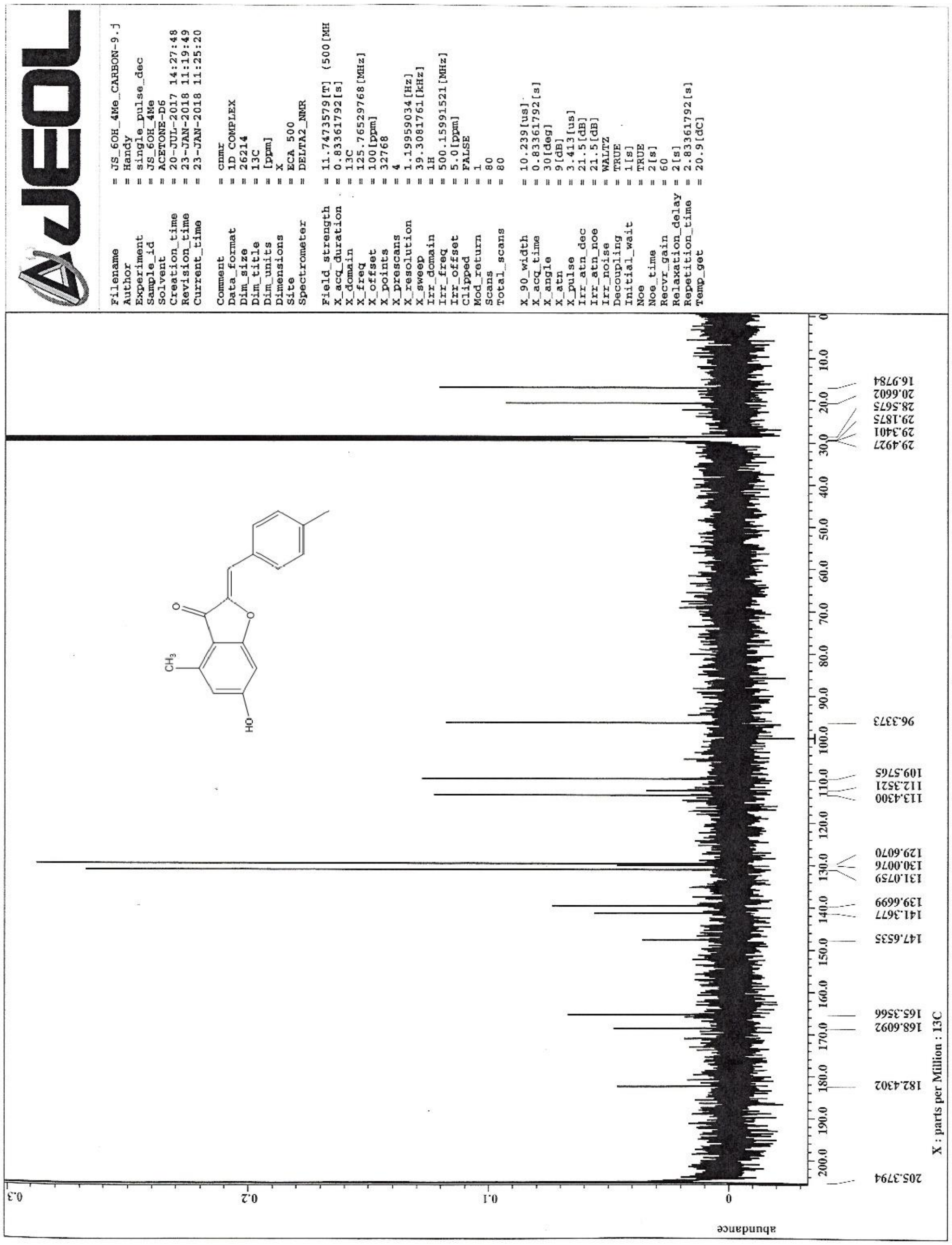
JEOL
Filename = JS_6OH_4Me_CARBON-9.jdf
Author = Handy
Experiment = single_pulse_dec
Sample_id = JS_6OH_4Me
Solvent = ACETONE-D6
Creation_time = 20-JUL-2017 14:27:48
Revision_time = 23-JAN-2018 11:19:49
Current_time = 23-JAN-2018 11:25:20
Comment = cnmr
Data_format = 1D COMPLEX
Dim_size = 26214
Dim_title = 13C
Dim_units = [ppm]
Dimensions = X
Site = ECA 500
Spectrometer = DELTA2_NMR
Field_strength = 11.7473579[T] (500[MH
X_acq_duration = 0.83361792[s]
X_domain = 13C
X_freq = 125.76529768[MHz]
X_offset = 100[ppm]
X_points = 32768
X_prescans = 4
X_resolution = 1.19959034[Hz]
X_sweep = 39.3081761[kHz]
Irr_domain = 1H
Irr_freq = 500.15991521[MHz]
Irr_offset = 5.0[ppm]
Clipped = FALSE
Mod_return = 1
Scans = 80
Total_scans = 80
X_90_width = 10.239[us]
X_acq_time = 0.83361792[s]
X_angle = 30[deg]
X_atn = 9[dB]
X_pulse = 3.413[us]
Irr_atn_dec = 21.5[dB]
Irr_atn_noe = 21.5[dB]
Irr_noise = WALTZ
Decoupling = TRUE
Initial_wait = 1[s]
Noe = TRUE
Noe_time = 2[s]
Recvr_gain = 60
Relaxation_delay = 2[s]
Repetition_time = 2.83361792[s]
Temp_get = 20.9[dC]
CH3
HO
O
205.3794
182.4302
168.6092
165.3566
147.6535
141.3677
139.6699
131.0759
130.0076
129.6070
113.4300
112.3521
109.5765
96.3373
29.4927
29.3401
29.1875
28.5675
20.6602
16.9784
X : parts per Million : 13C
abundance
0
0.1
0.2
0.3

C:\Sync\ShimadzuGCMS\Documents\Handy\Schmitt\6OH4Me.qgd

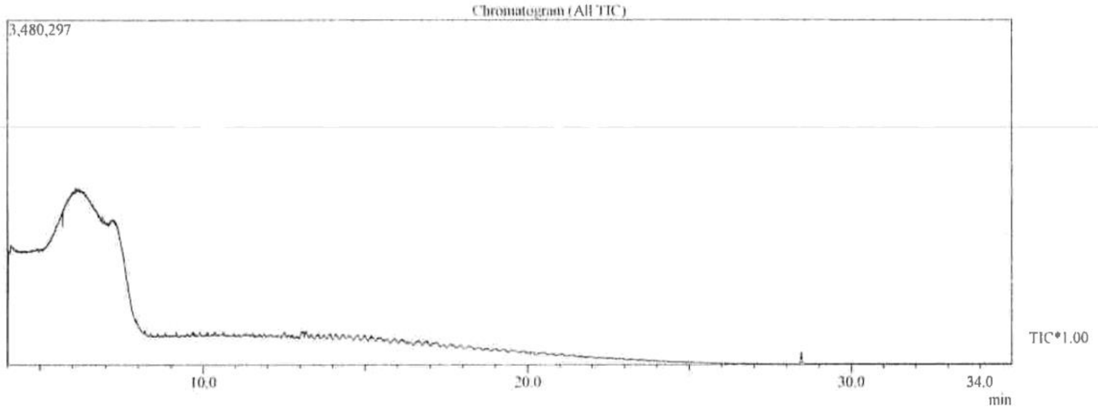

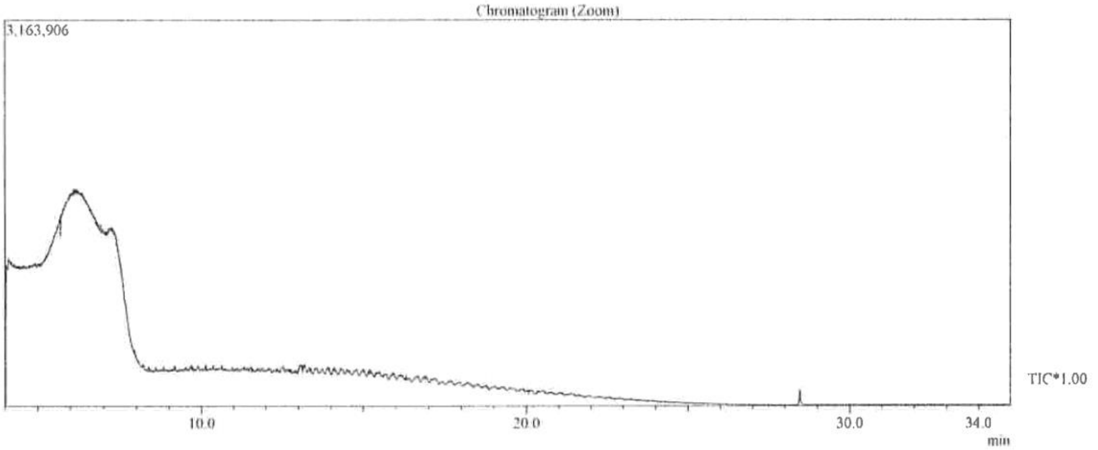

Spectrum

Line#:1 R.Time:28.475(Scan#:4896)
MassPeaks:32
RawMode:Single 28.475(4896) BasePeak:44.05(5575)
BG Mode:None Group 1 - Event 1 Scan

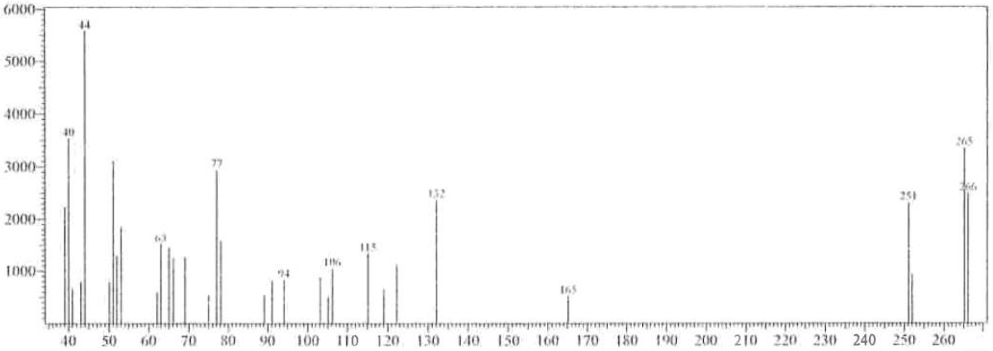

2-(4-methylbenzylidene)-1-(6’-hydroxy-7’-methylbenzofuran-3-one) [**17**]

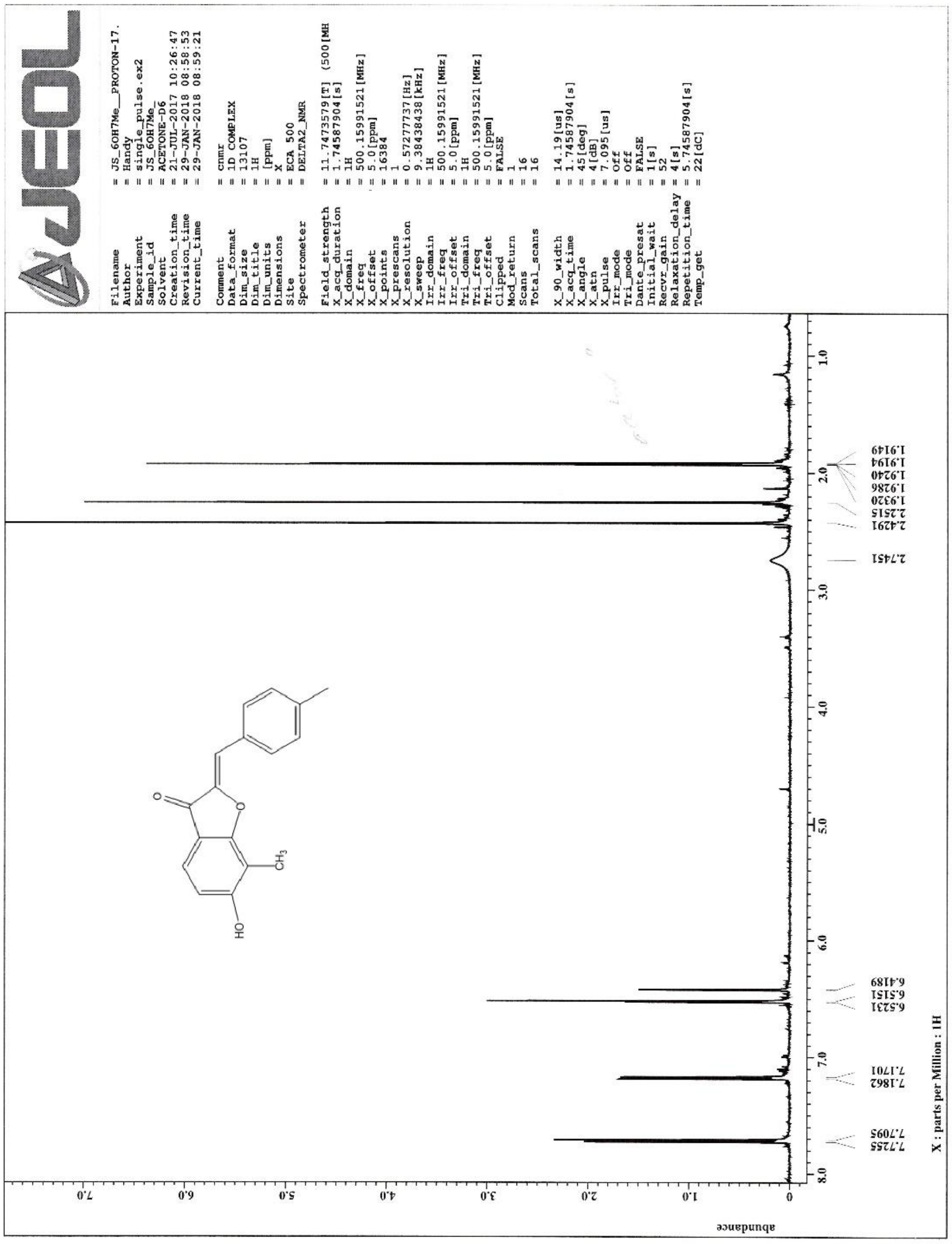

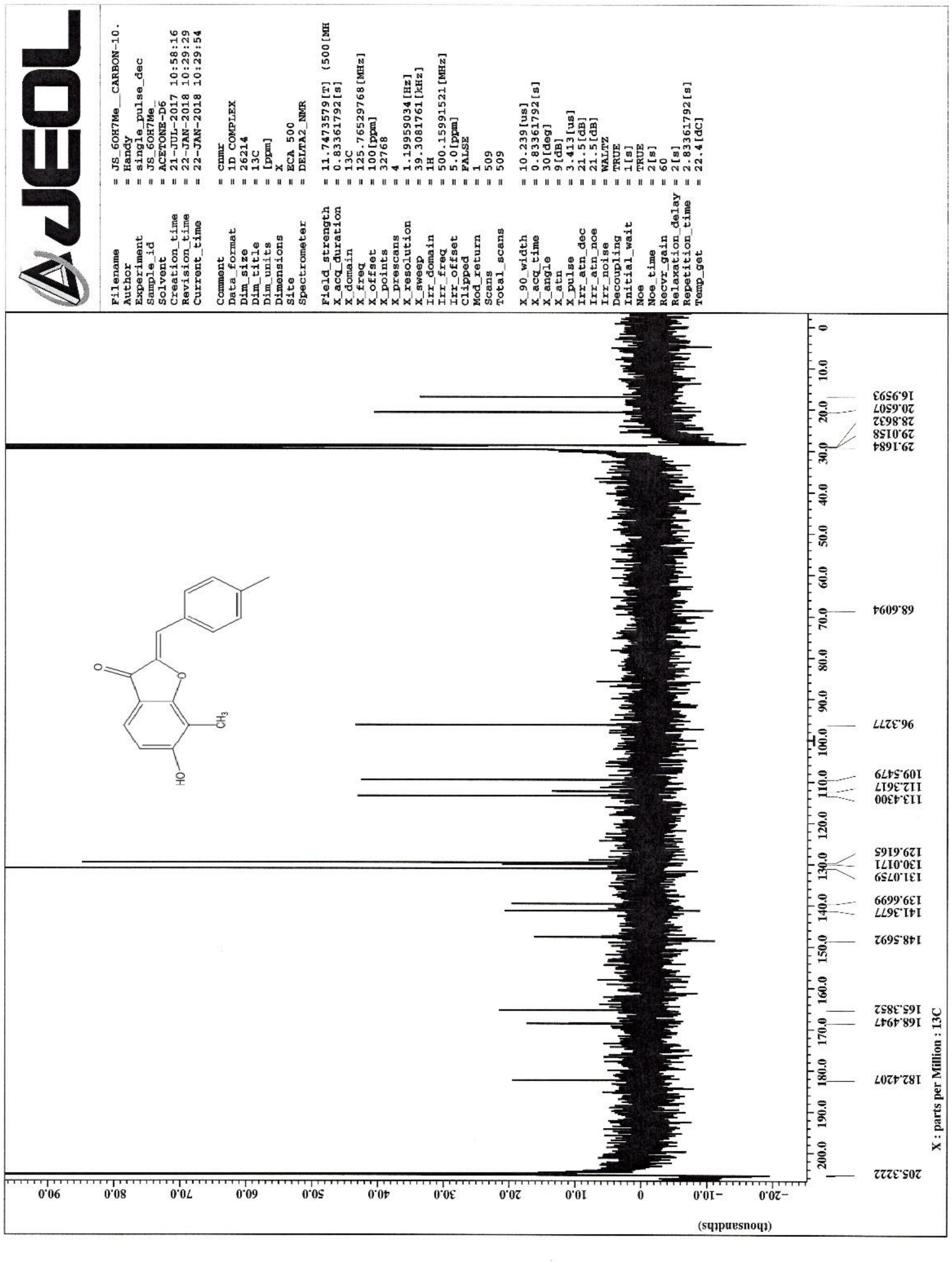

C:\Sync\ShimadzuGCMS\Documents\Handy\Schmitt\6OH7Me.qgd

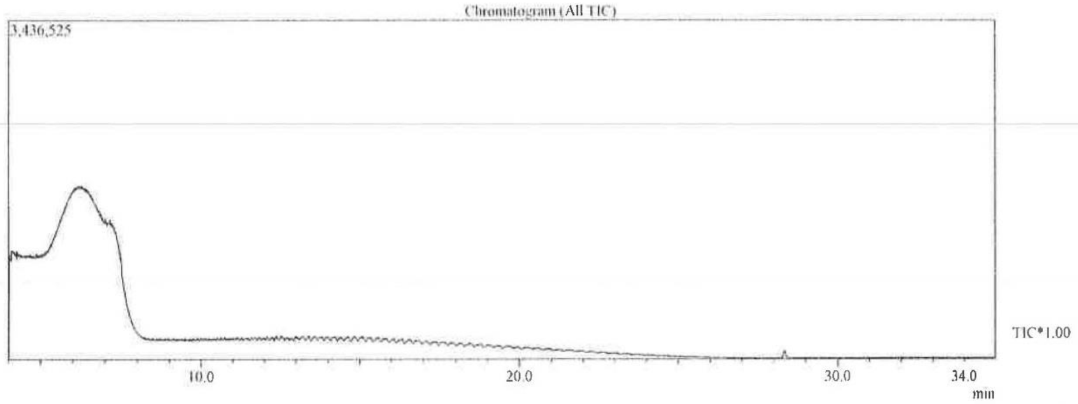

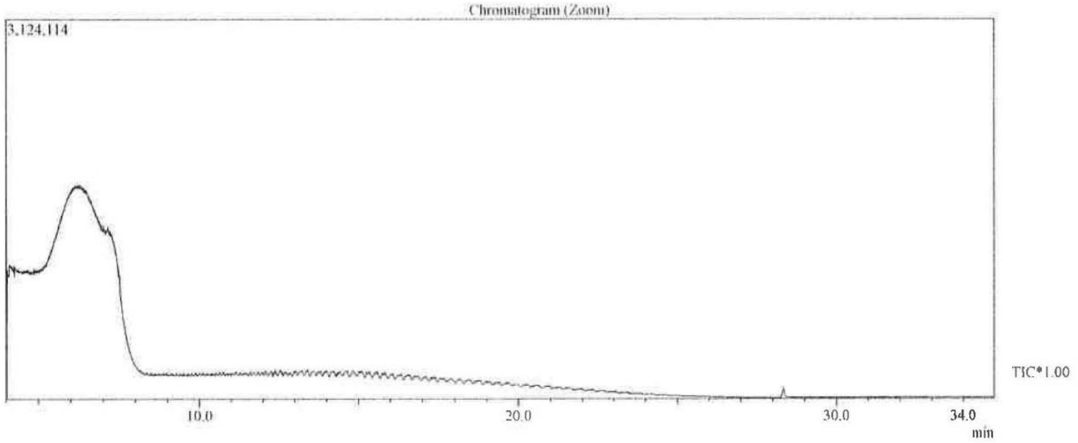

Spectrum

Line#:1 R.Time:28.360(Scan#:4873)
MassPeaks:36
RawMode:Single 28.360(4873) BasePeak:44.05(9778)
BG Mode:None Group 1 - Event 1 Scan

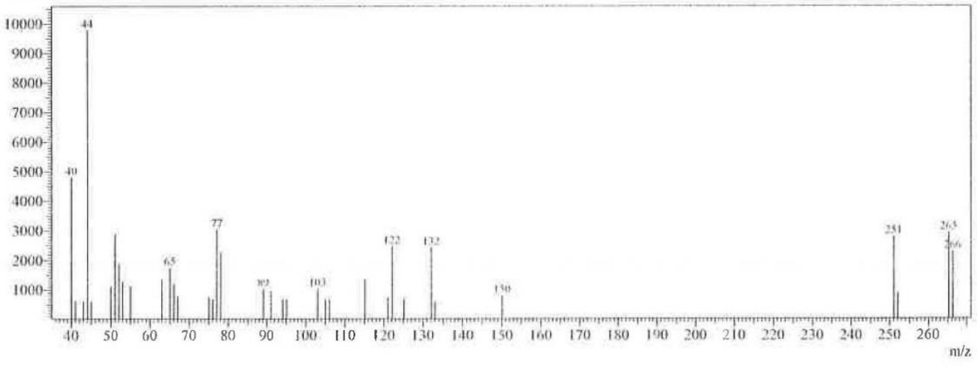

2-(4-methylbenzylidene)-1-(benzofuran-3-one) [**1**]

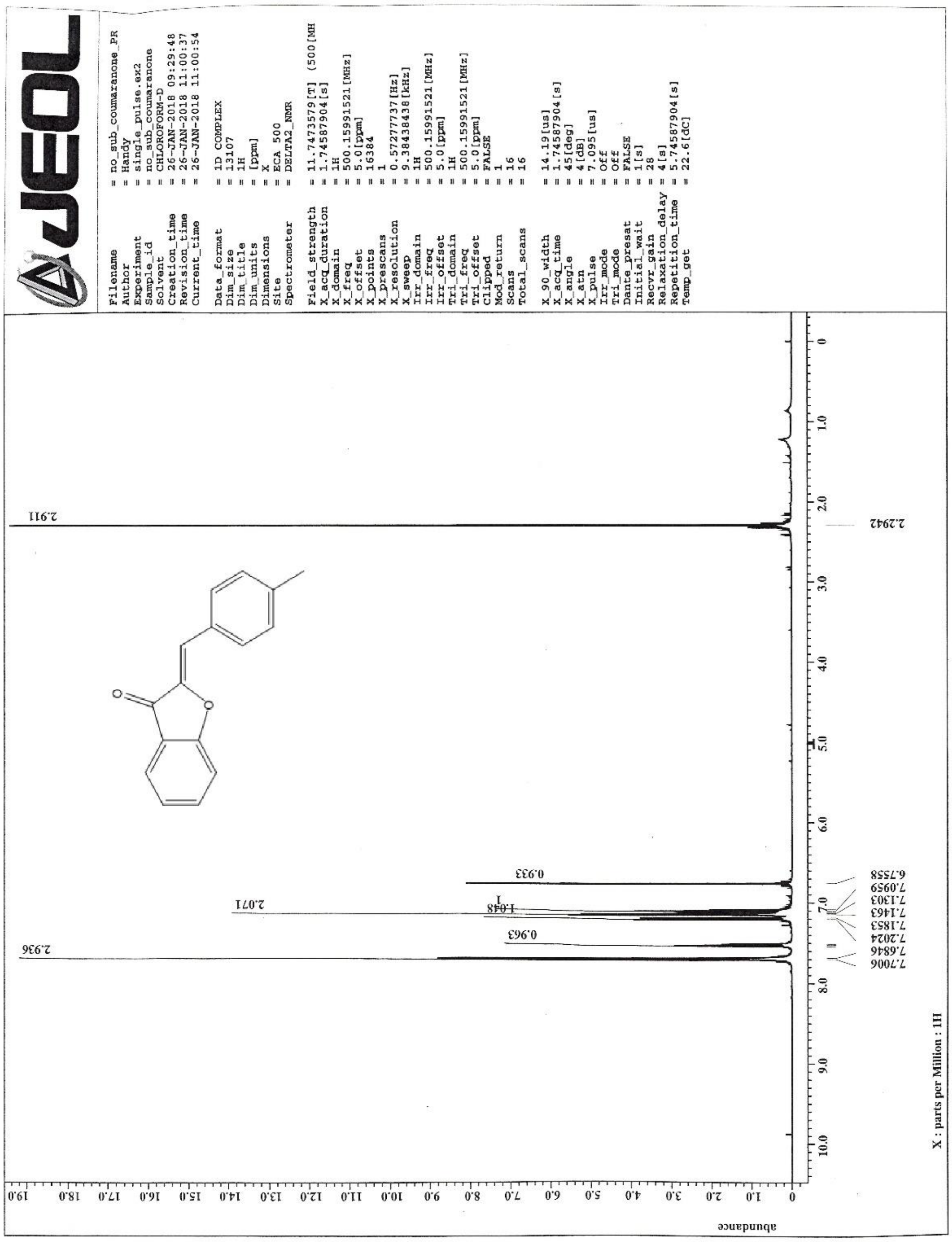

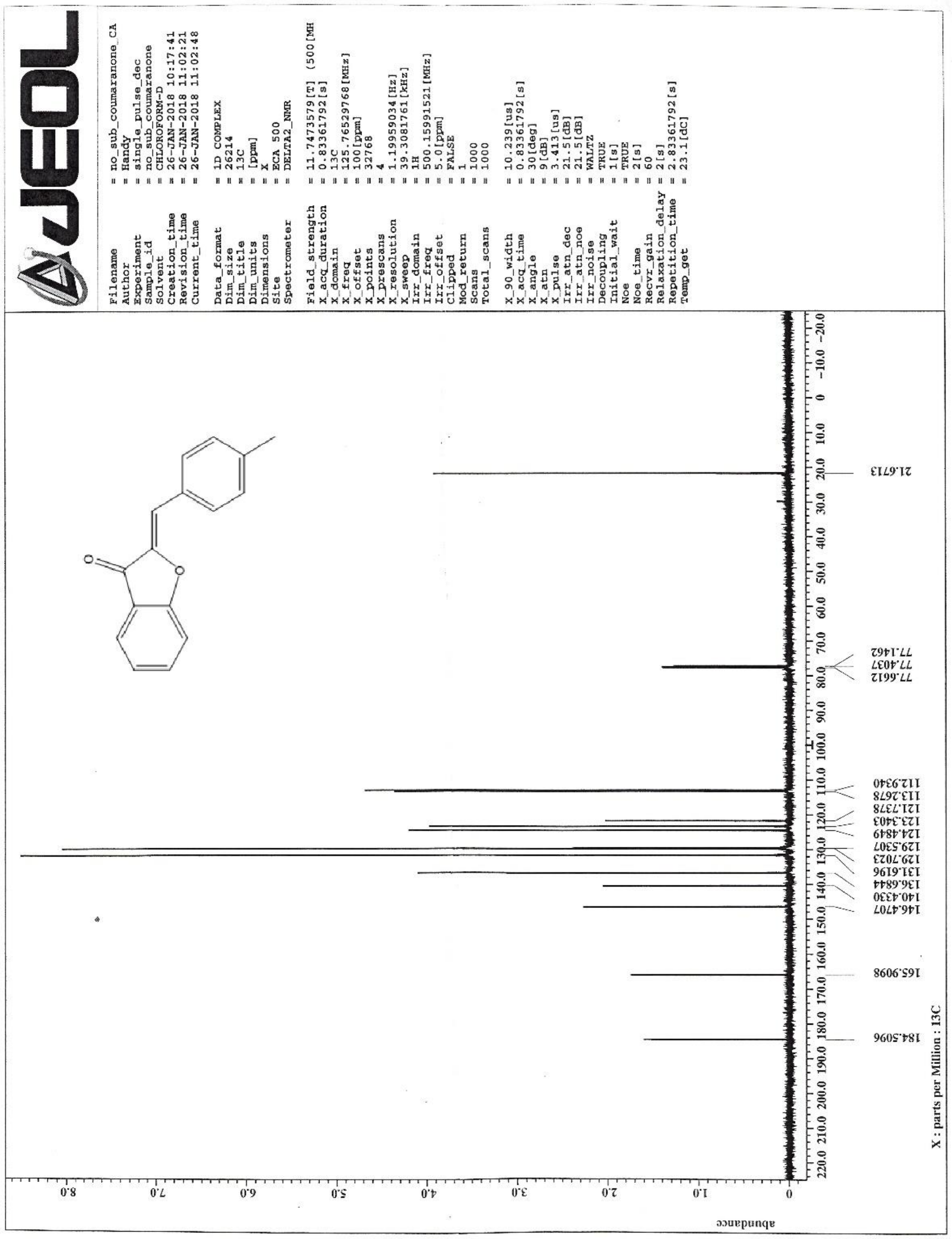
JEOL
Filename = no_sub_coumaranone_CA
Author = Handy
Experiment = single_pulse_dec
Sample_id = no_sub_coumaranone
Solvent = CHLOROFORM-D
Creation_time = 26-JAN-2018 10:17:41
Revision_time = 26-JAN-2018 11:02:21
Current_time = 26-JAN-2018 11:02:48
Data_format = 1D COMPLEX
Dim_size = 26214
Dim_title = 13C
Dim_units = [ppm]
Dimensions = X
Site = ECA 500
Spectrometer = DELTA2_NMR
Field_strength = 11.7473579[T] (500[MH
X_acq_duration = 0.83361792[s]
X_domain = 13C
X_freq = 125.76529768[MHz]
X_offset = 100[ppm]
X_points = 32768
X_prescans = 4
X_resolution = 1.19959034[Hz]
X_sweep = 39.3081761[kHz]
Irr_domain = 1H
Irr_freq = 500.15991521[MHz]
Irr_offset = 5.0[ppm]
Clipped = FALSE
Mod_return = 1
Scans = 1000
Total_scans = 1000
X_90_width = 10.239[us]
X_acq_time = 0.83361792[s]
X_angle = 30[deg]
X_atn = 9[dB]
X_pulse = 3.413[us]
Irr_atn_dec = 21.5[dB]
Irr_atn_noe = 21.5[dB]
Irr_noise = WALTZ
Decoupling = TRUE
Initial_wait = 1[s]
Noe = TRUE
Noe_time = 2[s]
Recvr_gain = 60
Relaxation_delay = 2[s]
Repetition_time = 2.83361792[s]
Temp_get = 23.1[dC]
21.6713
77.6612
77.4037
77.1462
112.9340
113.2678
121.7378
123.3403
124.4849
129.5307
129.7023
131.6196
136.6844
140.4330
146.4707
165.9098
184.5096
X : parts per Million : 13C
abundance

C:\Sync\ShimadzuGCMS\Documents\Handy\Schmitt\no sub in DCM.qgd

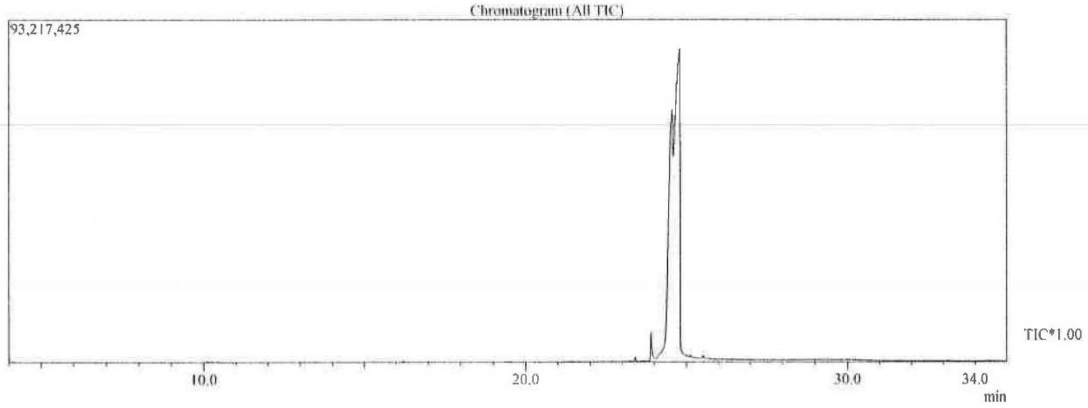

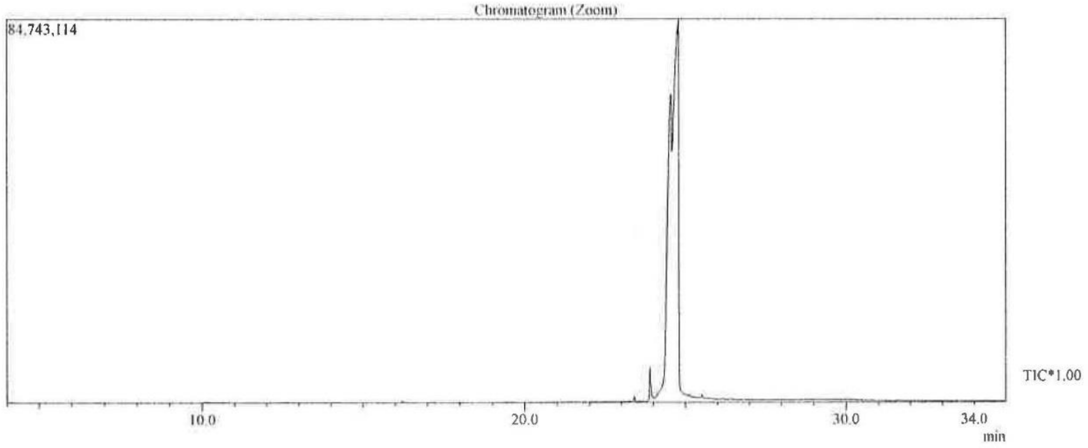

Spectrum

Line#:1 R.Time:24.745(Scan#:4150)
MassPeaks:185
RawMode:Single 24.745(4150) BasePeak:220.95(8428992)
BG Mode:None Group 1 - Event 1 Scan

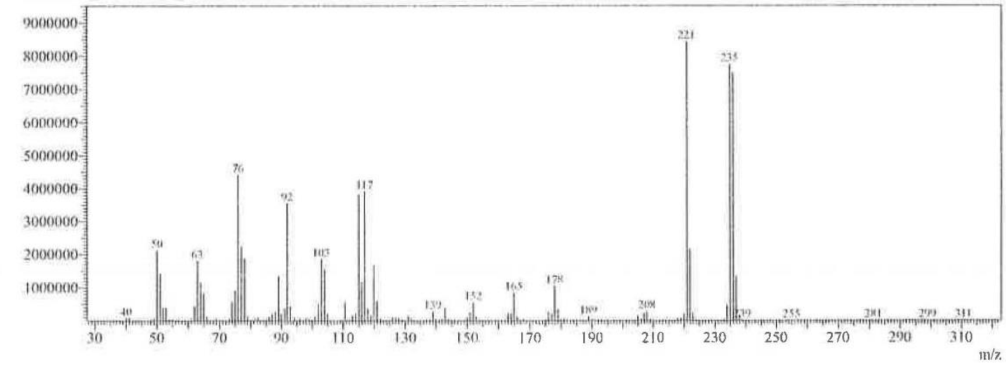

Supplement: File 1 — Experimental methods and spectra for all new compounds. [file Beilstein_J_Org_Chem-15-1781-s001.pdf]
